# Supplementary material for: Mapping PTBP2 binding in human brain identifies SYNGAP1 as a target for therapeutic splice switching
Source: Nat Commun. 2023 May 6;14:2628. doi: 10.1038/s41467-023-38273-3 (PMC10164156; doi:10.1038/s41467-023-38273-3)
Supplement: Supplementary file 1 — Supplementary Info [file 41467_2023_38273_MOESM1_ESM.pdf]

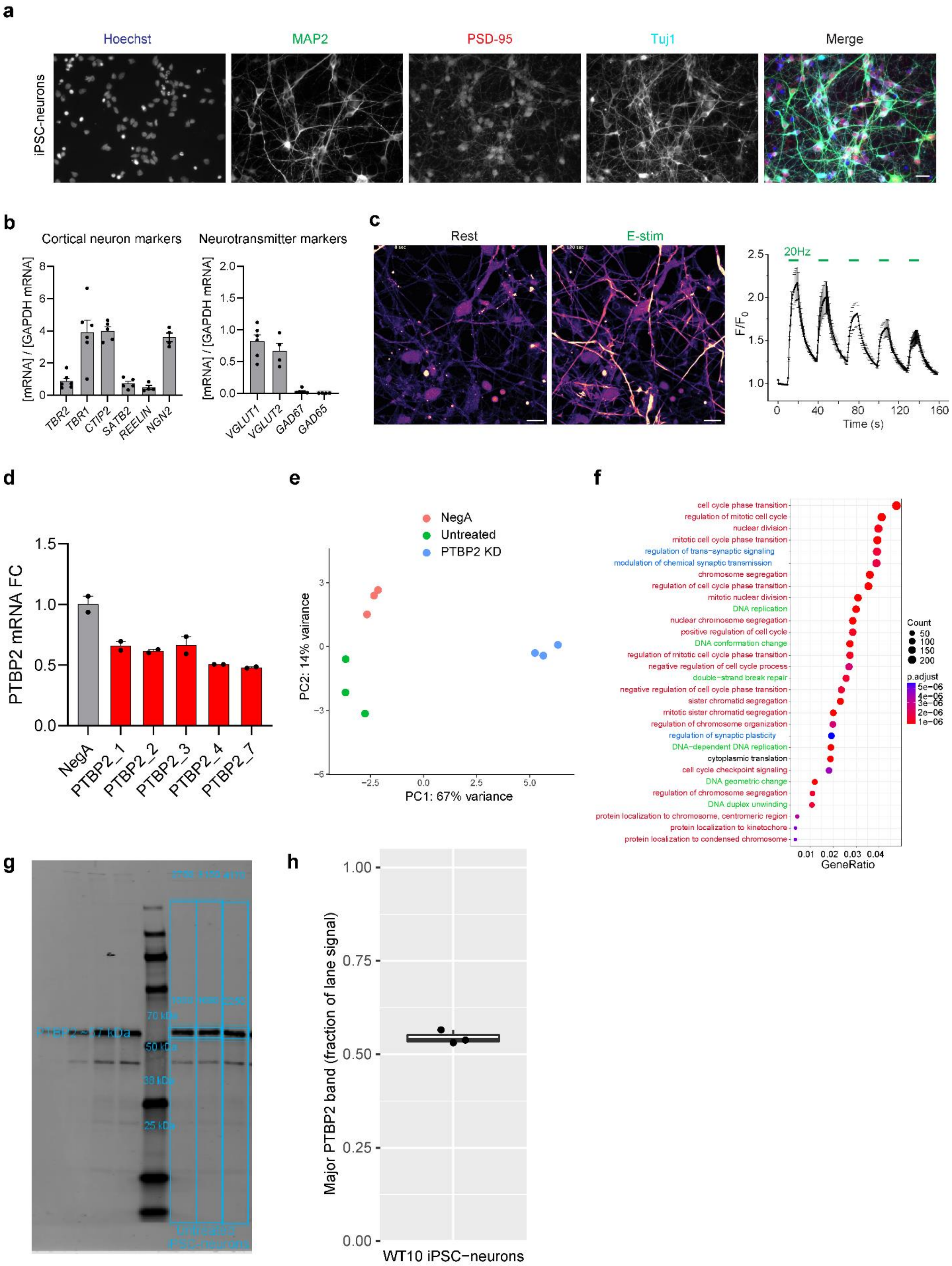
a
Hoechst
MAP2
PSD-95
Tuj1
Merge
iPSC-neurons
b
Cortical neuron markers
Neurotransmitter markers
[mRNA] / [GAPDH mRNA]
TBR2
TBR1
CTIP2
SATB2
REELIN
NGN2
VGLUT1
VGLUT2
GAD67
GAD65
c
Rest
E-stim
20Hz
F/F0
Time (s)
d
PTBP2 mRNA FC
NegA
PTBP2_1
PTBP2_2
PTBP2_3
PTBP2_4
PTBP2_7
e
NegA
Untreated
PTBP2 KD
PC2: 14% vairance
PC1: 67% variance
f
cell cycle phase transition
regulation of mitotic cell cycle
nuclear division
mitotic cell cycle phase transition
regulation of trans−synaptic signaling
modulation of chemical synaptic transmission
chromosome segregation
regulation of cell cycle phase transition
mitotic nuclear division
DNA replication
nuclear chromosome segregation
positive regulation of cell cycle
DNA conformation change
regulation of mitotic cell cycle phase transition
negative regulation of cell cycle process
double−strand break repair
negative regulation of cell cycle phase transition
sister chromatid segregation
mitotic sister chromatid segregation
regulation of chromosome organization
regulation of synaptic plasticity
DNA−dependent DNA replication
cytoplasmic translation
cell cycle checkpoint signaling
DNA geometric change
regulation of chromosome segregation
DNA duplex unwinding
protein localization to chromosome, centromeric region
protein localization to kinetochore
protein localization to condensed chromosome
Count
p.adjust
GeneRatio
g
70 kDa
50 kDa
38 kDa
25 kDa
h
Major PTBP2 band (fraction of lane signal)
WT10 iPSC−neurons

Supplementary Figure 1. Validation of iPSC-derived cortical neurons and analysis of PTBP2 knockdown in HEK293T cells and iPSC-neurons. (**a**) Neuronal marker expression by immunofluoresence of iPSC-neurons. iPSC-neurons were immunostained with antibodies specific to MAP2 (green), PSD-95 (red), and Tuj-1 (cyan), and counterstained with Hoechst-33342 (blue), indicative of neuronal phenotype. Scale bar = 20 µm. (**b**) (Left) qPCR of iPSC-neurons for transcripts of the excitatory cortical progenitor TBR2, and cortical neuron markers TBR1, CTIP2, SATB2, REELIN, and NGN2 showing expression of all subtypes. (Right) qPCR of iPSC-neurons identified transcripts of excitatory markers, VGLUT1 and VGLUT2, and minimal expression of inhibitory markers, GAD67 and GAD65. (**c**) Left panel, fluorescence images of iPSC-neurons loaded with a calcium indicator dye (Fluo-4 AM) at rest and electrically stimulated (E-stim) with 20 Hz trains of depolarizing field stimuli lasting 10 s, with 20 s of rest between trains. Scale bar = 10 µm. (Right) Quantification of fluorescence intensity changes over time when normalized to initial fluorescence levels (F/F0) using the 20 Hz stimulation. **(d)** qPCR from HEK293T cells transfected with 25 nM of PTBP2 gapmers for 24 h. A non-targeting gapmer (NegA) was included as negative control. **(e)** Principal component analysis (PCA) of gene-level rlog-transformed normalized count data from RNA-seq iPSC-neurons samples. **(f)** Dotplot (clusterProfiler) showing the top results from Gene Ontology (GO) enrichment analysis of genes differentially expressed upon PTBP2 KD (Biological Process, PTBP2 KD vs. untreated iPSC-neurons) relative to a background of all genes evaluated. Gene ratio is number of differentially expressed genes (padj < 0.05) relative to total genes in GO group (Count). **(g)** Primary characterization of PTBP2 antibody (EMD Millipore #ABE431) by Western blot in WT10 iPSC-neurons with molecular weight markers for three replicates (Chameleon Duo Ladder showing the 700 channel only, LI-COR 928-60000). PTBP2 predicted MW is 57.5 kDa (-/+ 20%: 46.0 kDa/69.0 kDa). The quantifications of the signal for the main band and the signal for the total lane are displayed (quantified regions and local background regions are indicated). **(h)** Quantification of **g** represented as a boxploxt. Fraction of signal from major PTBP2 band relative to the signal of the entire lane. In **b**-**d,** data are represented as mean values ± SEM. All data points represent independent biological replicates. **b** (*n* = 4-6 from 3-4 independent differentiations of the CHOP WT10 line). **c** (*n* = 4). **d** (*n* = 2). **e-h** (*n* = 3 biological replicates). Source data are provided as a Source Data file.

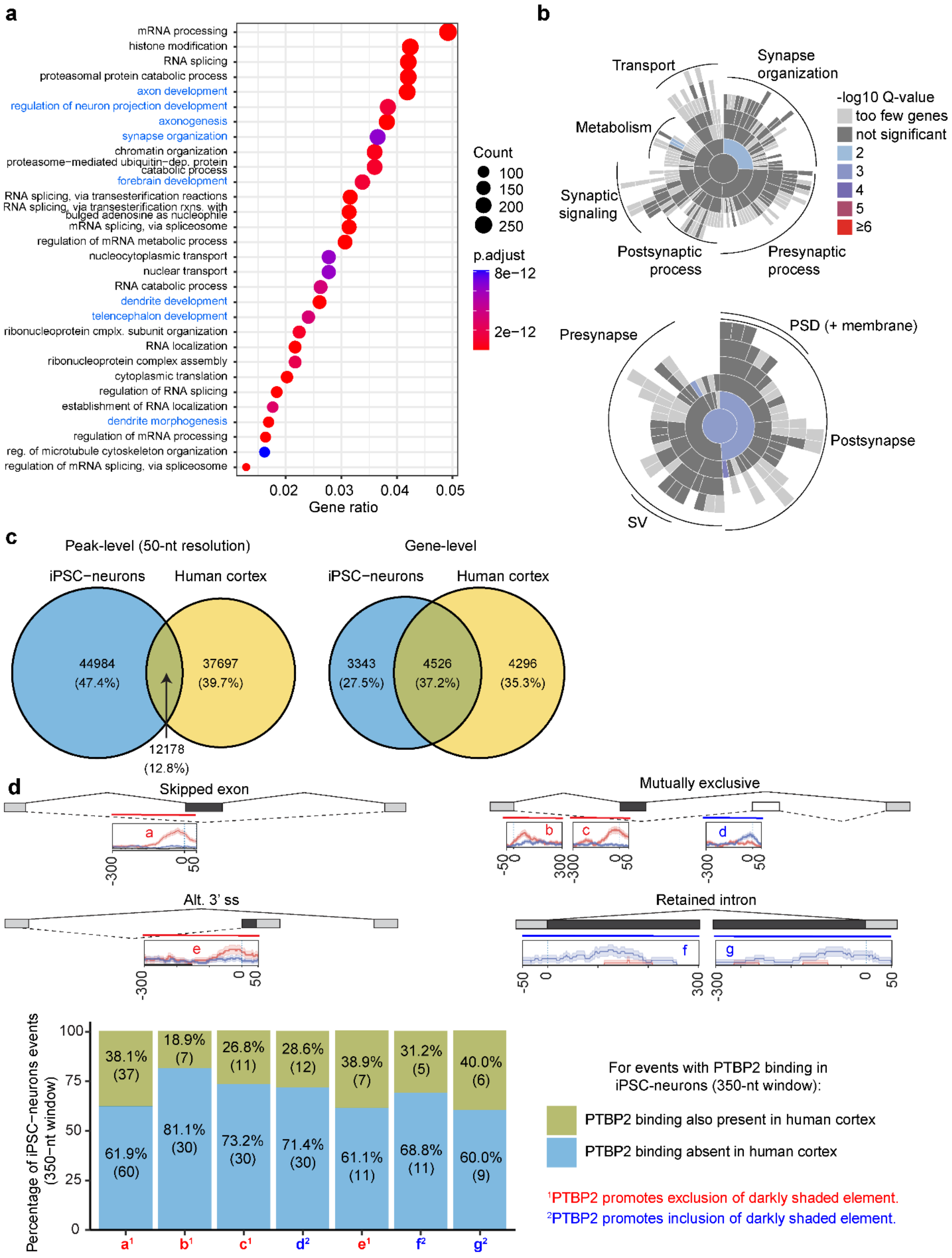

a
mRNA processing
histone modification
RNA splicing
proteasomal protein catabolic process
axon development
regulation of neuron projection development
axonogenesis
synapse organization
chromatin organization
proteasome−mediated ubiquitin−dep. protein catabolic process
forebrain development
RNA splicing, via transesterification reactions
RNA splicing, via transesterification rxns. with bulged adenosine as nucleophile
mRNA splicing, via spliceosome
regulation of mRNA metabolic process
nucleocytoplasmic transport
nuclear transport
RNA catabolic process
dendrite development
telencephalon development
ribonucleoprotein cmplx. subunit organization
RNA localization
ribonucleoprotein complex assembly
cytoplasmic translation
regulation of RNA splicing
establishment of RNA localization
dendrite morphogenesis
regulation of mRNA processing
reg. of microtubule cytoskeleton organization
regulation of mRNA splicing, via spliceosome
0.02 0.03 0.04 0.05
Gene ratio
Count
100
150
200
250
p.adjust
8e−12
2e−12
b
Transport
Synapse organization
Metabolism
Synaptic signaling
Postsynaptic process
Presynaptic process
-log10 Q-value
too few genes
not significant
2
3
4
5
≥6
Presynapse
PSD (+ membrane)
Postsynapse
SV
c
Peak-level (50-nt resolution)
iPSC−neurons
Human cortex
44984 (47.4%)
37697 (39.7%)
12178 (12.8%)
Gene-level
iPSC−neurons
Human cortex
3343 (27.5%)
4526 (37.2%)
4296 (35.3%)
d
Skipped exon
a
-300 0 50
Mutually exclusive
b
c
d
-50 0 300 -300 0 50 -300 0 50
Alt. 3' ss
e
-300 0 50
Retained intron
f
g
-50 0 300 -300 0 50
Percentage of iPSC−neurons events (350−nt window)
100 75 50 25 0
38.1% (37)
61.9% (60)
18.9% (7)
81.1% (30)
26.8% (11)
73.2% (30)
28.6% (12)
71.4% (30)
38.9% (7)
61.1% (11)
31.2% (5)
68.8% (11)
40.0% (6)
60.0% (9)
a1 b1 c1 d2 e1 f2 g2
For events with PTBP2 binding in iPSC-neurons (350-nt window):
PTBP2 binding also present in human cortex
PTBP2 binding absent in human cortex
1PTBP2 promotes exclusion of darkly shaded element.
2PTBP2 promotes inclusion of darkly shaded element.

Supplementary Figure 2. PTBP2 CLIP-seq target gene ontology. **(a)** Dotplot (clusterProfiler) showing the top 30 categories from Gene Ontology (GO) enrichment analysis (Biological Process) of PTBP2 CLIP-seq peaks in iPSC-neurons. Gene ratio is number of genes with peak calls relative to total genes in Gene Ontology group (Count). Synapse-related terms are highlighted in blue. **(b)** SynGO enrichment analysis of PTBP2 CLIP-seq peaks in iPSC-neurons relative to a background set of brain-expressed genes represented as a sunburst plot. (Top) Biological Process, 442 genes. (Bottom) Cellular Component, 572 genes. **(c)** Venn diagram of PTBP2 binding in iPSC-neurons and human cortex at the (left) 50-nt resolution level of CLIP-seq peak calls and (right) at the gene level. **(d)** PTBP2 binding within 350 nucleotides proximal to AS events in iPSC-neurons. (Top) schematic of AS events showing positioning of 350-nt windows (see **Fig. 3f**). (Bottom) Percentage (and number) of events with PTBP2 binding in the given 350-nt window in iPSC-neurons that also showed PTBP2 binding in the same window in human cortex. *n* = 3 biological replicates for PTBP2 CLIP-seq and size-matched input controls. Source data are provided as a Source Data file. PSD, postsynaptic density; SV, synaptic vesicle; nt, nucleotide.

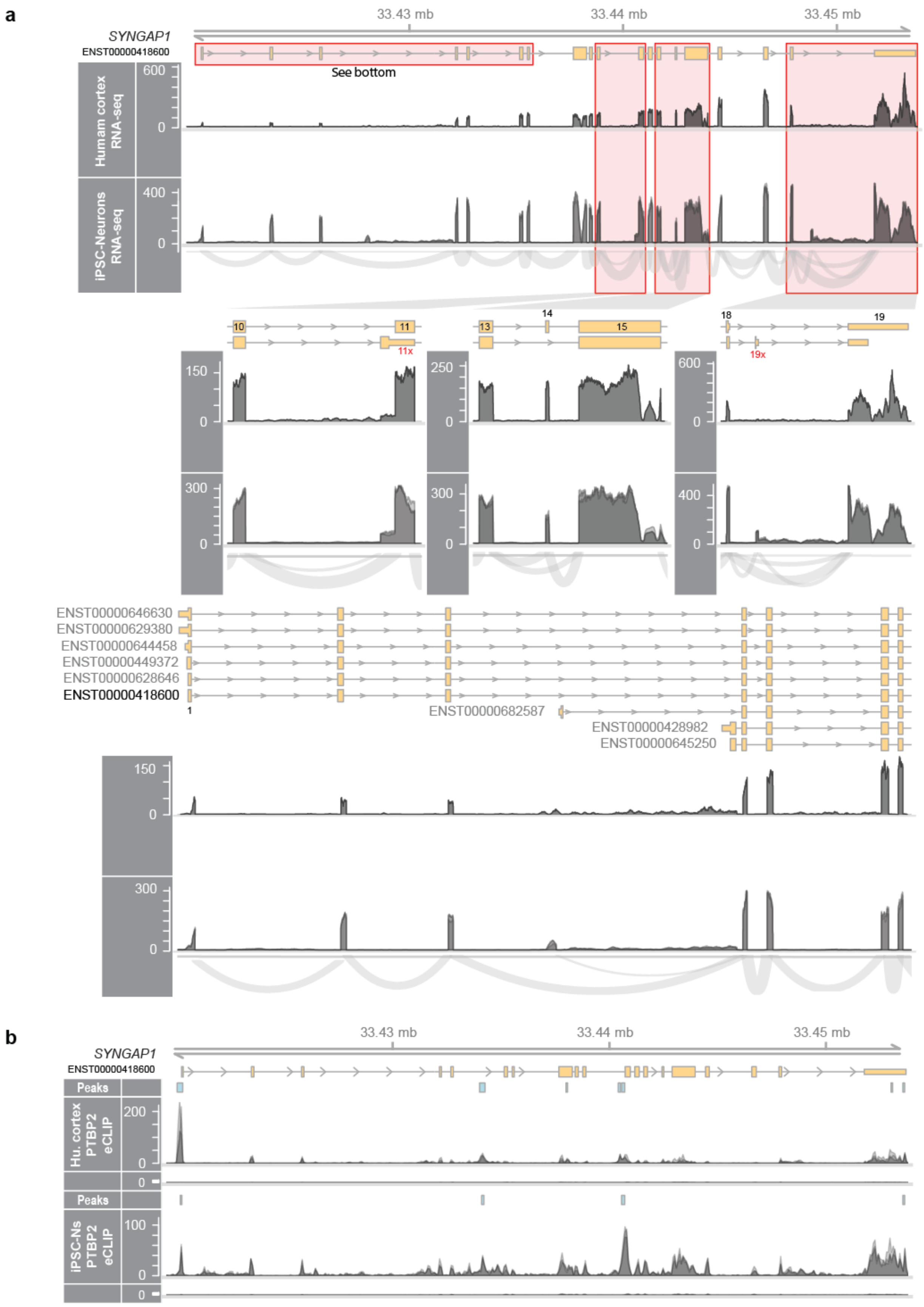

a
33.43 mb
33.44 mb
33.45 mb
SYNGAP1
ENST00000418600
See bottom
Humam cortex RNA-seq
iPSC-Neurons RNA-seq
600
0
400
0
10
11
11x
13
14
15
18
19
19x
150
0
250
0
600
0
300
0
300
0
400
0
ENST00000646630
ENST00000629380
ENST00000644458
ENST00000449372
ENST00000628646
ENST00000418600
1
ENST00000682587
ENST00000428982
ENST00000645250
150
0
300
0
b
33.43 mb
33.44 mb
33.45 mb
SYNGAP1
ENST00000418600
Peaks
Hu. cortex PTBP2 eCLIP
200
0
0
Peaks
iPSC-Ns PTBP2 eCLIP
100
0
0

Supplementary Figure 3. PTBP2 binding and alternative splicing of SYNGAP1. **(a)** (Top) Gene model of SYNGAP1 followed by RNA-seq read coverage for human cortex and RNA-seq read coverage and sashimi plots for untreated control iPSC-neurons. (Insets) Zoom-ins for regions of interest. The human cortex RNA-seq represents 101 samples from GTEx Brain Front Cortex (BA9). *n* = 3 replicates (overlaid) for iPSC-neurons. **(b)** Gene model of ENST00000418600, the dominant SYNGAP1 isoform in brain, followed by human cortex CLIP-seq (peaks, PTBP2 eCLIP read coverage, size-matched input read coverage), then iPSC-neurons CLIP-seq (as for cortex). *n* = 3 replicates (overlaid) for PTBP2 CLIP-seq and size-matched input controls. Source data are provided as a Source Data file.

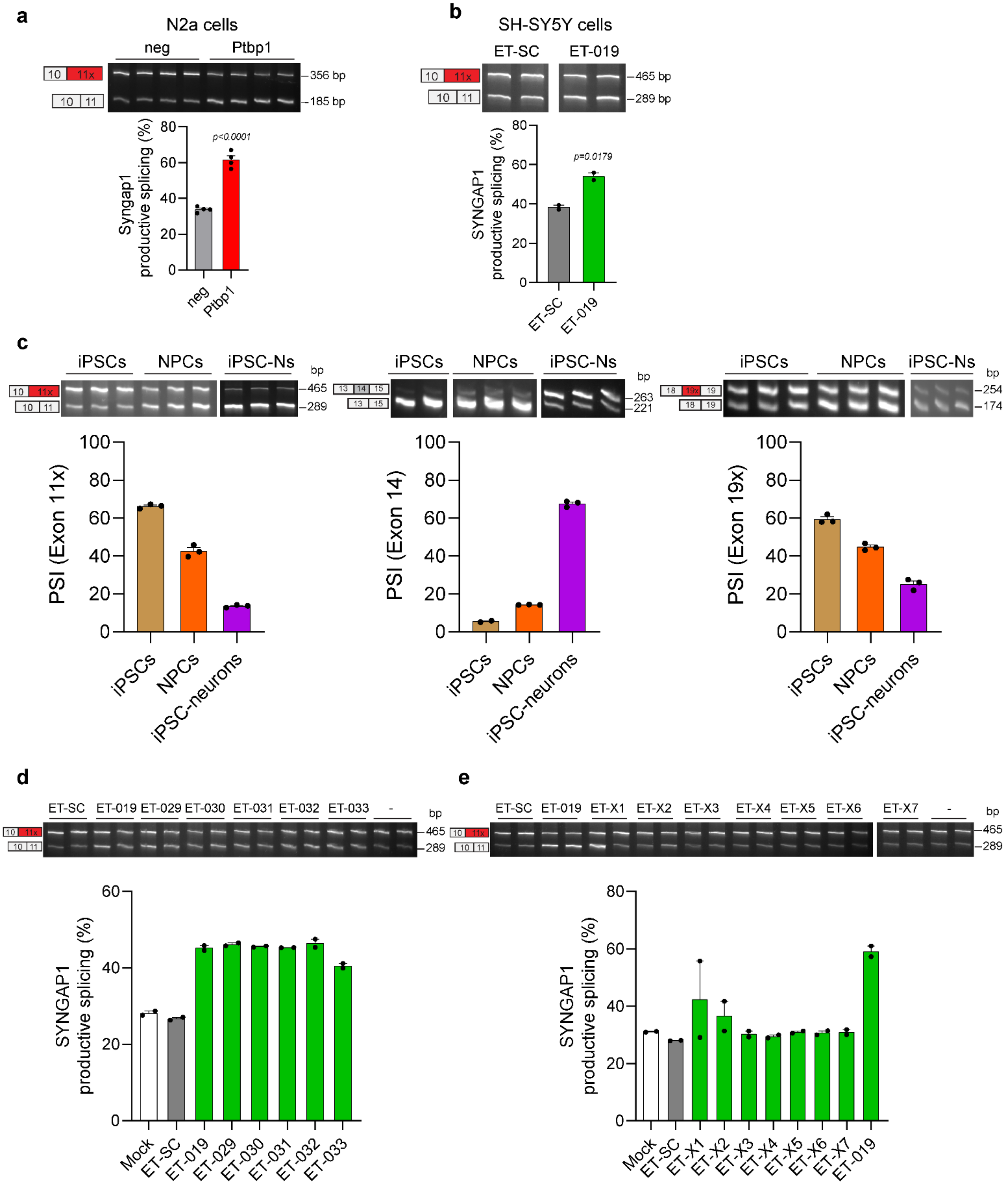

a
N2a cells
neg
Ptbp1
356 bp
185 bp
Syngap1 productive splicing (%)
p<0.0001
neg
Ptbp1
b
SH-SY5Y cells
ET-SC
ET-019
465 bp
289 bp
SYNGAP1 productive splicing (%)
p=0.0179
ET-SC
ET-019
c
iPSCs
NPCs
iPSC-Ns
bp
465
289
263
221
254
174
PSI (Exon 11x)
PSI (Exon 14)
PSI (Exon 19x)
iPSCs
NPCs
iPSC-neurons
d
ET-SC
ET-019
ET-029
ET-030
ET-031
ET-032
ET-033
-
bp
465
289
SYNGAP1 productive splicing (%)
Mock
e
ET-X1
ET-X2
ET-X3
ET-X4
ET-X5
ET-X6
ET-X7

Supplementary Figure 4. Disrupting PTBP binding in SYNGAP1 site 1 improves SYNGAP1 productive splicing. (**a**) RT-PCR from N2a cells transfected with either *Ptbp1* gapmer or negative control (neg). (**b**) RT-PCR from SH-SY5Y cells electroporated with 20 µM of ET-019 or negative control ASO (ET-SC) for 24 h. (**c**) Quantification (RT-PCR) of changes in SYNGAP1 AS on (left) exon 11x, (middle) exon 14 and (right) exon 19x in iPSCs, NPCs and iPSC-neurons. (**d**) and (**e**) RT-PCR from HEK293T cells transfected for 24h with 100 nM of ASO targeting SYNGAP1 site 1, including a positive ASO control (ET-019), a non-targeting ASO control (ET-SC) and no ASO control (Mock, -). Data are represented as mean values ± SEM. All data points represent independent biological replicates. **a** (*n* = 4). **b**, **d**, **e** (*n* = 2). **c** (*n* = 3, except *n* = 2 for iPSCs in middle panel). In **a** and **b,** Student's t-test. Source data are provided as a Source Data file.

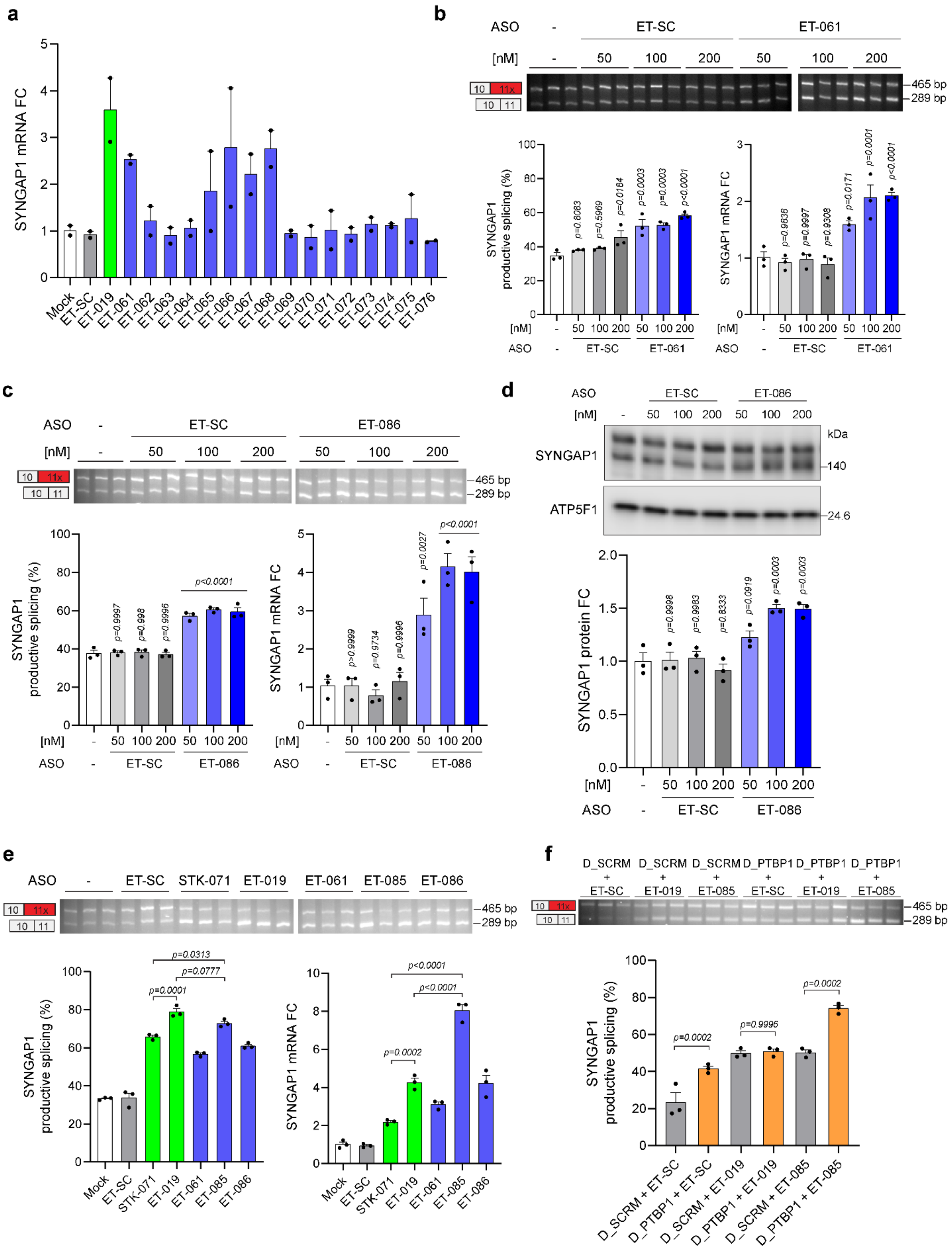

a
SYNGAP1 mRNA FC
0 1 2 3 4 5
Mock ET-SC ET-019 ET-061 ET-062 ET-063 ET-064 ET-065 ET-066 ET-067 ET-068 ET-069 ET-070 ET-071 ET-072 ET-073 ET-074 ET-075 ET-076
b
ASO - ET-SC ET-061
[nM] - 50 100 200 50 100 200
10 11x
10 11
465 bp
289 bp
SYNGAP1 productive splicing (%)
p=0.8083 p=0.5969 p=0.0184 p=0.0003 p=0.0003 p<0.0001
SYNGAP1 mRNA FC
p=0.9838 p=0.9997 p=0.9308 p=0.0171 p=0.0001 p<0.0001
[nM] - 50 100 200 50 100 200
ASO - ET-SC ET-061
c
ASO - ET-SC ET-086
[nM] - 50 100 200 50 100 200
10 11x
10 11
465 bp
289 bp
SYNGAP1 productive splicing (%)
p=0.9997 p=0.998 p=0.9996 p<0.0001
SYNGAP1 mRNA FC
p>0.9999 p=0.9734 p=0.9996 p=0.0027 p<0.0001
[nM] - 50 100 200 50 100 200
ASO - ET-SC ET-086
d
ASO ET-SC ET-086
[nM] - 50 100 200 50 100 200
kDa
SYNGAP1
140
ATP5F1
24.6
SYNGAP1 protein FC
p=0.9998 p=0.9983 p=0.8333 p=0.0919 p=0.0003 p=0.0003
[nM] - 50 100 200 50 100 200
ASO - ET-SC ET-086
e
ASO - ET-SC STK-071 ET-019 ET-061 ET-085 ET-086
10 11x
10 11
465 bp
289 bp
SYNGAP1 productive splicing (%)
p=0.0313 p=0.0777 p=0.0001
SYNGAP1 mRNA FC
p<0.0001 p<0.0001 p=0.0002
Mock ET-SC STK-071 ET-019 ET-061 ET-085 ET-086
f
D_SCRM + ET-SC
D_SCRM + ET-019
D_SCRM + ET-085
D_PTBP1 + ET-SC
D_PTBP1 + ET-019
D_PTBP1 + ET-085
10 11x
10 11
465 bp
289 bp
SYNGAP1 productive splicing (%)
p=0.0002 p=0.9996 p=0.0002
D_SCRM + ET-SC D_PTBP1 + ET-SC D_SCRM + ET-019 D_PTBP1 + ET-019 D_SCRM + ET-085 D_PTBP1 + ET-085

Supplementary Figure 5. Disrupting PTBP binding in *SYNGAP1* exon 11x upregulates SYNGAP1. (**a**) qPCR showing SYNGAP1 mRNA levels from samples in Fig. 6B. (**b**) and (**c**) Top and left panels: RT-PCR from HEK293T cells transfected with increasing concentrations of ET-061 (**b**) or ET-086 (**c**) for 48 h, including matching concentrations of the non-targeting ASO control (ET-SC) and no ASO control (Mock, -). Right panels: qPCR showing SYNGAP1 mRNA levels. (**d**) Western blot from HEK293T cells transfected as in (c). (**e**) Top and left panel: RT-PCR from HEK293T cells transfected with 100 nM of ASO for 48 h, including matching concentrations of the non-targeting ASO control (ET-SC) and no ASO control (Mock). Right panel: qPCR showing SYNGAP1 mRNA levels. (**f**) RT-PCR from HEK293T cells transfected with 5 µM of decoy oligo for 24 h followed by ASO transfection (200 nM) for an additional 24 h. D_SCRM and ET-SC oligos were included as negative controls. Data are represented as mean values ± SEM. All data points represent independent biological replicates. **a** (n = 2). **b**-**f** (n = 3). In **b**-**d**, one-way ANOVA with Dunnett's multiple comparison test vs mock-treated cells (-). In **e-f**, one-way ANOVA with Tukey's multiple comparison test. Source data are provided as a Source Data file. FC, fold change.

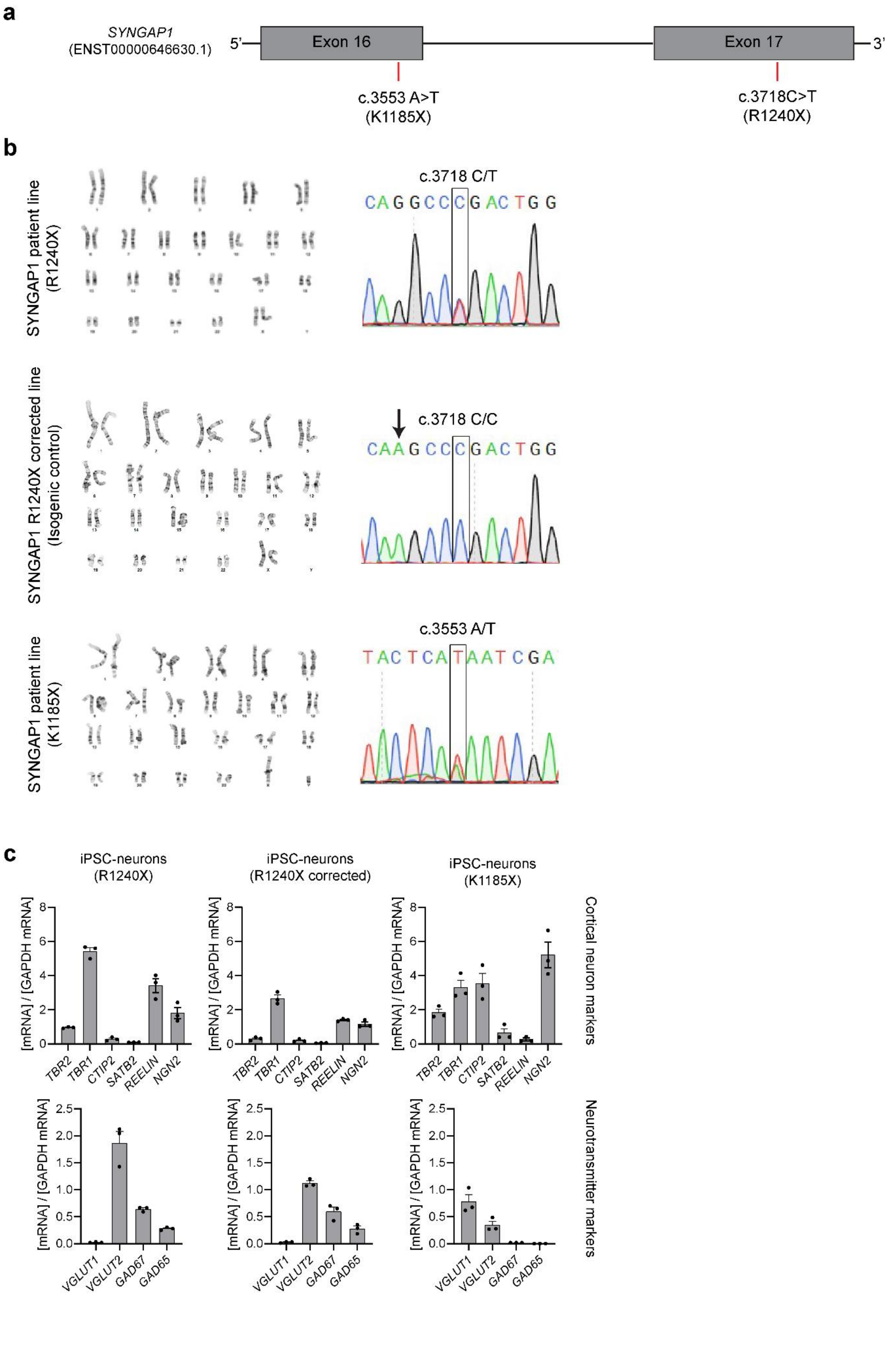

a
SYNGAP1
(ENST00000646630.1)
5'
Exon 16
Exon 17
3'
c.3553 A>T
(K1185X)
c.3718C>T
(R1240X)
b
SYNGAP1 patient line (R1240X)
c.3718 C/T
CAGGCCCGACTGG
SYNGAP1 R1240X corrected line (Isogenic control)
c.3718 C/C
CAAGCCCGACTGG
SYNGAP1 patient line (K1185X)
c.3553 A/T
TACTCATAATCGA
c
iPSC-neurons (R1240X)
iPSC-neurons (R1240X corrected)
iPSC-neurons (K1185X)
[mRNA] / [GAPDH mRNA]
TBR2
TBR1
CTIP2
SATB2
REELIN
NGN2
Cortical neuron markers
VGLUT1
VGLUT2
GAD67
GAD65
Neurotransmitter markers

Supplementary Figure 6. Generation and characterization of SYNGAP1 patient and corrected iPSC lines and iPSC-neurons. (**a**) Schematic of *SYNGAP1* mRNA showing the location of the heterozygous mutations present in the two independent SYNGAP1 patient iPSC lines. (**b**) Left, karyotype analyses. Right, chromatogram from genomic DNA sequencing to confirm the presence of the corresponding heterozygous mutation in SYNGAP1 patient iPSC lines or the corrected nucleotide in the R1240X corrected line (isogenic control). Black arrow indicates a silent mutation introduced in the R1240X corrected line by CRISPR/Cas9 to prevent re-cleavage of the recombined single-stranded oligonucleotide. (**c**) Characterization of SYNGAP1 patient lines and isogenic control after directed differentiation into cortical excitatory neurons. Top, qPCR of SYNGAP1 patient iPSC-neurons for transcripts of the excitatory cortical cell types. Bottom, qPCR of SYNGAP1 iPSC-neurons for transcripts of excitatory markers and inhibitory markers, exhibiting predominant excitatory phenotype like that of WT iPSC-neurons. Data are represented as mean values ± SEM. All data points represent independent biological replicates. **c** (*n* = 3). Source data are provided as a Source Data file.

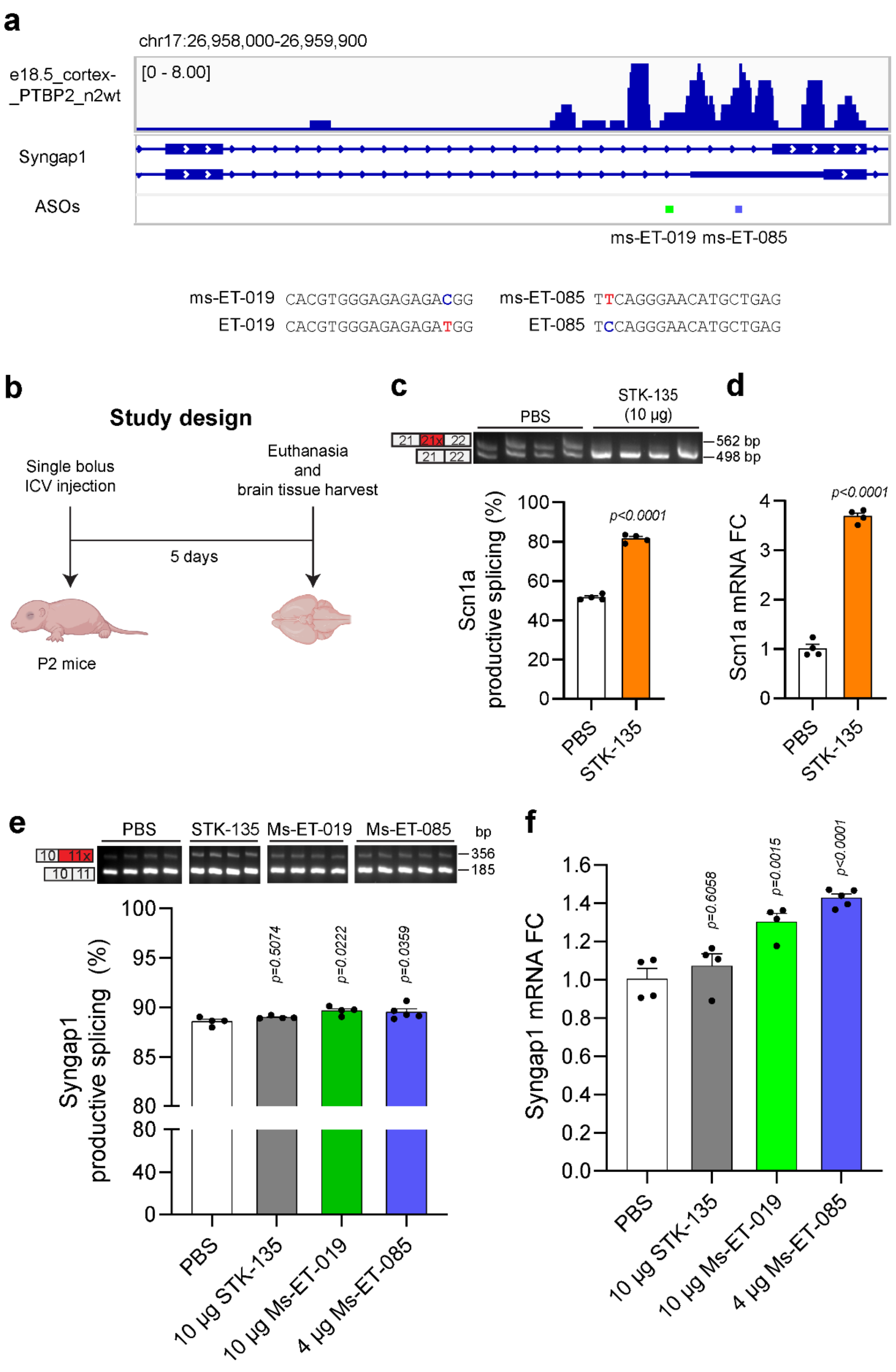

a
chr17:26,958,000-26,959,900
e18.5_cortex-_PTBP2_n2wt
[0 - 8.00]
Syngap1
ASOs
ms-ET-019 ms-ET-085
ms-ET-019 CACGTGGGAGAGAGACGG
ET-019 CACGTGGGAGAGAGATGG
ms-ET-085 TTCAGGGAACATGCTGAG
ET-085 TCCAGGGAACATGCTGAG
b
Study design
Single bolus ICV injection
Euthanasia and brain tissue harvest
5 days
P2 mice
c
PBS
STK-135 (10 µg)
21 21x 22
21 22
562 bp
498 bp
Scn1a productive splicing (%)
p<0.0001
PBS
STK-135
d
Scn1a mRNA FC
p<0.0001
PBS
STK-135
e
PBS
STK-135
Ms-ET-019
Ms-ET-085
bp
10 11x
10 11
356
185
Syngap1 productive splicing (%)
p=0.5074
p=0.0222
p=0.0359
PBS
10 µg STK-135
10 µg Ms-ET-019
4 µg Ms-ET-085
f
Syngap1 mRNA FC
p=0.6058
p=0.0015
p<0.0001
PBS
10 µg STK-135
10 µg Ms-ET-019
4 µg Ms-ET-085

Supplementary Figure 7. Intracerebroventricular injection of ASOs in neonatal mice increases Syngap1 mRNA expression. (**a**) Top, processed mouse e18.5 cortex Ptbp2 CLIP-seq data from Saito et. al.[1] (GEO accession GSE103315). Genomic coordinates are based on mm10. Middle, gene models for *Syngap1* exons 10-11 3'-AS event. Bottom, location of ASOs targeting mouse *Syngap1* and comparison of ASO sequences (5'-3') targeting mouse *Syngap1* and human *SYNGAP1* mRNAs. (**b**) Experimental design for evaluation of *Syngap1* ASOs *in vivo*. P2 mice were injected with PBS, a positive control ASO targeting the non-productive exon inclusion in *Scn1a* (STK-135, 10 µg) previously reported by Lim and coworkers[2], Ms-ET-019 (10 µg) and Ms-ET-085 (4 µg). Mice were euthanized at P7, and brain tissues were harvested and analyzed for productive exon exclusion in *Scn1a* upon STK-135 treatment, and productive exclusion of the alternative 3'ss in *Syngap1* upon *Syngap1* ASO treatments. Created with BioRender.com. (**c**) *Scn1a* RT-PCR assay from mouse brains injected with 10 µg of STK-135. The percentage of exon 21x exclusion (productive splicing) in *Scn1a* transcript was calculated based on densitometric analysis of RT-PCR products. (**d**) qPCR showing *Scn1a* productive transcript levels. (**e**) RT-PCR assay of *Syngap1* Ex11 productive splicing. (**f**) qPCR showing *Syngap1* productive transcript levels. Data are represented as mean values ± SEM. All data points represent independent biological replicates. **c**, **d** (n = 4). **e**, **f** (n = 4 except n = 5 for Ms-ET-085). In **c** and **d**, Student's t-test. In **e** and **f**, one-way ANOVA with Dunnett's multiple comparison test vs PBS. Source data are provided as a Source Data file. FC, fold change.

Supplementary References:

1. Saito, Y. *et al.* Differential NOVA2-Mediated Splicing in Excitatory and Inhibitory Neurons Regulates Cortical Development and Cerebellar Function. *Neuron* **101**, 707-720.e5 (2019).

2. Lim, K. H. *et al.* Antisense oligonucleotide modulation of non-productive alternative splicing upregulates gene expression. *Nature Communications 2020 11:1* **11**, 1–13 (2020).
