# Supplementary material for: Mapping PTBP2 binding in human brain identifies SYNGAP1 as a target for therapeutic splice switching
Source: Nat Commun. 2023 May 6;14:2628. doi: 10.1038/s41467-023-38273-3 (PMC10164156; doi:10.1038/s41467-023-38273-3)
Supplement: Supplementary file 2 — Description of Additional Supplementary Files [file 41467_2023_38273_MOESM2_ESM.pdf]

Description of Additional Supplementary Files

File Name: Supplementary Data 1
Description: Differential gene expression analysis in iPSC-neurons following PTBP2 KD.

File Name: Supplementary Data 2
Description: Table of differentially spliced genes in iPSC-neurons (PTBP2 KD vs. Untreated) along with Orphanet status, up-regulation/down-regulation at the gene level, and CLIP-seq peak calls proximal to splice event.

File Name: Supplementary Data 3
Description: Differential splicing of Orphanet genes by alternative splicing event.

File Name: Supplementary Data 4
Description: PTBP2 peak calls for human cortex.

File Name: Supplementary Data 5
Description: PTBP2 peak calls for iPSC-neurons.

File Name: Supplementary Data 6
Description: Sequence and chemistry of oligonucleotides used in study.

File Name: Supplementary Data 7
Description: Primer sequences used for assessing expression of neuronal markers.

File Name: Supplementary Movie 1
Description: iPSC-neurons loaded with a calcium indicator dye (Fluo-4 AM) at rest and electrically stimulated with 20 Hz trains of depolarizing field stimuli lasting 10 s, with 20 s of rest between trains.
