# Supplementary material for: Mapping PTBP2 binding in human brain identifies SYNGAP1 as a target for therapeutic splice switching
Source: Nat Commun. 2023 May 6;14:2628. doi: 10.1038/s41467-023-38273-3 (PMC10164156; doi:10.1038/s41467-023-38273-3)
Supplement: Supplementary file 11 — Reporting Summary [file 41467_2023_38273_MOESM11_ESM.pdf]

nature portfolio

Corresponding author(s): Benjamin L. Prosser, Beverly L. Davidson

Last updated by author(s): Mar 28, 2023

Reporting Summary

Nature Portfolio wishes to improve the reproducibility of the work that we publish. This form provides structure for consistency and transparency in reporting. For further information on Nature Portfolio policies, see our Editorial Policies and the Editorial Policy Checklist.

Statistics

For all statistical analyses, confirm that the following items are present in the figure legend, table legend, main text, or Methods section.

n/a	Confirmed	
☐	☒	The exact sample size (*n*) for each experimental group/condition, given as a discrete number and unit of measurement
☐	☒	A statement on whether measurements were taken from distinct samples or whether the same sample was measured repeatedly
☐	☒	The statistical test(s) used AND whether they are one- or two-sided<br>*Only common tests should be described solely by name; describe more complex techniques in the Methods section.*
☒	☐	A description of all covariates tested
☐	☒	A description of any assumptions or corrections, such as tests of normality and adjustment for multiple comparisons
☐	☒	A full description of the statistical parameters including central tendency (e.g. means) or other basic estimates (e.g. regression coefficient) AND variation (e.g. standard deviation) or associated estimates of uncertainty (e.g. confidence intervals)
☐	☒	For null hypothesis testing, the test statistic (e.g. *F*, *t*, *r*) with confidence intervals, effect sizes, degrees of freedom and *P* value noted<br>*Give P values as exact values whenever suitable.*
☒	☐	For Bayesian analysis, information on the choice of priors and Markov chain Monte Carlo settings
☒	☐	For hierarchical and complex designs, identification of the appropriate level for tests and full reporting of outcomes
☒	☐	Estimates of effect sizes (e.g. Cohen's *d*, Pearson's *r*), indicating how they were calculated

Our web collection on statistics for biologists contains articles on many of the points above.

Software and code

Policy information about availability of computer code

Data collection	Image collection and Airyscan processing was performed using Zen 2.1 SP3 FP2 black software (Zeiss). Alternatively, images were taken on a Leica DMi4000 inverted microscope. LICOR Image Studio Ver5.2 or GBox imaging system (GeneSys Version 1.5.6.0) was used to image western blots. QuantStudio 3 or QuantStudio 5 was used to collect qPCR amplification data using QuantStudio Design and Analysis Software v1.4.1 or v1.5.2, respectively. Sequencing data was collected on a NovaSeq 6000.
Data analysis	(Fiji Is Just) ImageJ 2.3.0/1.53f was used for preparation of microscopy images. Image Studio Lite ver5.2 was used for western blot quantification. GraphPad Prism 9.3 software or R 4.2.1 was used for graphing and statistical analysis. Salmon v1.5.2 was used for genome mapping. DESeq2 was used for differential gene expression. STAR v2.7.1a was used for genome alignment. rMATS v4.1.1 was used for differential splicing analysis. CLAM v1.2 was used for eCLIP peak calling. RBPmaps v0.1.4 was used to create splicing regulatory maps. Adapter sequences were removed with Cutadapt v1.18. Sequence quality was assessed by FastQC v0.11.2. FlowJo 10.8.2 software was used for flow cytometry analysis.

For manuscripts utilizing custom algorithms or software that are central to the research but not yet described in published literature, software must be made available to editors and reviewers. We strongly encourage code deposition in a community repository (e.g. GitHub). See the Nature Portfolio guidelines for submitting code & software for further information.

Data

Policy information about availability of data

All manuscripts must include a data availability statement. This statement should provide the following information, where applicable:

- Accession codes, unique identifiers, or web links for publicly available datasets
- A description of any restrictions on data availability
- For clinical datasets or third party data, please ensure that the statement adheres to our policy

Data availability - The datasets generated during the current study are available in the GEO repository with accession codes GSE206661, GSE206650, and GSE206660. Raw images from DNA agarose gels and Western blots, along with source data for figures and supplemental figures, are provided as a Source Data File. Additional data that supports the findings of this study can be found in Supplementary Data Files.

Publicly available data sets - Brain Front Cortex (gtexCovBrainFrontalCortexBA9) table was obtained from the GTEx RNA-seq Coverage track on the UCSC genome browser on 5/6/2022. A list of genes associated with rare diseases were downloaded from the Orphadata repository of Orphanet (http://www.orphadata.org).

Human research participants

Policy information about studies involving human research participants and Sex and Gender in Research.

Reporting on sex and gender	Post-mortem brain tissue were from male donors. Considerations of sex were based on availability of source material.
Population characteristics	Post-mortem brain tissue were from donors aged 42 - 64 yo and included white and bi-racial donors. Considerations of population were based on availability of source material.
Recruitment	Post-mortem.
Ethics oversight	Samples were acquired from a brain resource center.

Note that full information on the approval of the study protocol must also be provided in the manuscript.

Field-specific reporting

Please select the one below that is the best fit for your research. If you are not sure, read the appropriate sections before making your selection.

☒ Life sciences ☐ Behavioural & social sciences ☐ Ecological, evolutionary & environmental sciences

For a reference copy of the document with all sections, see nature.com/documents/nr-reporting-summary-flat.pdf

Life sciences study design

All studies must disclose on these points even when the disclosure is negative.

Sample size	Sample size was determined based on author's previous experience as well as similar experiments in the published literature. We decided on the number of replicates based on the variability inherent in the experiment. The number of replicates for each experiment is provided in the manuscript. For in vivo mouse experiments, a minimum of four replicates or more depending on litter size were used.
Data exclusions	For in vivo mouse experiments, ASO doses that proved toxic were excluded from consideration.
Replication	All experiments were reproduced, typically in triplicate, and the number of biological replicates for each experiment are noted.
Randomization	Mice were randomly allocated to experimental groups. Allocation to experimental groups is not relevant for experiments not involving mice.
Blinding	Unbiased analytical approaches were used to limit investigator bias.

Reporting for specific materials, systems and methods

We require information from authors about some types of materials, experimental systems and methods used in many studies. Here, indicate whether each material, system or method listed is relevant to your study. If you are not sure if a list item applies to your research, read the appropriate section before selecting a response.

Materials & experimental systems

n/a	Involved in the study	
☐	☒	Antibodies
☐	☒	Eukaryotic cell lines
☒	☐	Palaeontology and archaeology
☐	☒	Animals and other organisms
☒	☐	Clinical data
☒	☐	Dual use research of concern

Methods

n/a	Involved in the study	
☒	☐	ChIP-seq
☒	☐	Flow cytometry
☒	☐	MRI-based neuroimaging

Antibodies

Antibodies used

Rabbit anti-PTBP1 antibody (Cell Signaling Technology #72669)
Rabbit anti-PTBP2 antibody (EMD Millipore #ABE431)
Rabbit anti-SYNGAP1 (Cell Signaling Technology #5539S)
Mouse anti-ATP5F1 (Abcam #ab117991 or Santa Cruz Biotechnology #sc-514419)
Mouse anti-GAPDH (D4C6R) (Cell Signaling Technology #97166S)
Mouse anti-MAP2 (Sigma #M1406)
Mouse anti-Tuj1 (Biolegend #801201)
Rabbit anti-PSD-95 (Cell Signaling Technology #3450)
Goat anti-IgG1-Alexa488 (Invitrogen)
Goat anti-IgG2A-Alexa647 (Invitrogen)
Goat anti-rabbit Alexa568 (Invitrogen)
Anti-rabbit-HRP (Cell Signaling Technology #7074S)
Anti-mouse-HRP (Cell Signaling Technology #7076S)
IRDye 680RD anti-rabbit (LI-COR #926-68073)
IRDye 800CW anti-mouse (LI-COR #926-32212)

Validation

- Rabbit anti-PTBP1 antibody (Cell Signaling Technology #72669):
Validated for western blotting by Cell Signaling Technology.

- Rabbit anti-PTBP2 antibody (EMD Millipore #ABE431):
Validated for western blotting and immunoprecipitation by EMD Millipore. 2 citations in www.citeab.com. Additionally validated in this manuscript by PTBP2 knock-down using PTBP2 gapmer ASO.

- Rabbit anti-SYNGAP1 (Cell Signaling Technology #5539S):
Validated for Western blotting by Cell Signaling Technology. 7 citations in www.citeab.com. Additionally validated in (Lim et al., 2020).

- Mouse anti-ATP5F1 (Abcam #ab117991):
Validated for Western blotting, Flow Cytometry and Immunofluorescence by Abcam. 11 citations in www.citeab.com.

- Mouse anti-ATP5F1 (Santa Cruz Biotechnology #sc-514419):
Validated for Western blotting and Immunofluorescence by Santa Cruz. 6 citations in www.citeab.com.

- Mouse anti-GAPDH (D4C6R) (Cell Signaling Technology #97166S):
Validated for Western blotting by Abcam. 240 citations in www.citeab.com.

- Mouse anti-MAP2 (Sigma #M1406)
Validated for Western blotting and Immunofluorescence by Sigma. 248 citations in www.citeab.com.

- Mouse anti-Tuj1 (Biolegend #801201)
Validated for Western blotting and Immunofluorescence by Biolegend. 266 citations in www.citeab.com.

- Rabbit anti-PSD-95 (Cell Signaling Technology #3450)
Validated for Western blotting and Immunofluorescence by Cell Signaling Technology. 195 citations in www.citeab.com.

Eukaryotic cell lines

Policy information about cell lines and Sex and Gender in Research

Cell line source(s)

CHOP-WT10 cell line (male) from Children's Hospital of Philadelphia.
SYNGAP1 patient R1240X (female) and K1185X (male) iPSC lines generated and described in this manuscript.
SYNGAP1 corrected R1240X (female) iPSC line generated and described in this manuscript.
HEK293T cells from ATCC
Neuro2A cells from ATCC
SH-SY5Y cells from ATCC

Authentication

CHOP-WT10 was authenticated in (Maguire et al., 2016).
SYNGAP1 patient R1240X and K1185X iPSC lines and SYNGAP1 corrected R1240X iPSC lines were authenticated by

	karyotyping, as described in this manuscript.<br>HEK293T, Neuro2a and SH-SY5Y were authenticated by ATCC.
Mycoplasma contamination	The CHOP-WT10 cell line, SYNGAP1 patient R1240X and K1185X iPSC lines, and SYNGAP1 corrected R1240X iPSC line tested negative for mycoplasma using an in-house PCR-based assay. Other cell lines were not tested for mycoplasma, but no sign of mycoplasma contamination was observed.
Commonly misidentified lines<br>(See ICLAC register)	None

Animals and other research organisms

Policy information about studies involving animals; ARRIVE guidelines recommended for reporting animal research, and Sex and Gender in Research

Laboratory animals	C57BL/6J male and female neonate mice were used. All mice were maintained on a 12:12-h light:dark cycle and mothers had ad libitum access to food and water throughout the experiments.
Wild animals	The study did not involve wild animals.
Reporting on sex	No sex-based analyses were performed.
Field-collected samples	The study did not involve field-collected samples.
Ethics oversight	Animal care and use procedures were approved and performed in accordance with the standards set forth by the Children's Hospital of Philadelphia Institutional Animal Care and Use Committee and the Guide for the Care and Use of Laboratory Animals published by the US National Institutes of Health.

Note that full information on the approval of the study protocol must also be provided in the manuscript.

March 2021
