# Supplementary figures and images for: Mapping PTBP2 binding in human brain identifies SYNGAP1 as a target for therapeutic splice switching
Source: Nat Commun. 2023 May 6;14:2628. doi: 10.1038/s41467-023-38273-3 (PMC10164156; doi:10.1038/s41467-023-38273-3)

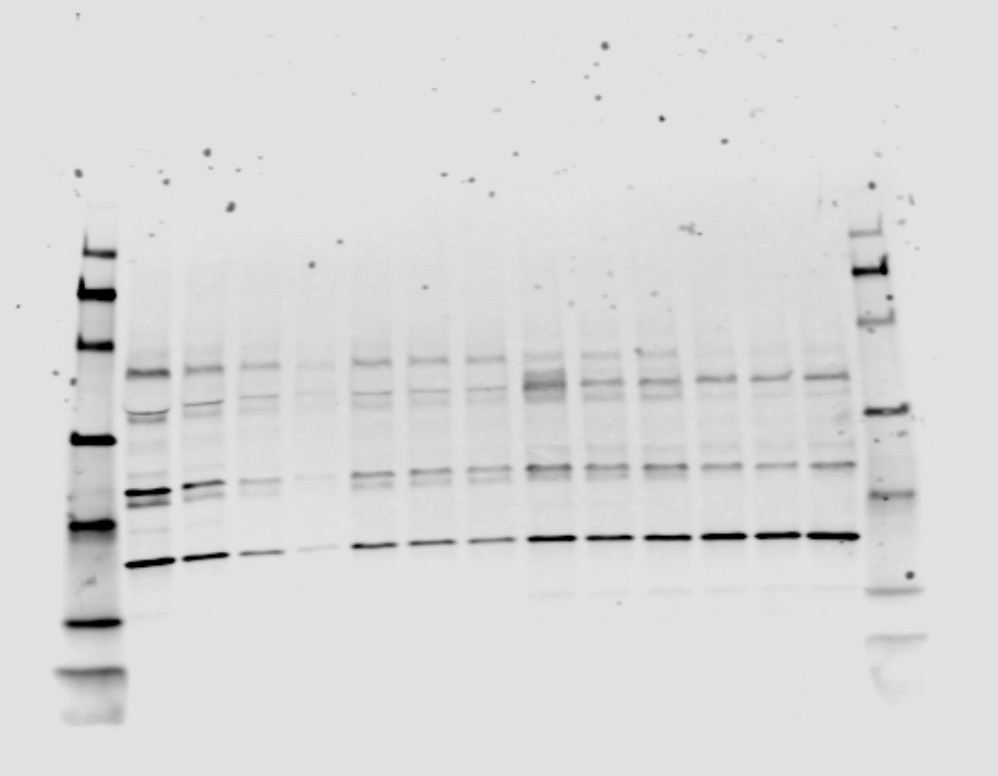

Supplement: Supplementary file 12 — Source Data [file 41467_2023_38273_MOESM12_ESM.zip › Source Data/Uncropped images/Fig1b_PTBP1blot_GAPDH_ATP5F1.tif]

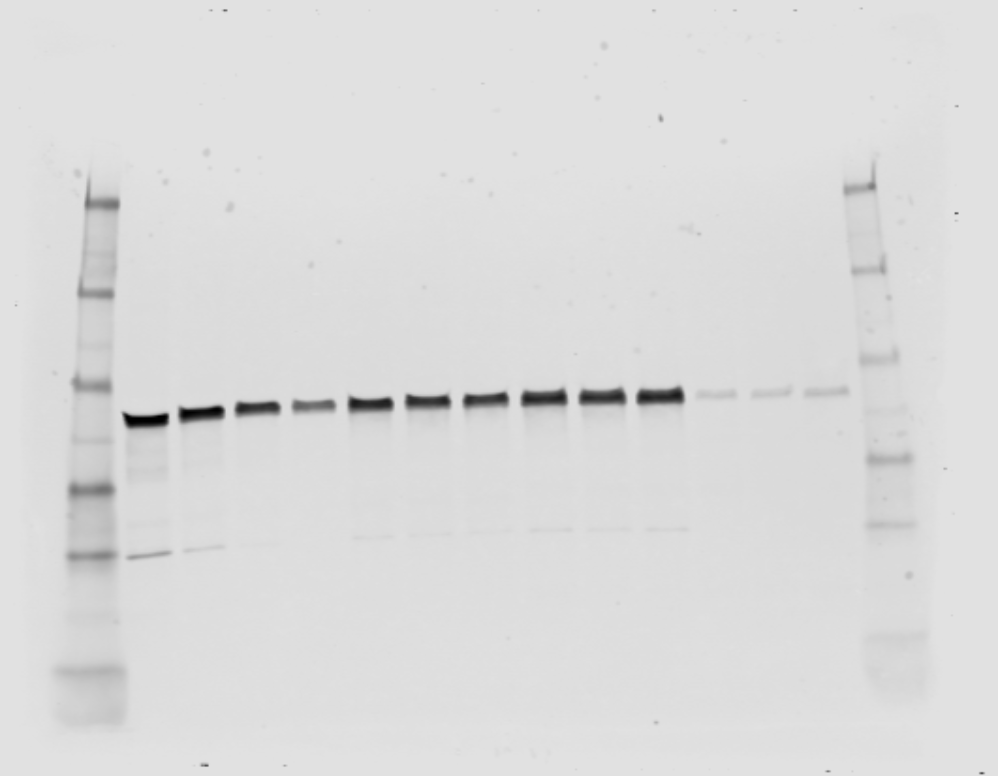

Supplement: Supplementary file 12 — Source Data [file 41467_2023_38273_MOESM12_ESM.zip › Source Data/Uncropped images/Fig1b_PTBP1blot_PTBP1.tif]

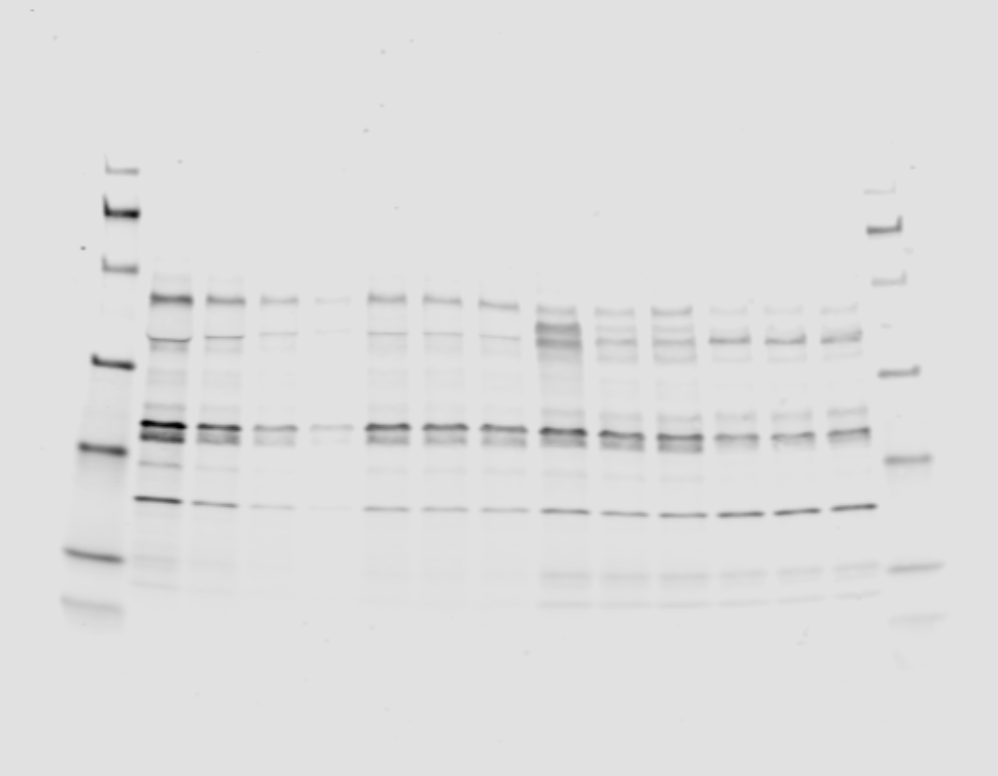

Supplement: Supplementary file 12 — Source Data [file 41467_2023_38273_MOESM12_ESM.zip › Source Data/Uncropped images/Fig1b_PTBP2blot_GAPDH_ATP5F1.tif]

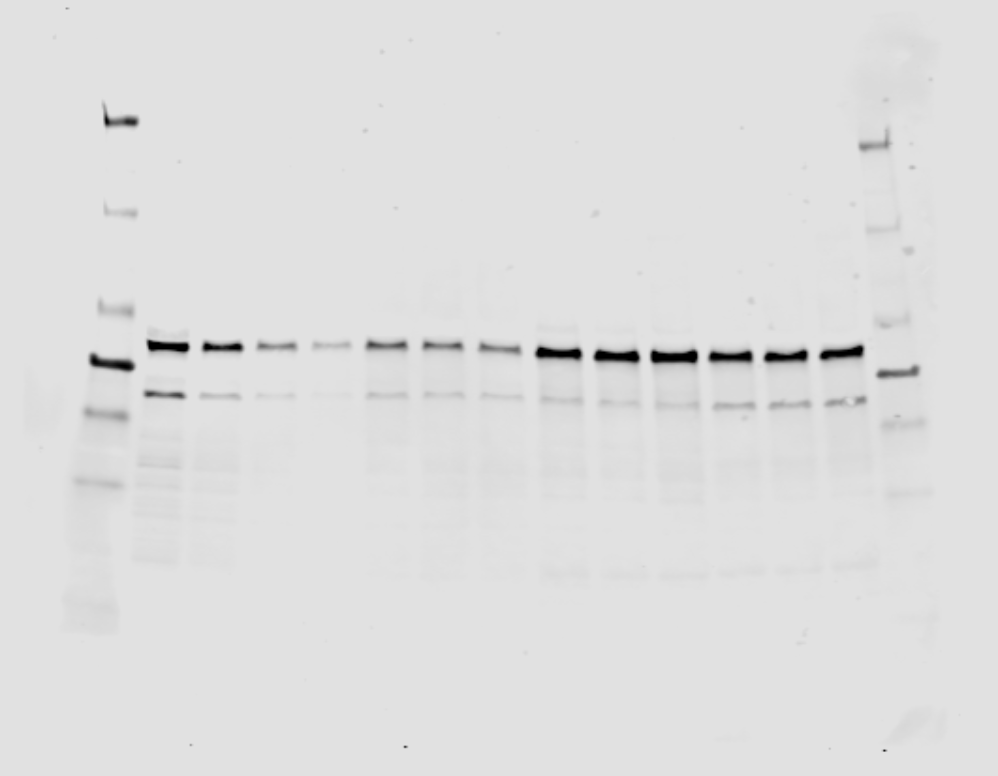

Supplement: Supplementary file 12 — Source Data [file 41467_2023_38273_MOESM12_ESM.zip › Source Data/Uncropped images/Fig1b_PTBP2blot_PTBP2.tif]

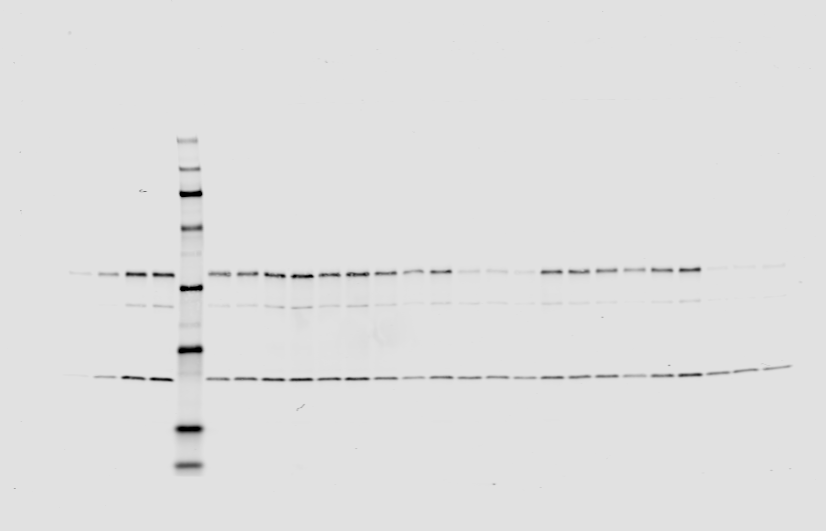

Supplement: Supplementary file 12 — Source Data [file 41467_2023_38273_MOESM12_ESM.zip › Source Data/Uncropped images/Fig1c_PTBP2blot_PTBP2_RPL4_ATP5F1.tif]

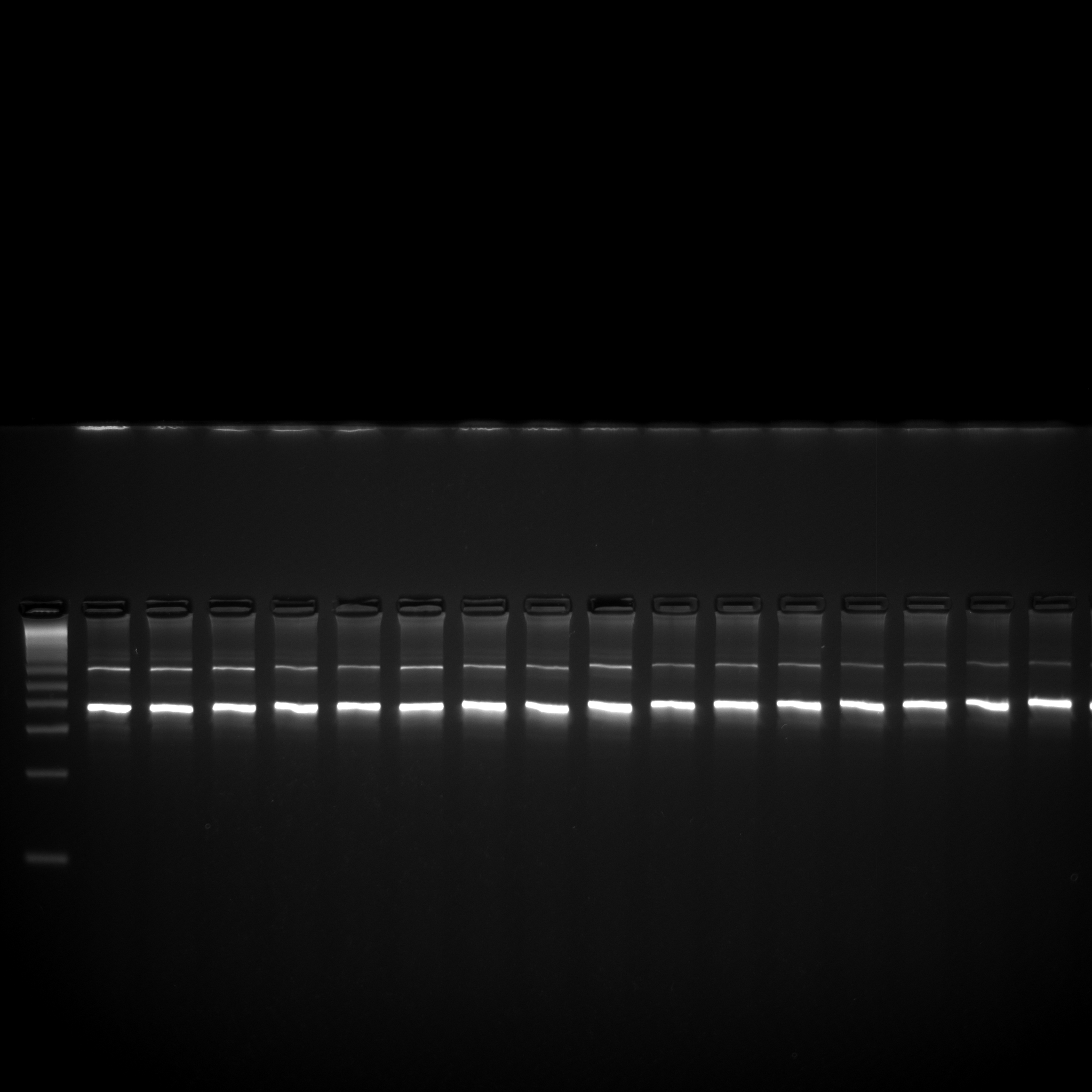

Supplement: Supplementary file 12 — Source Data [file 41467_2023_38273_MOESM12_ESM.zip › Source Data/Uncropped images/Fig4b_SYNGAP1_leftpanel_part1.tif]

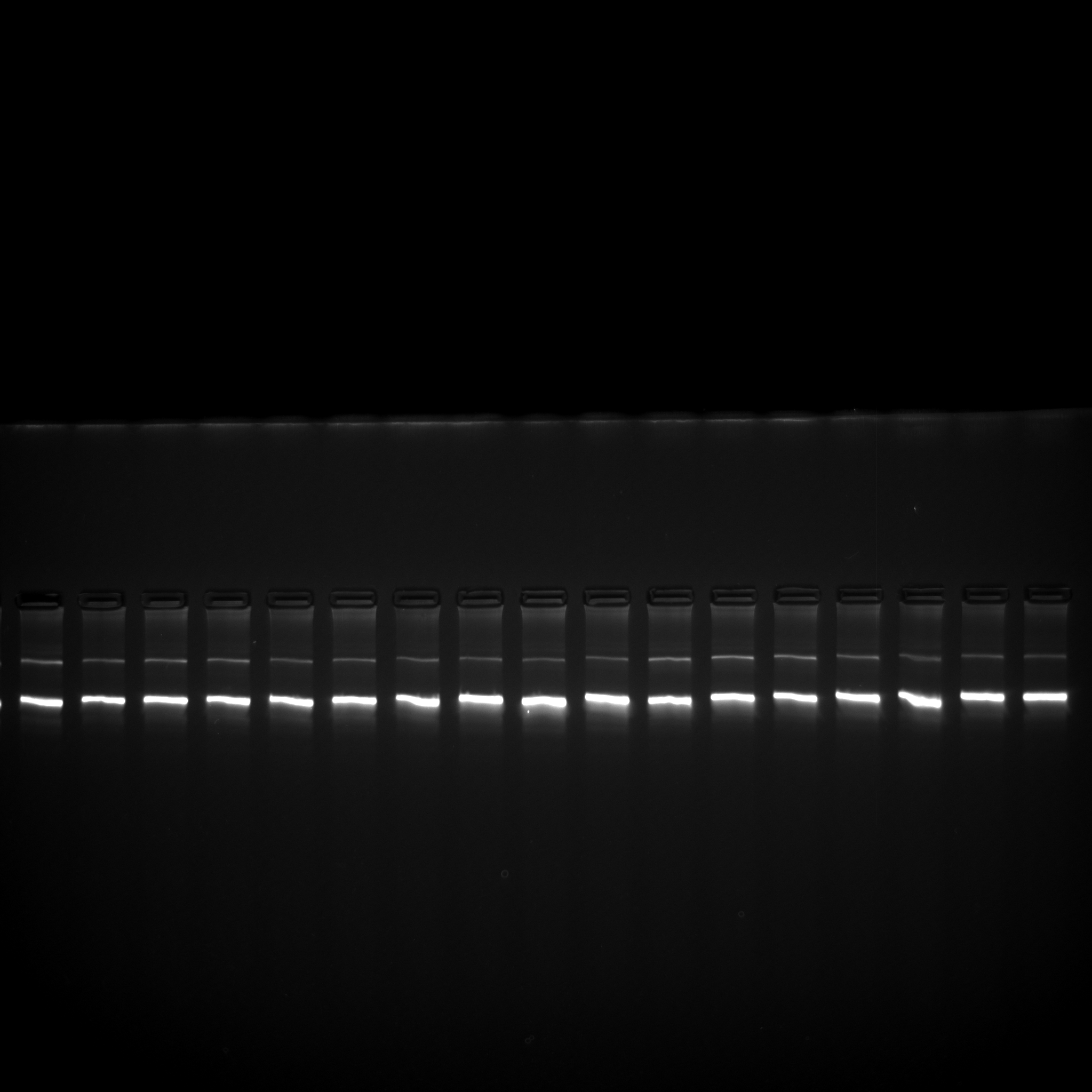

Supplement: Supplementary file 12 — Source Data [file 41467_2023_38273_MOESM12_ESM.zip › Source Data/Uncropped images/Fig4b_SYNGAP1_leftpanel_part2.tif]

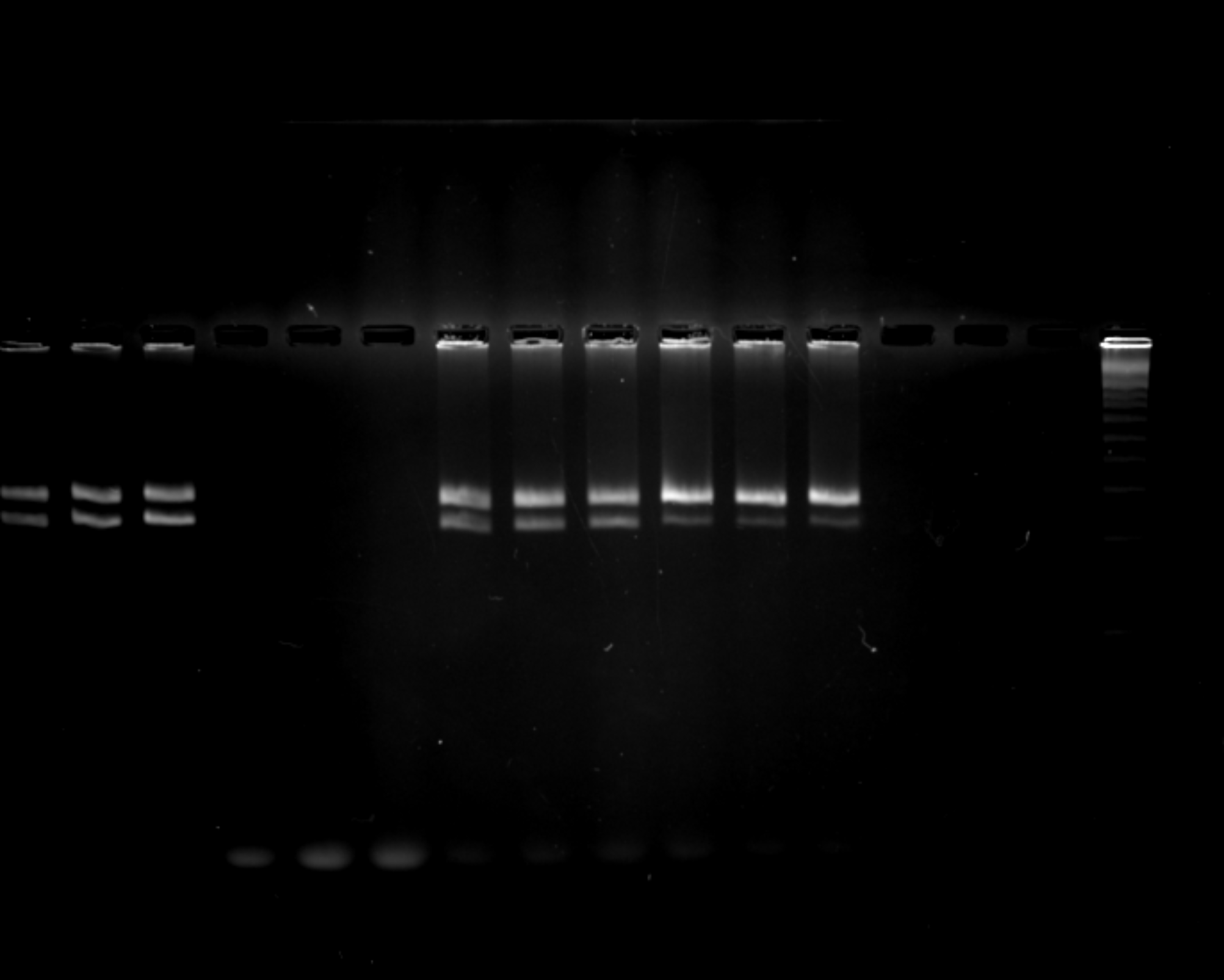

Supplement: Supplementary file 12 — Source Data [file 41467_2023_38273_MOESM12_ESM.zip › Source Data/Uncropped images/Fig4b_SYNGAP1_middlepanel.tif]

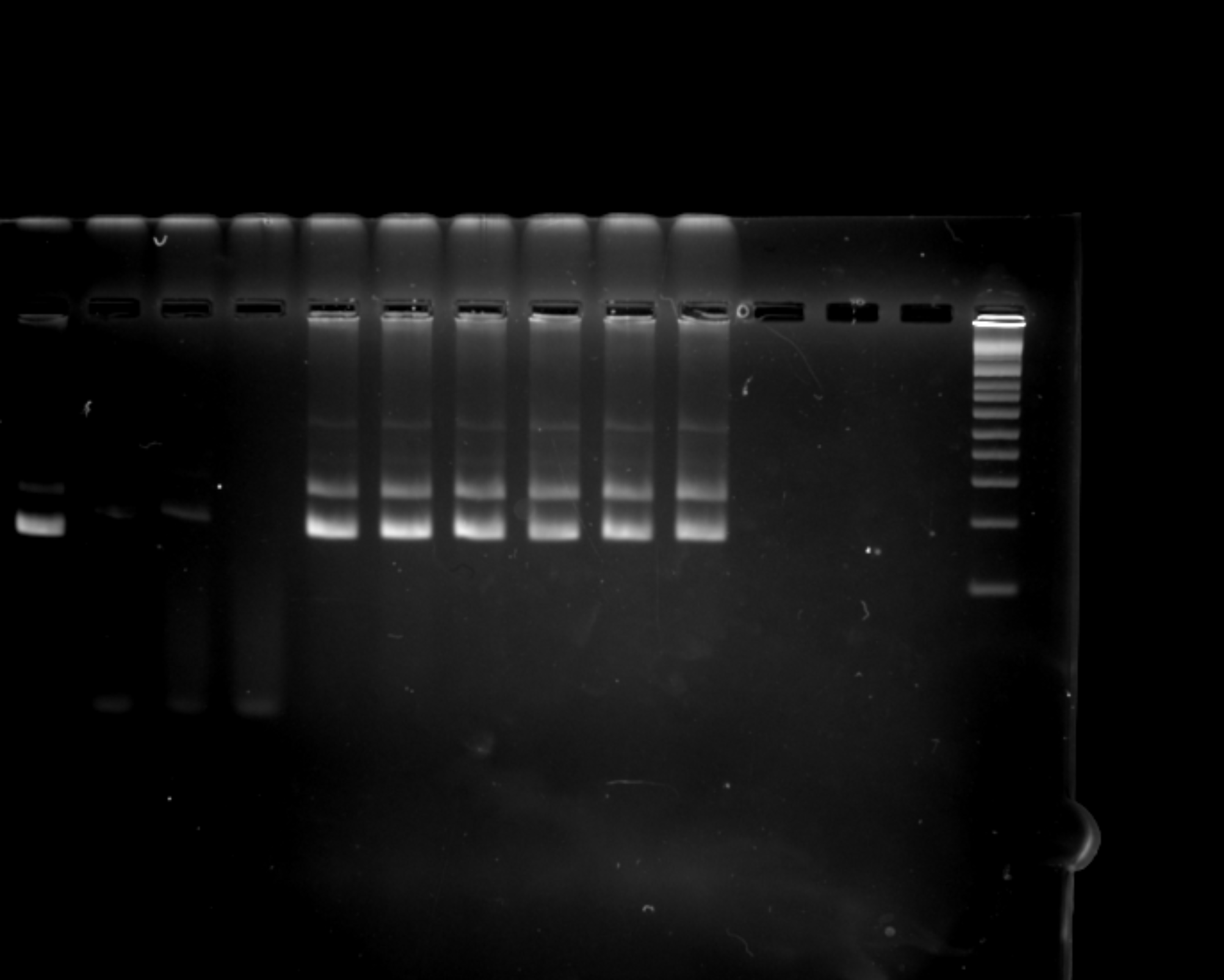

Supplement: Supplementary file 12 — Source Data [file 41467_2023_38273_MOESM12_ESM.zip › Source Data/Uncropped images/Fig4b_SYNGAP1_rightpanel.tif]

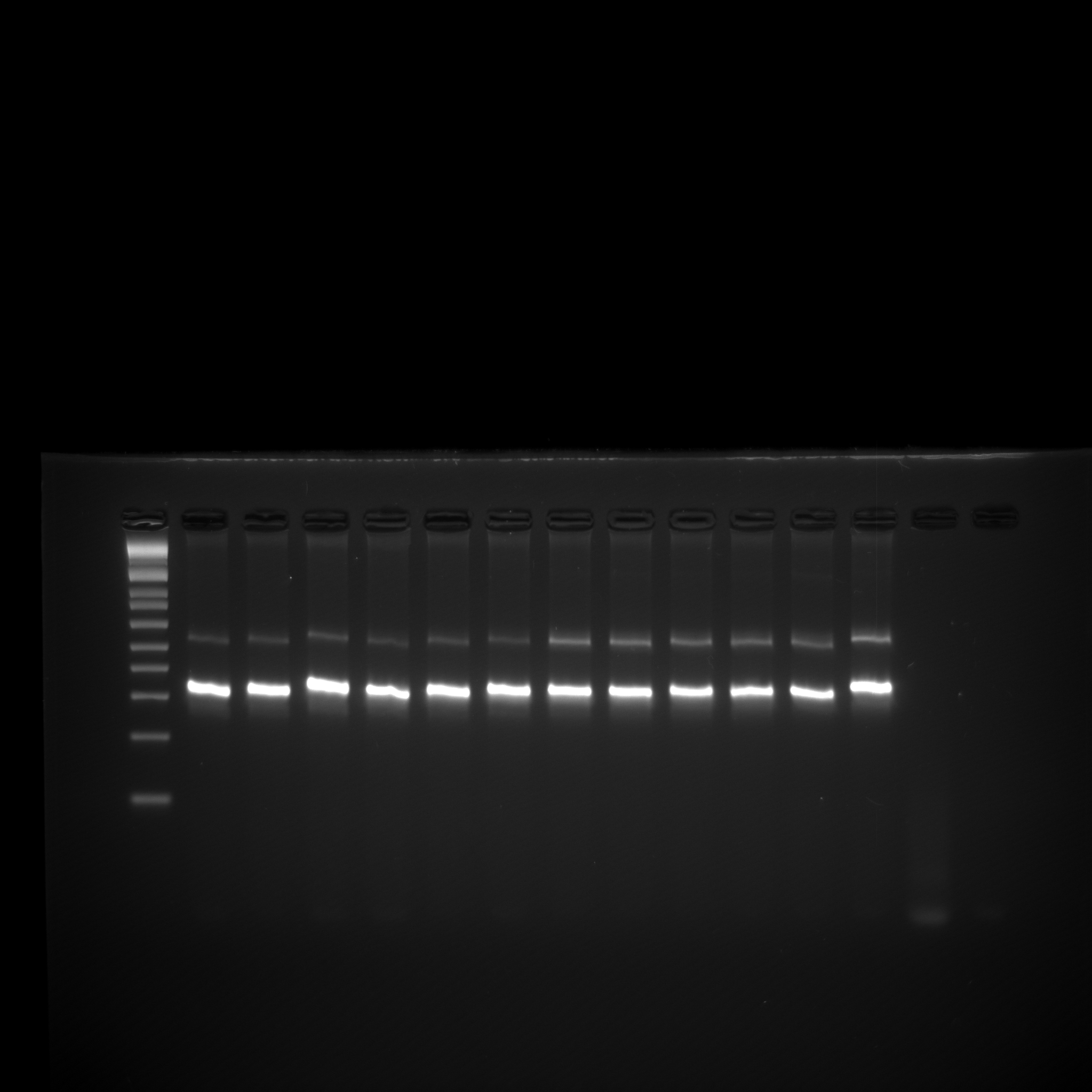

Supplement: Supplementary file 12 — Source Data [file 41467_2023_38273_MOESM12_ESM.zip › Source Data/Uncropped images/Fig4c_SYNGAP1_leftpanel.tif]

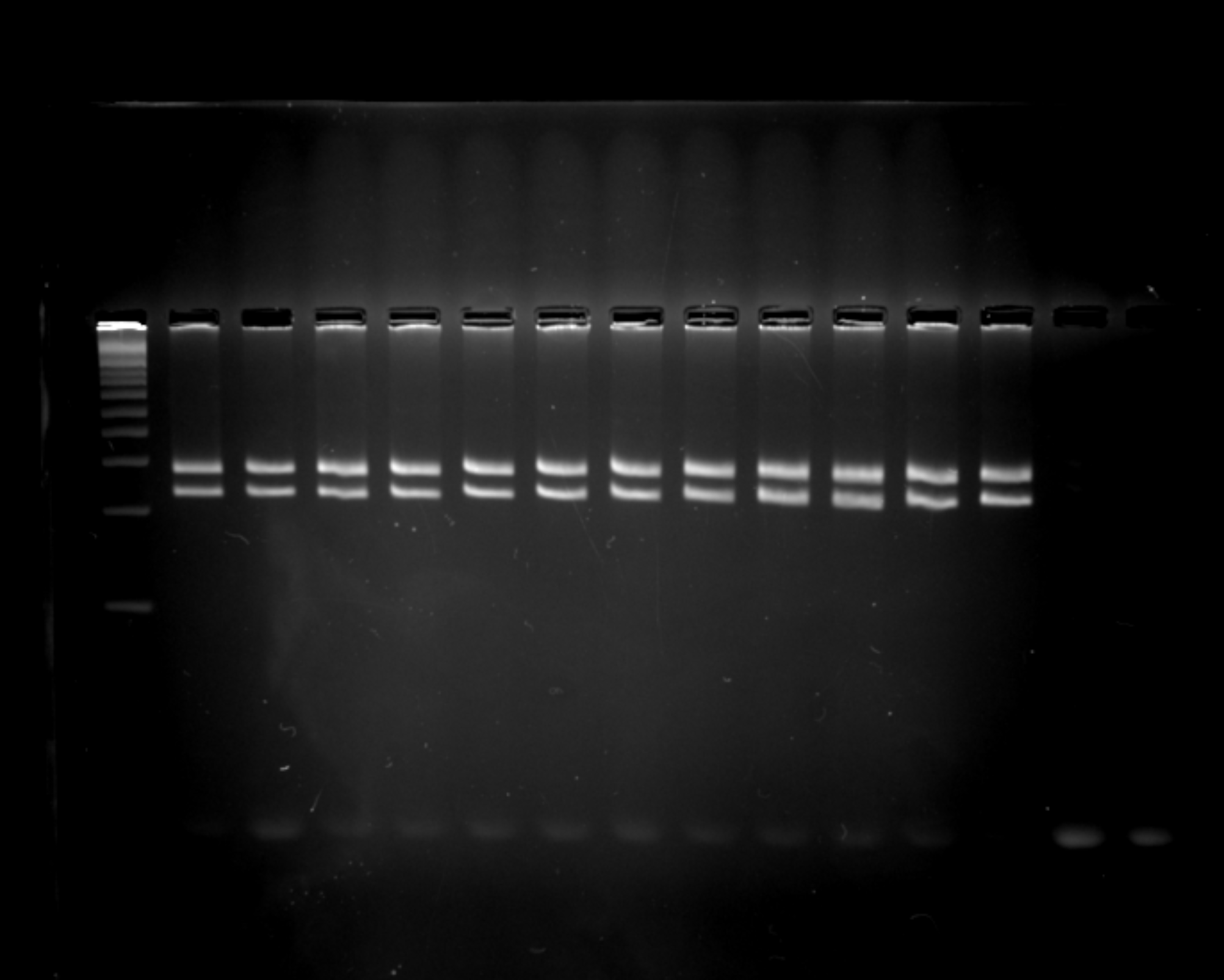

Supplement: Supplementary file 12 — Source Data [file 41467_2023_38273_MOESM12_ESM.zip › Source Data/Uncropped images/Fig4c_SYNGAP1_middlepanel.tif]

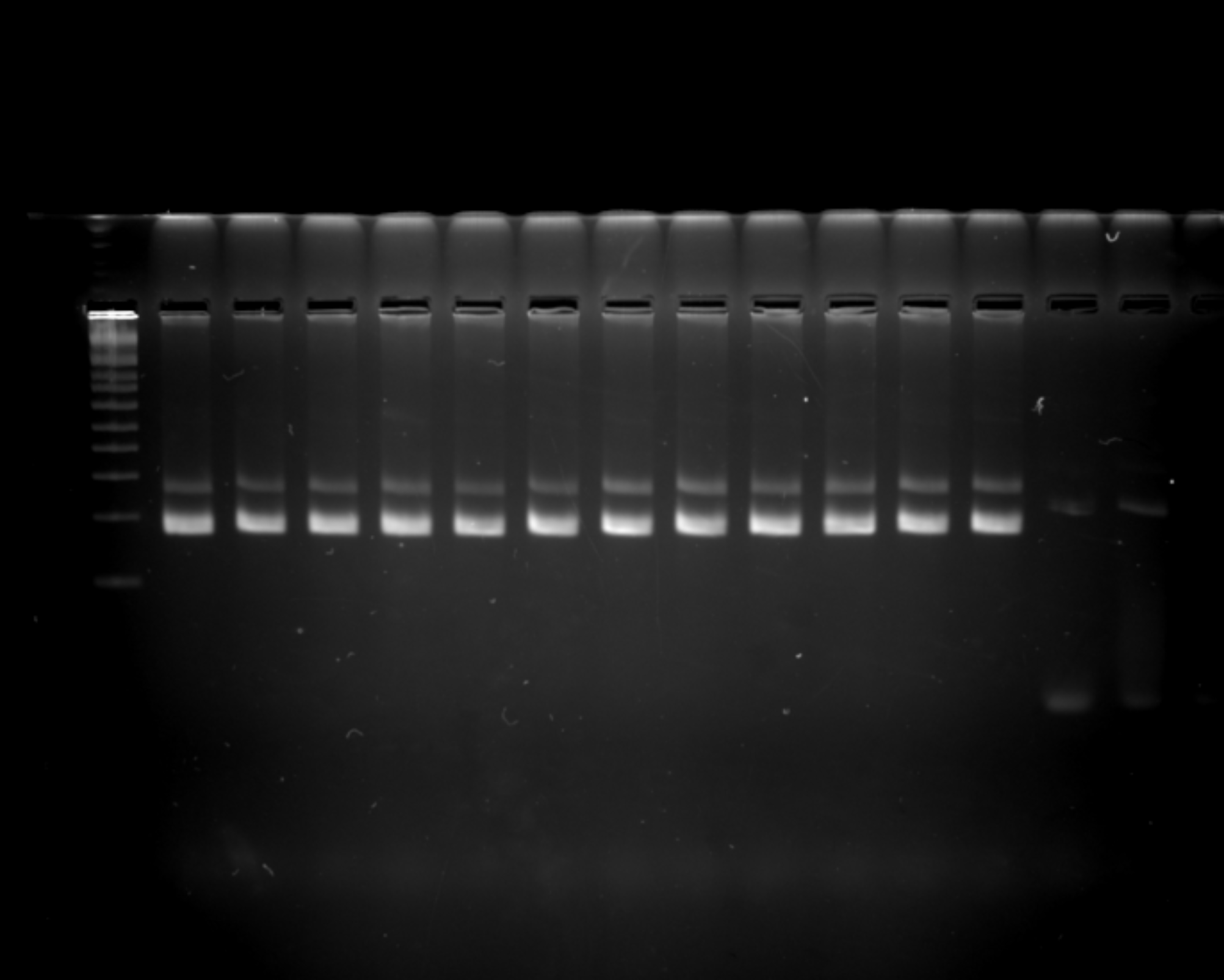

Supplement: Supplementary file 12 — Source Data [file 41467_2023_38273_MOESM12_ESM.zip › Source Data/Uncropped images/Fig4c_SYNGAP1_rightpanel.tif]

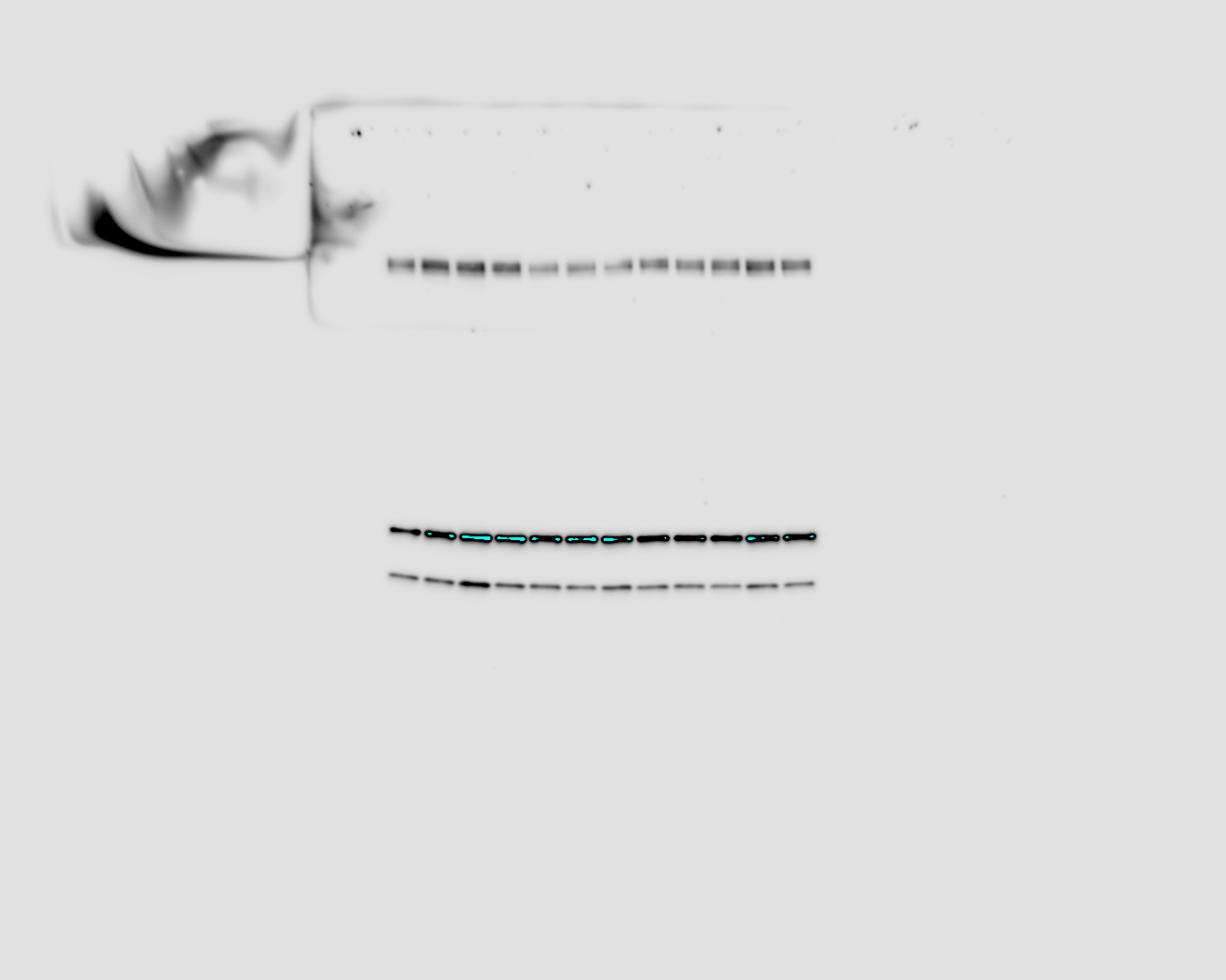

Supplement: Supplementary file 12 — Source Data [file 41467_2023_38273_MOESM12_ESM.zip › Source Data/Uncropped images/Fig4f_SYNGAP1blot_SYNGAP1_GAPDH_ATP5F1.tif]

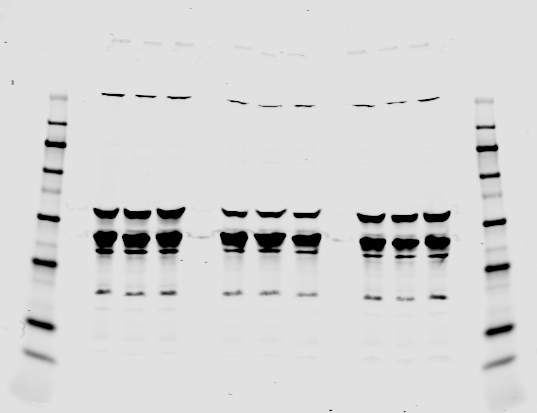

Supplement: Supplementary file 12 — Source Data [file 41467_2023_38273_MOESM12_ESM.zip › Source Data/Uncropped images/Fig5a_PTBP1blot_ATP5F1.tif]

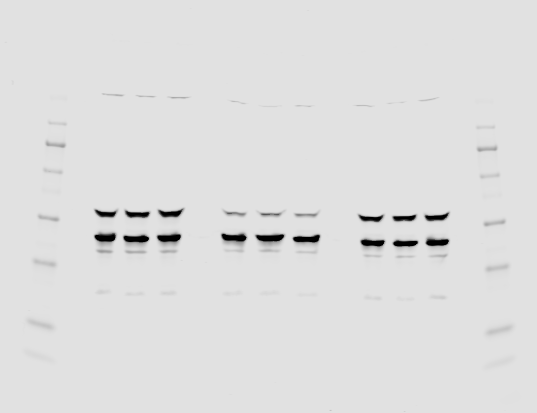

Supplement: Supplementary file 12 — Source Data [file 41467_2023_38273_MOESM12_ESM.zip › Source Data/Uncropped images/Fig5a_PTBP1blot_PTBP1.tif]

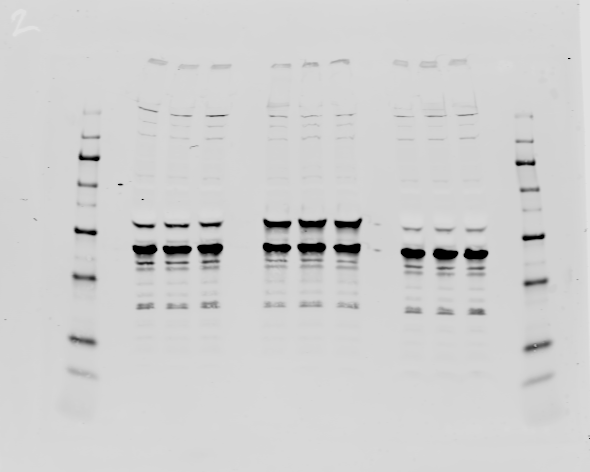

Supplement: Supplementary file 12 — Source Data [file 41467_2023_38273_MOESM12_ESM.zip › Source Data/Uncropped images/Fig5a_PTBP2blot_ATP5F1.tif]

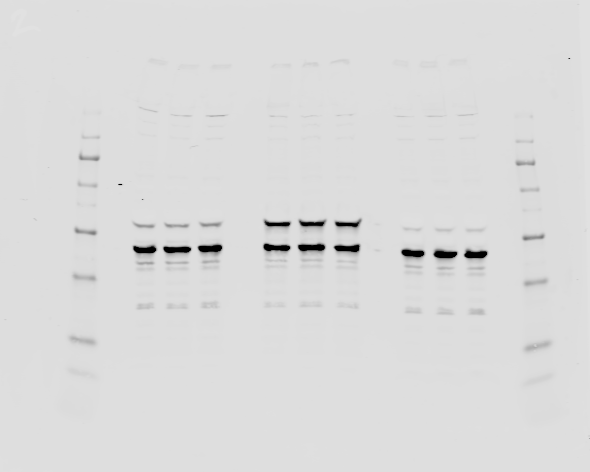

Supplement: Supplementary file 12 — Source Data [file 41467_2023_38273_MOESM12_ESM.zip › Source Data/Uncropped images/Fig5a_PTBP2blot_PTBP2.tif]

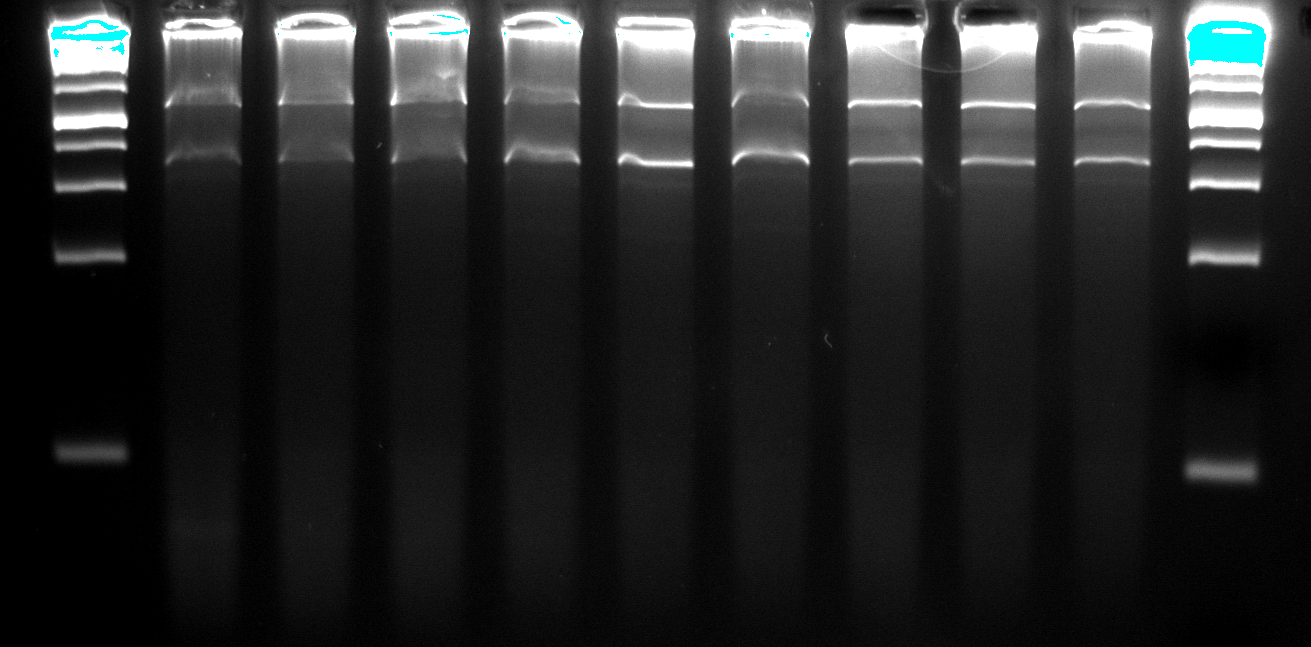

Supplement: Supplementary file 12 — Source Data [file 41467_2023_38273_MOESM12_ESM.zip › Source Data/Uncropped images/Fig5b_SYNGAP1_gel1.tif]

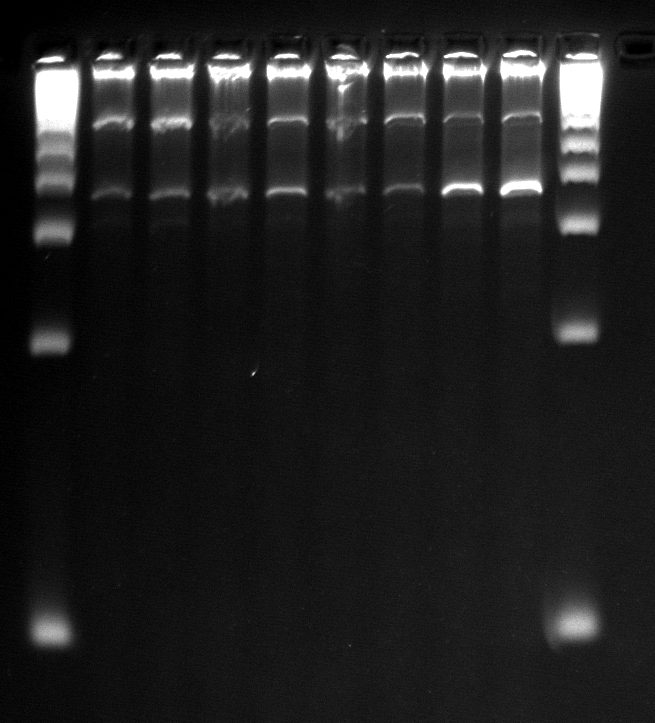

Supplement: Supplementary file 12 — Source Data [file 41467_2023_38273_MOESM12_ESM.zip › Source Data/Uncropped images/Fig5b_SYNGAP1_gel2.tif]

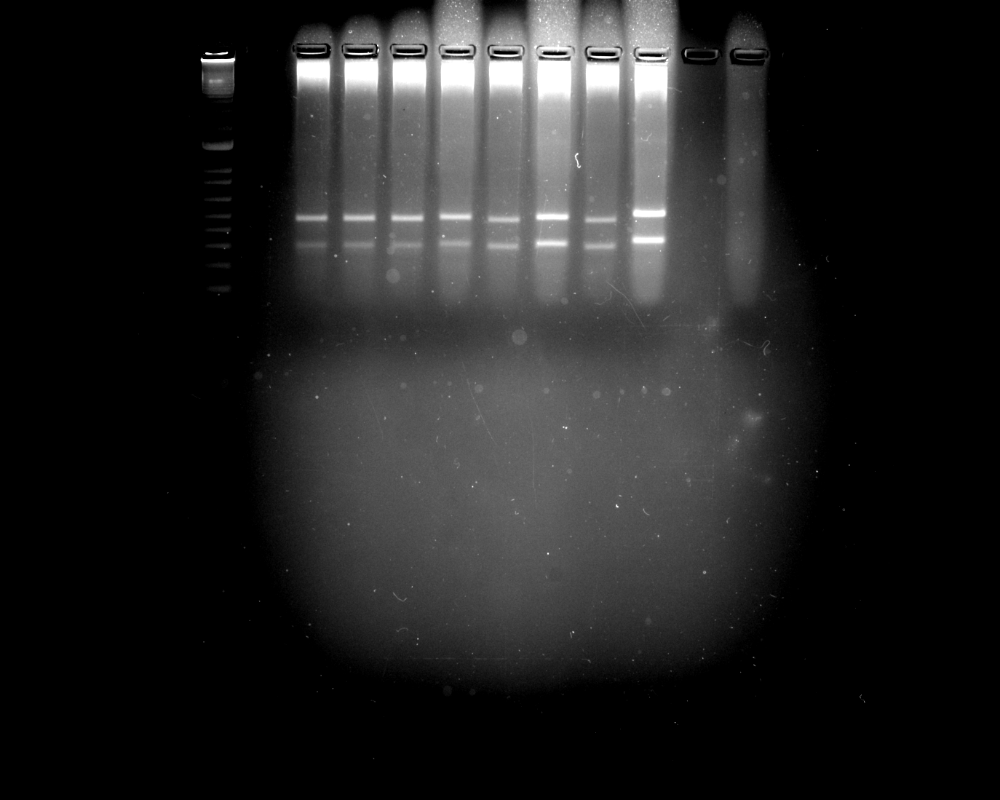

Supplement: Supplementary file 12 — Source Data [file 41467_2023_38273_MOESM12_ESM.zip › Source Data/Uncropped images/Fig5c_SYNGAP1.tif]

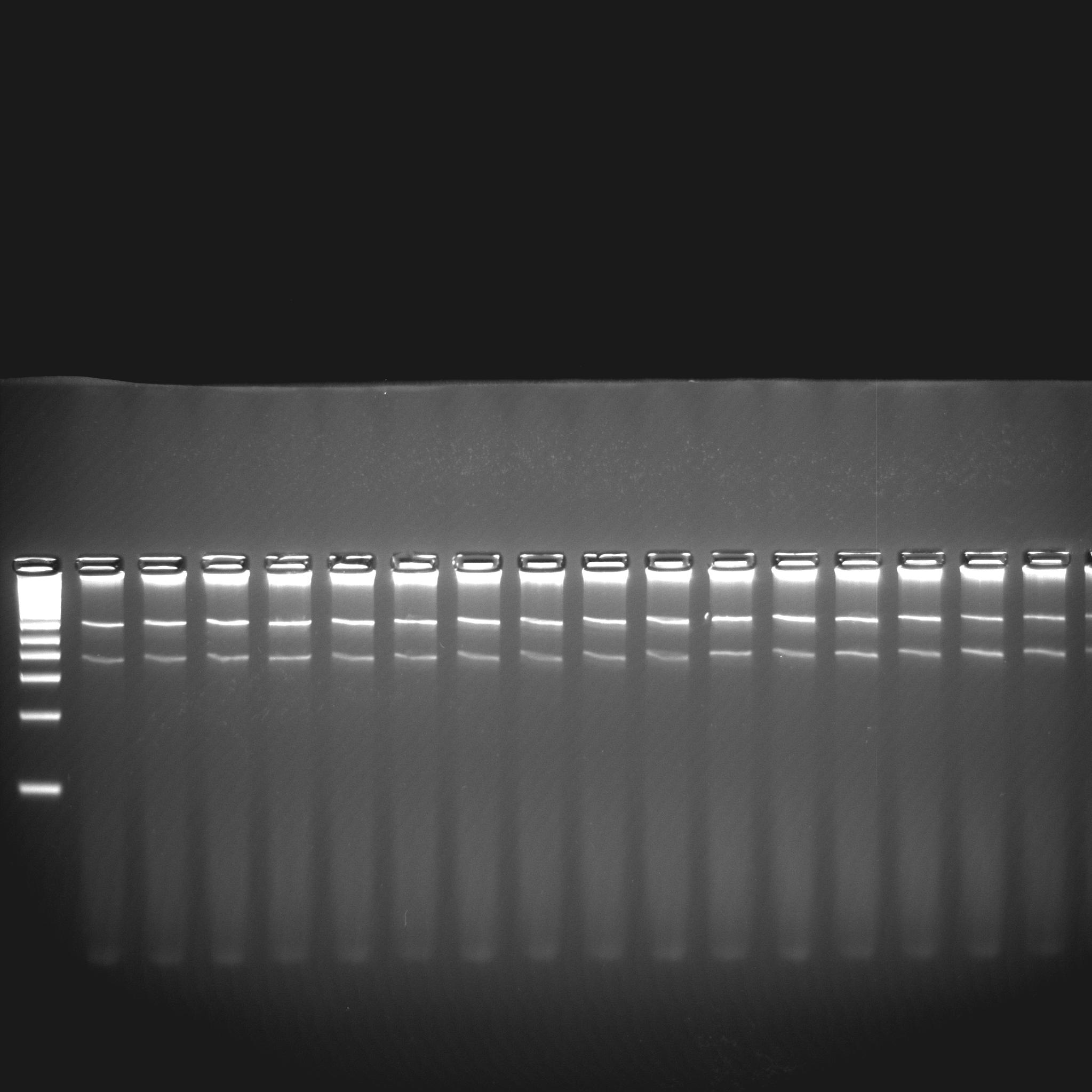

Supplement: Supplementary file 12 — Source Data [file 41467_2023_38273_MOESM12_ESM.zip › Source Data/Uncropped images/Fig5f_SYNGAP1_gel1_part1.tif]

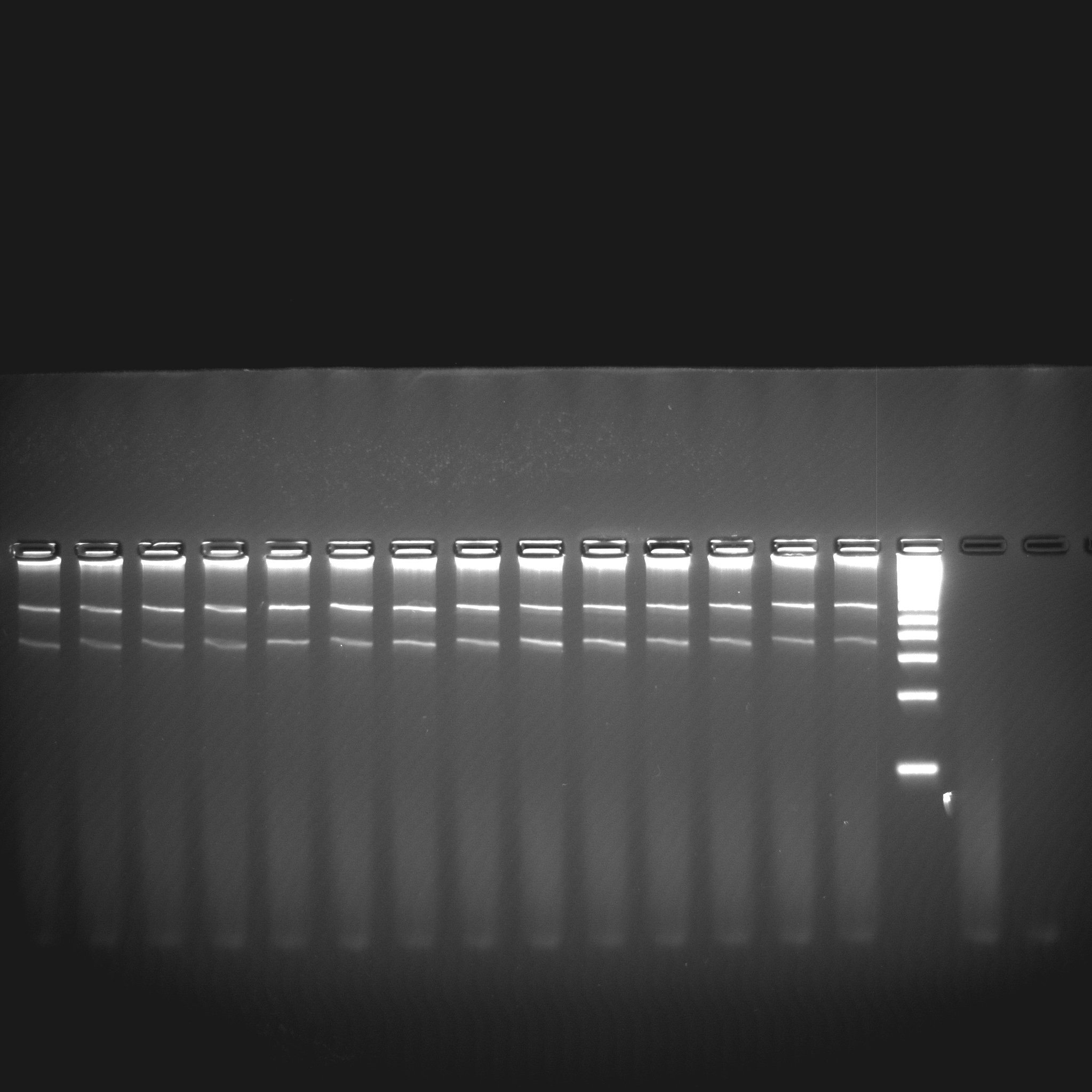

Supplement: Supplementary file 12 — Source Data [file 41467_2023_38273_MOESM12_ESM.zip › Source Data/Uncropped images/Fig5f_SYNGAP1_gel1_part2.tif]

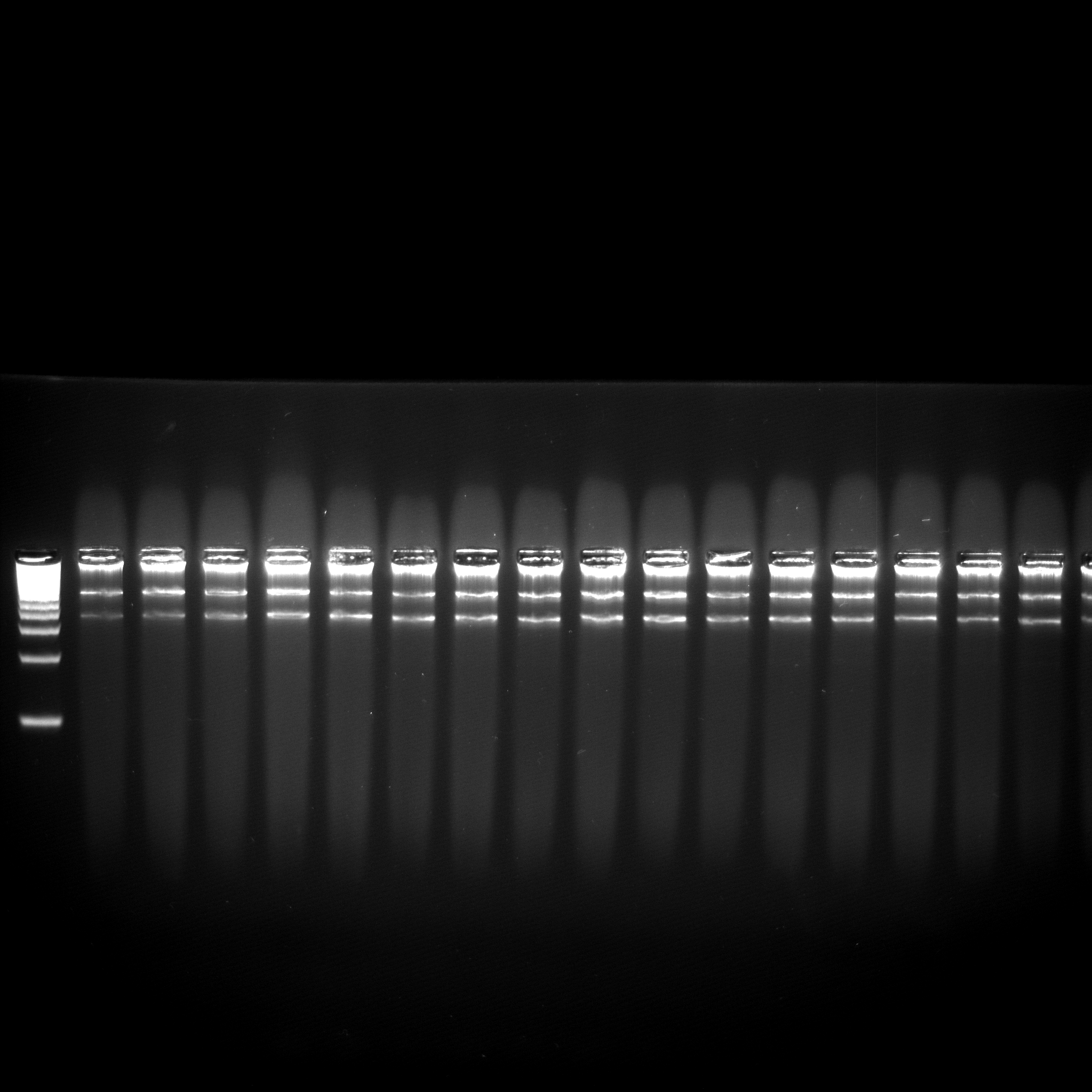

Supplement: Supplementary file 12 — Source Data [file 41467_2023_38273_MOESM12_ESM.zip › Source Data/Uncropped images/Fig5f_SYNGAP1_gel2_part1.tif]

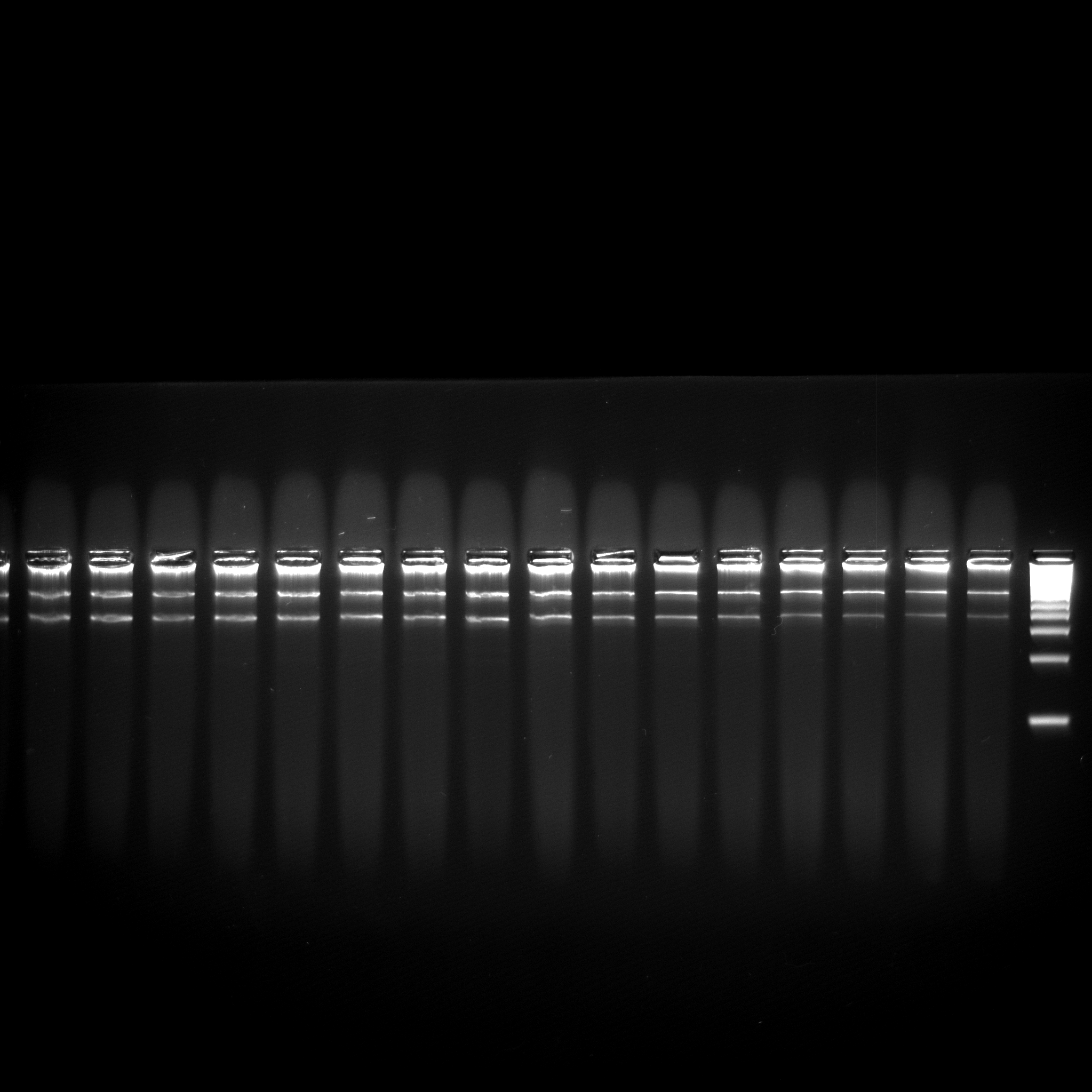

Supplement: Supplementary file 12 — Source Data [file 41467_2023_38273_MOESM12_ESM.zip › Source Data/Uncropped images/Fig5f_SYNGAP1_gel2_part2.tif]

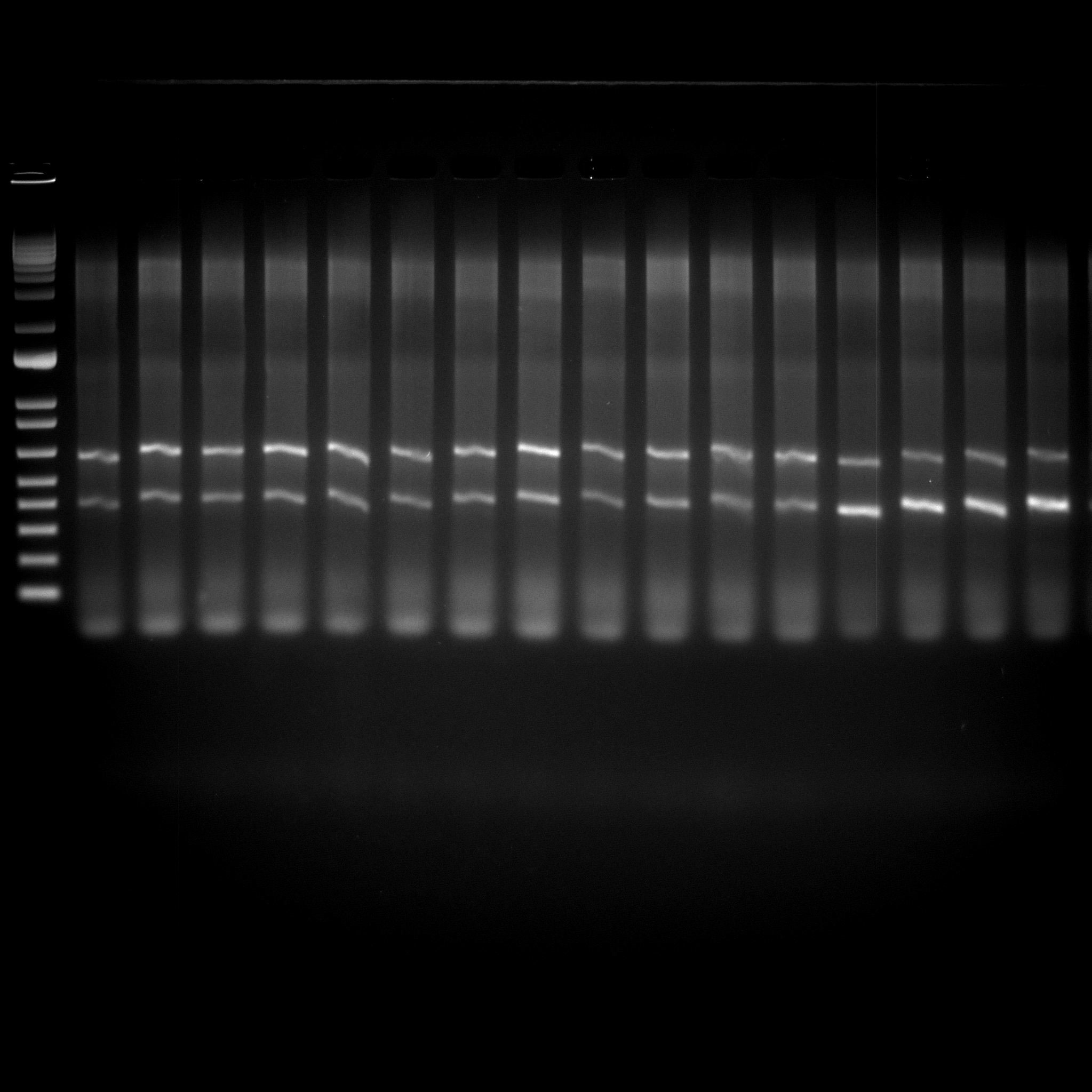

Supplement: Supplementary file 12 — Source Data [file 41467_2023_38273_MOESM12_ESM.zip › Source Data/Uncropped images/Fig5g_SYNGAP1_part1.tif]

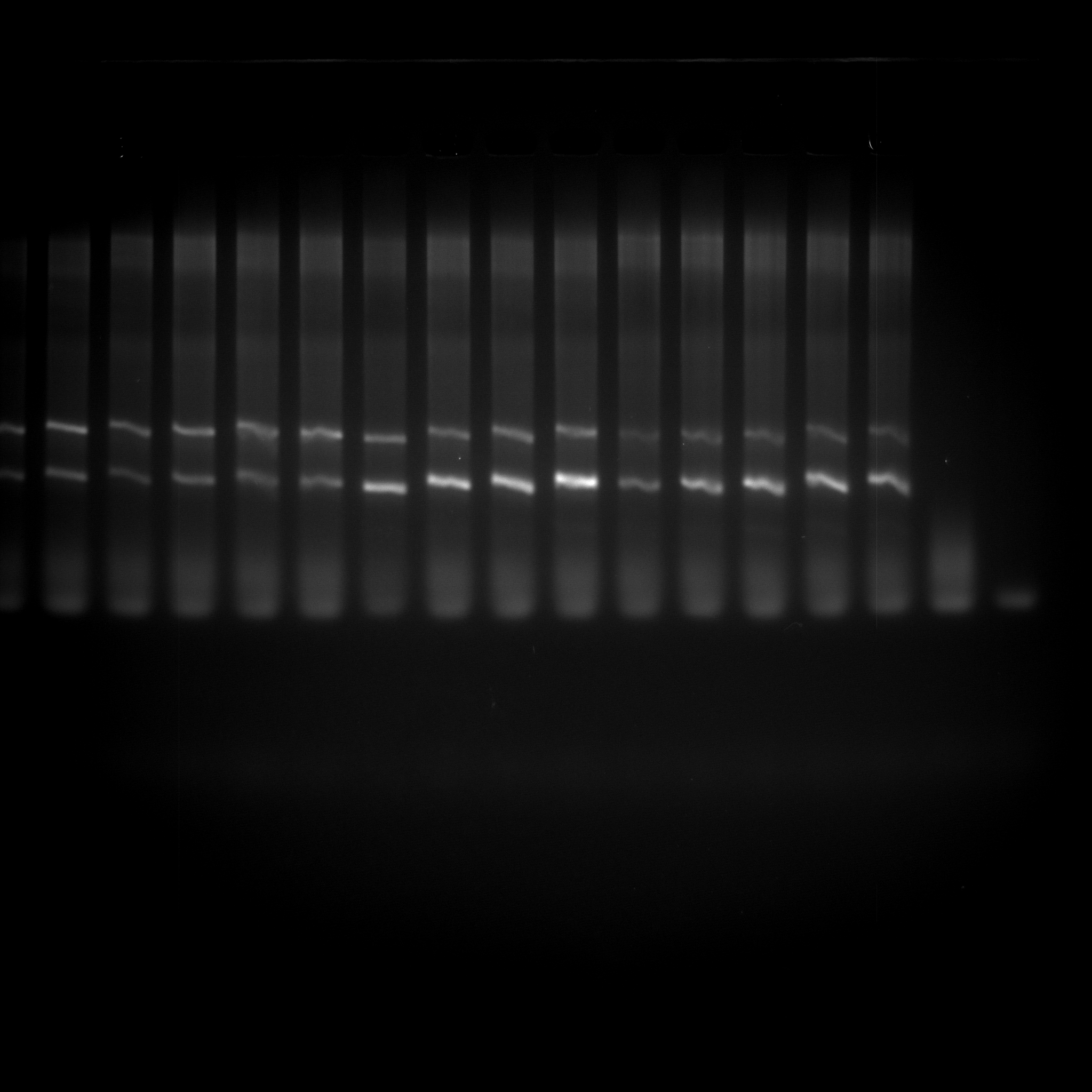

Supplement: Supplementary file 12 — Source Data [file 41467_2023_38273_MOESM12_ESM.zip › Source Data/Uncropped images/Fig5g_SYNGAP1_part2.tif]

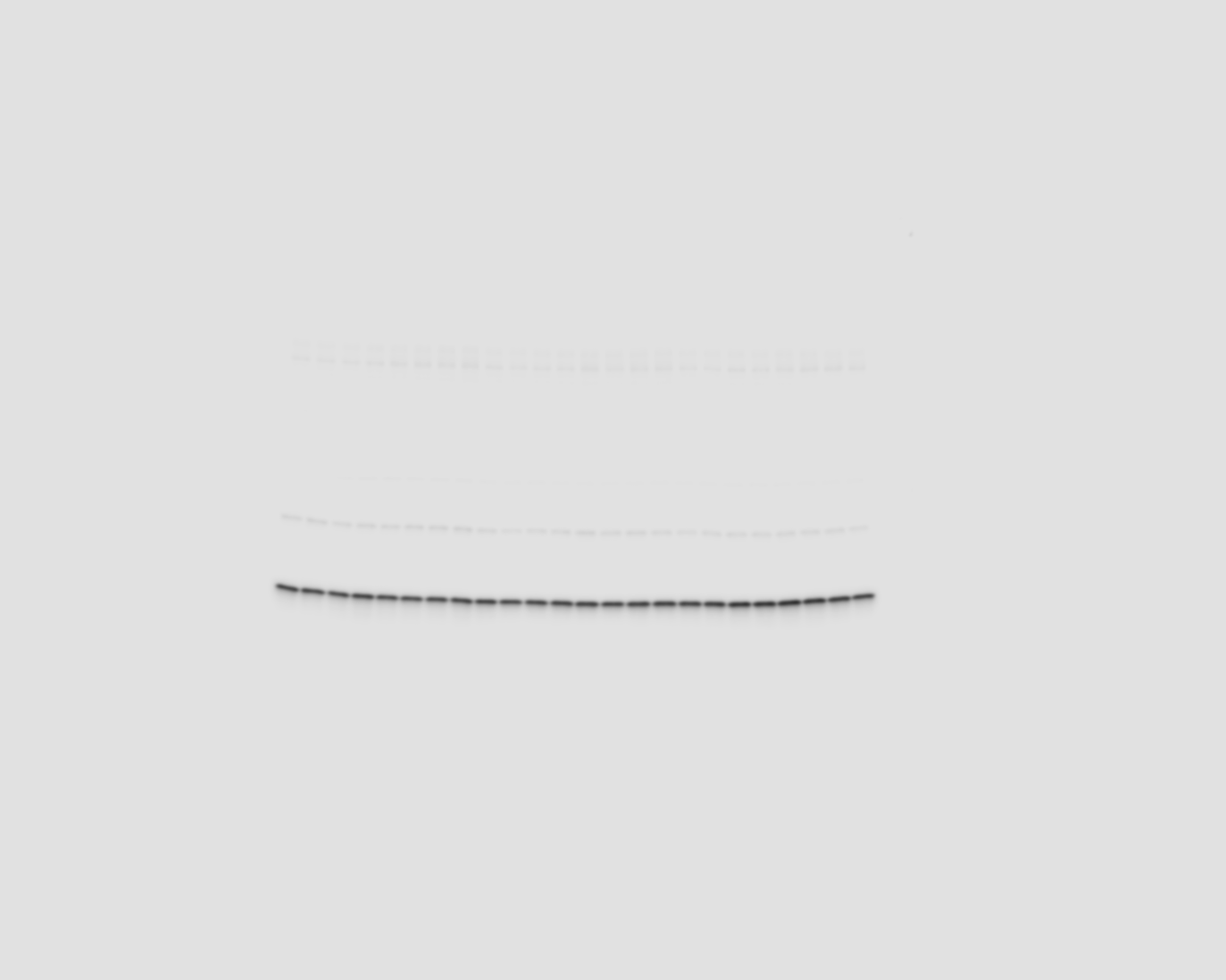

Supplement: Supplementary file 12 — Source Data [file 41467_2023_38273_MOESM12_ESM.zip › Source Data/Uncropped images/Fig5h_SYNGAP1blot_ATP5F1.tif]

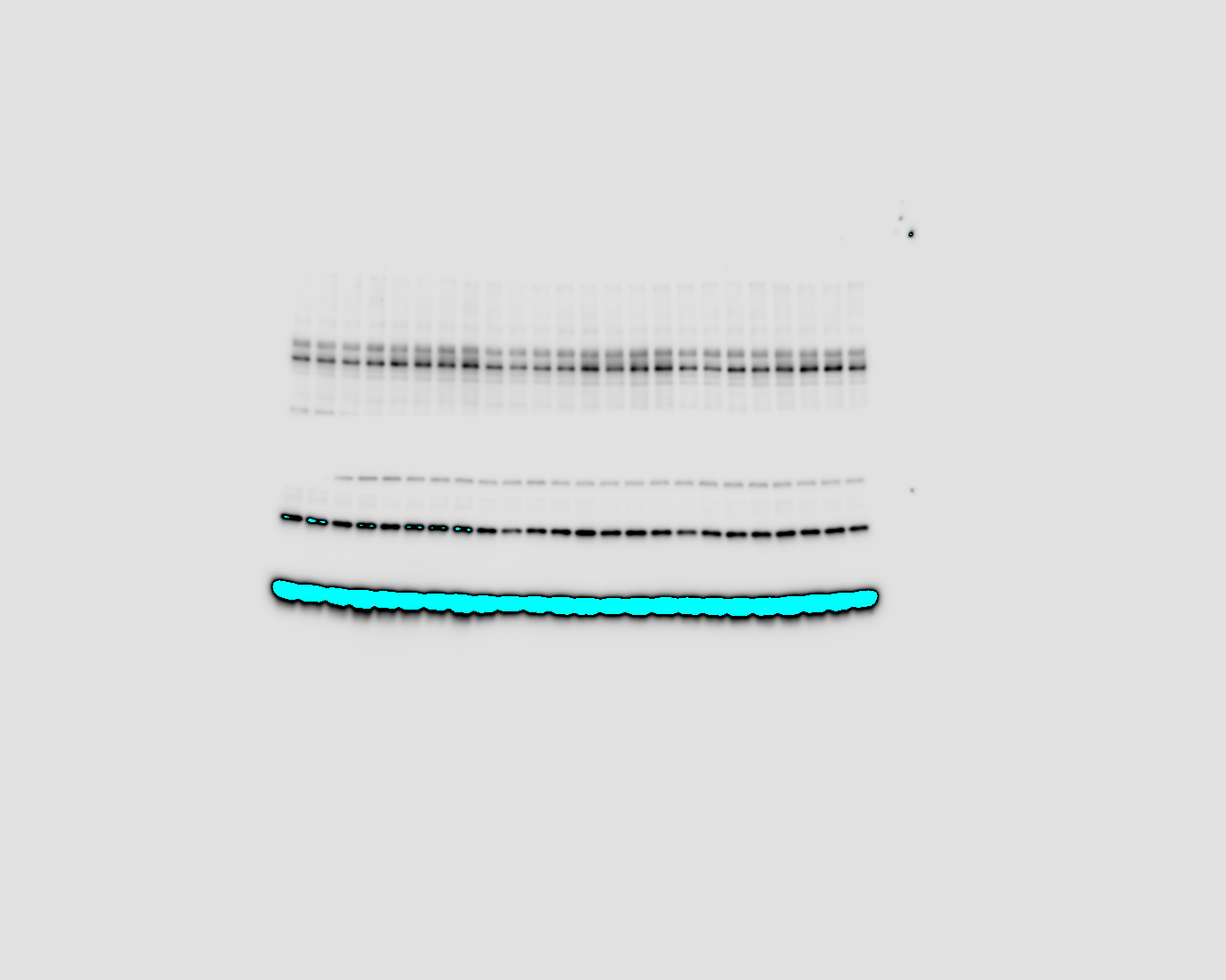

Supplement: Supplementary file 12 — Source Data [file 41467_2023_38273_MOESM12_ESM.zip › Source Data/Uncropped images/Fig5h_SYNGAP1blot_SYNGAP1.tif]

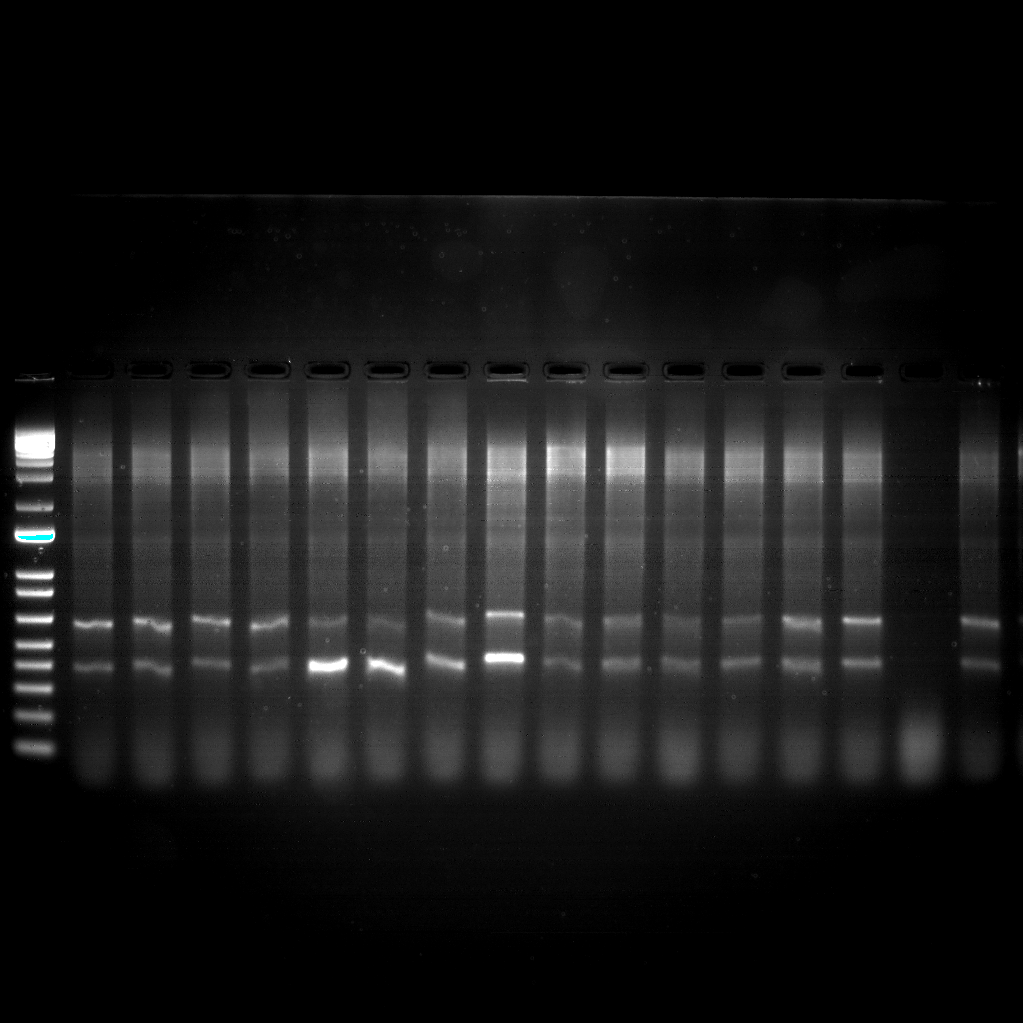

Supplement: Supplementary file 12 — Source Data [file 41467_2023_38273_MOESM12_ESM.zip › Source Data/Uncropped images/Fig6a_SYNGAP1_gel1_part1.tif]

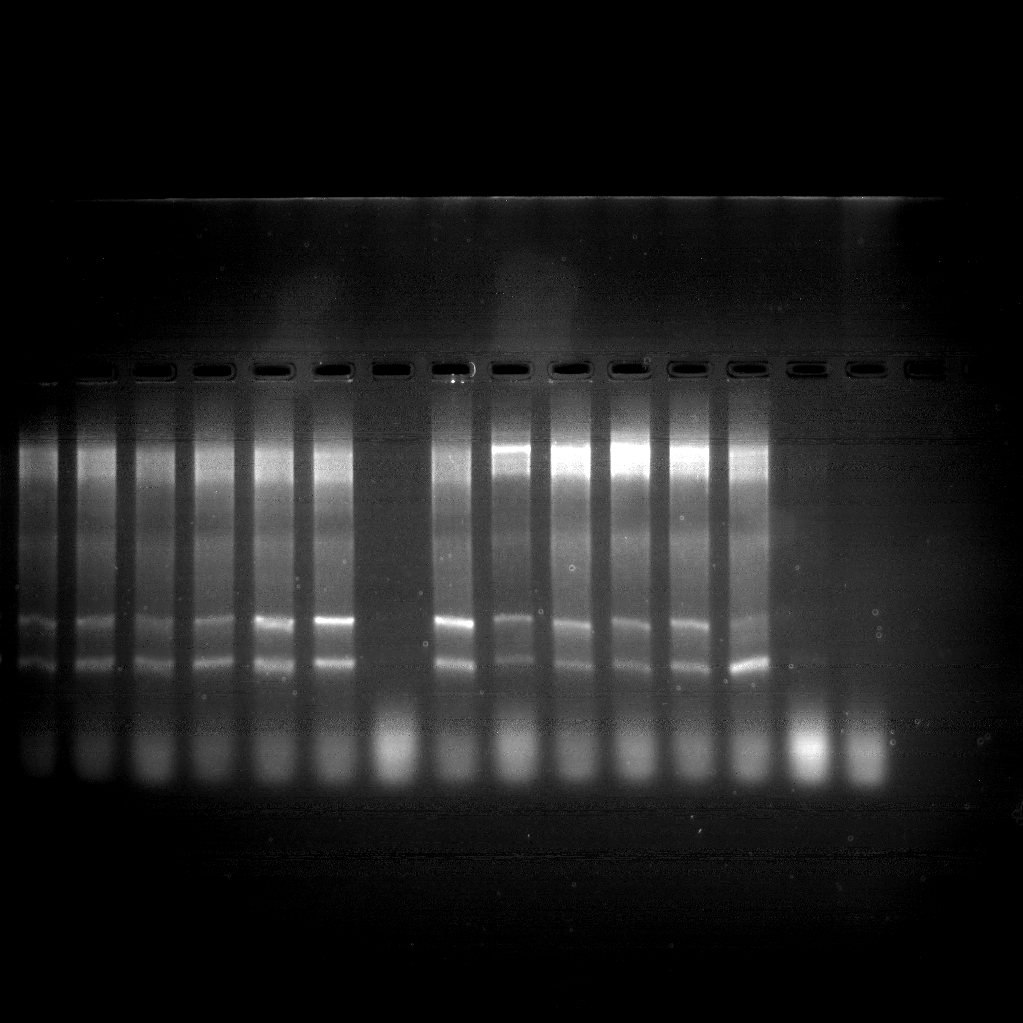

Supplement: Supplementary file 12 — Source Data [file 41467_2023_38273_MOESM12_ESM.zip › Source Data/Uncropped images/Fig6a_SYNGAP1_gel1_part2.tif]

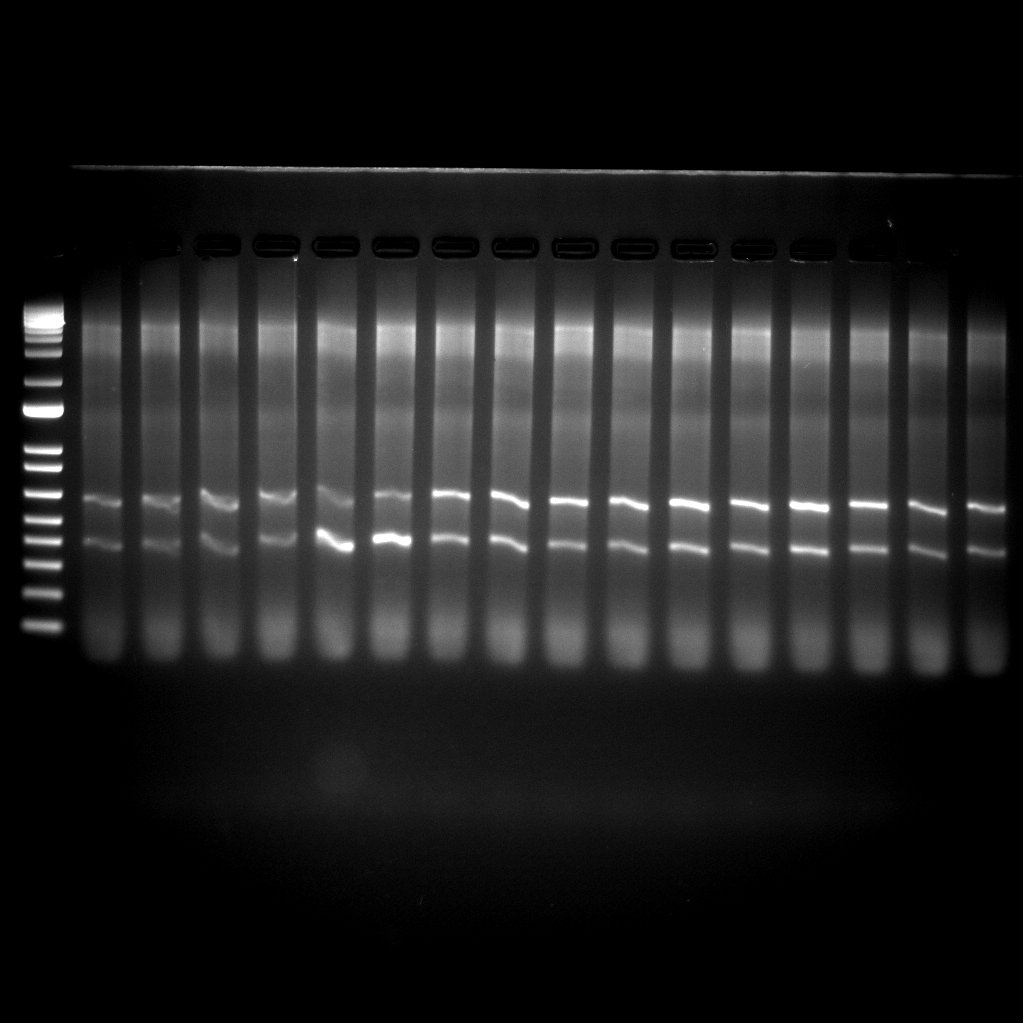

Supplement: Supplementary file 12 — Source Data [file 41467_2023_38273_MOESM12_ESM.zip › Source Data/Uncropped images/Fig6a_SYNGAP1_gel2_part1.tif]

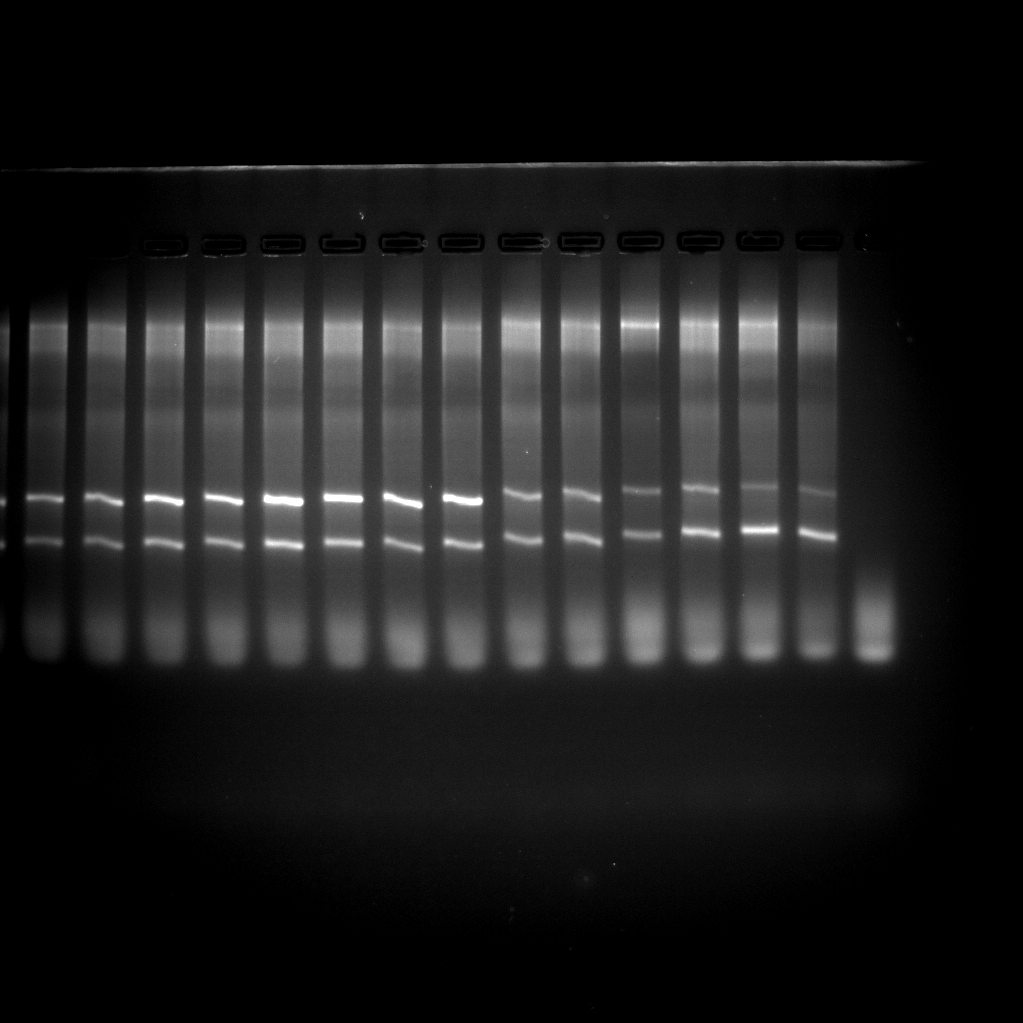

Supplement: Supplementary file 12 — Source Data [file 41467_2023_38273_MOESM12_ESM.zip › Source Data/Uncropped images/Fig6a_SYNGAP1_gel2_part2.tif]

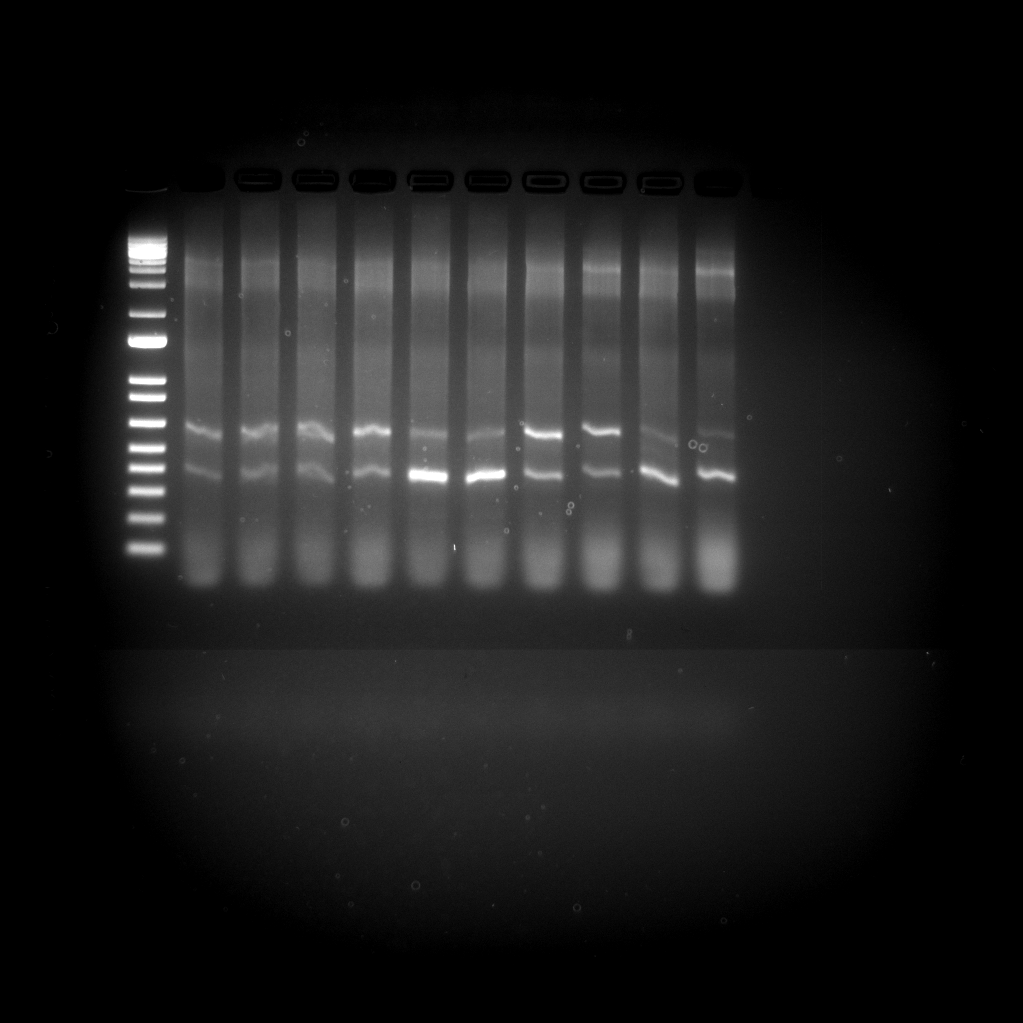

Supplement: Supplementary file 12 — Source Data [file 41467_2023_38273_MOESM12_ESM.zip › Source Data/Uncropped images/Fig6a_SYNGAP1_gel3.tif]

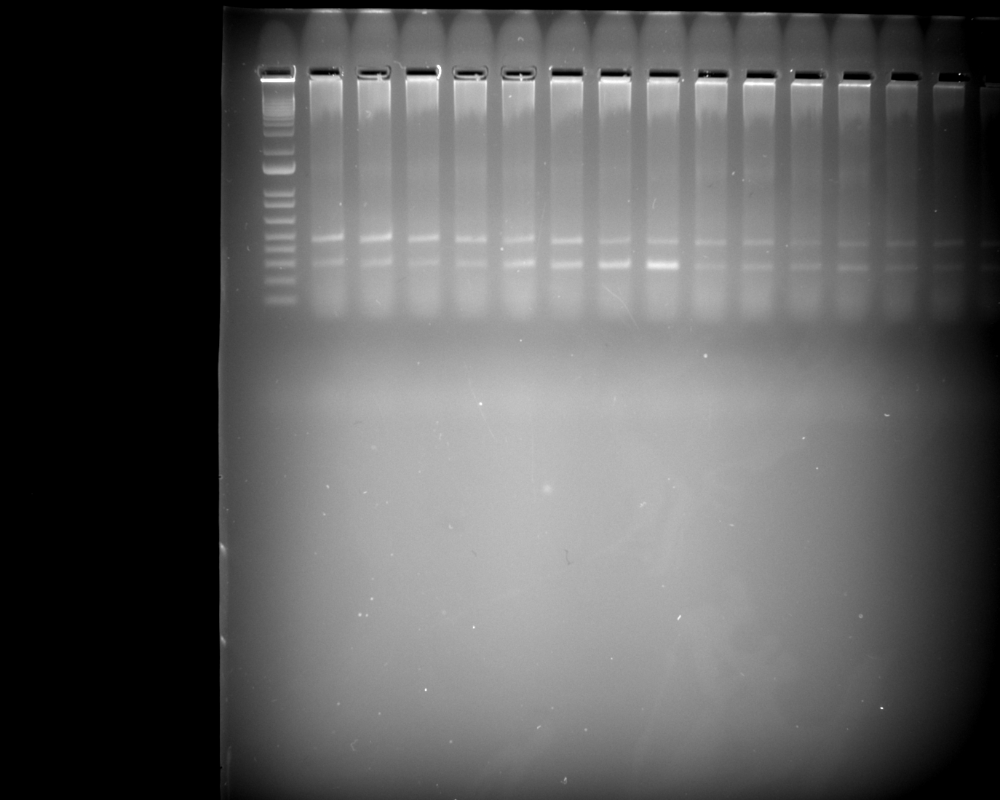

Supplement: Supplementary file 12 — Source Data [file 41467_2023_38273_MOESM12_ESM.zip › Source Data/Uncropped images/Fig6b_SYNGAP1_gel1_part1.tif]

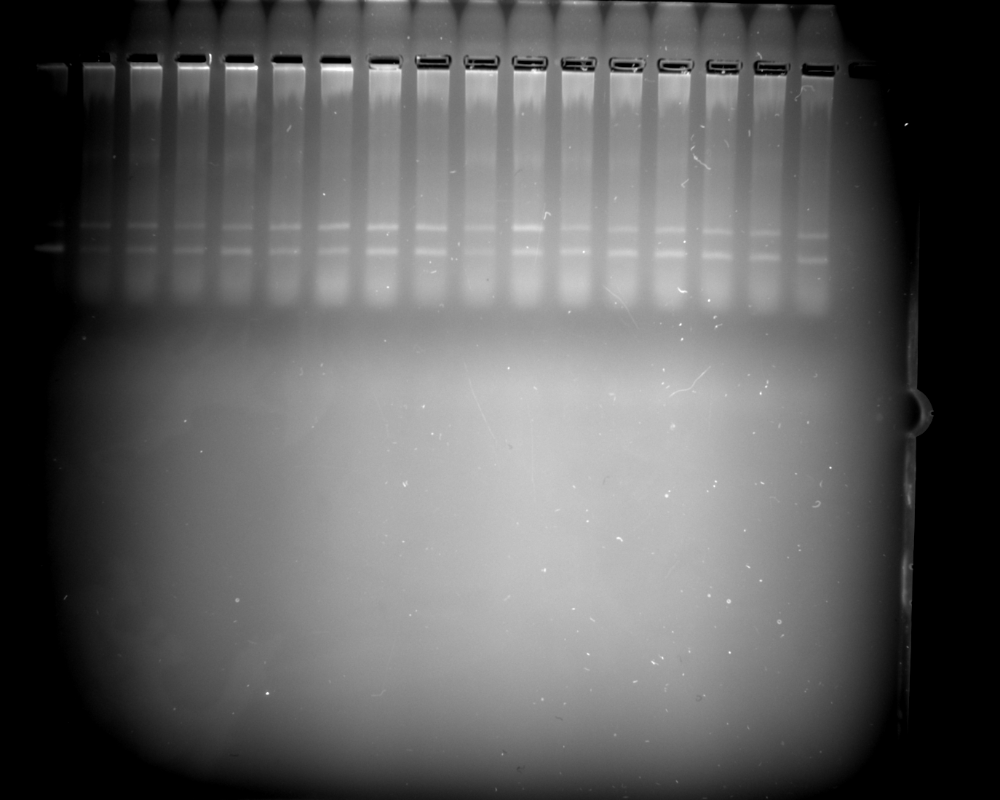

Supplement: Supplementary file 12 — Source Data [file 41467_2023_38273_MOESM12_ESM.zip › Source Data/Uncropped images/Fig6b_SYNGAP1_gel1_part2.tif]

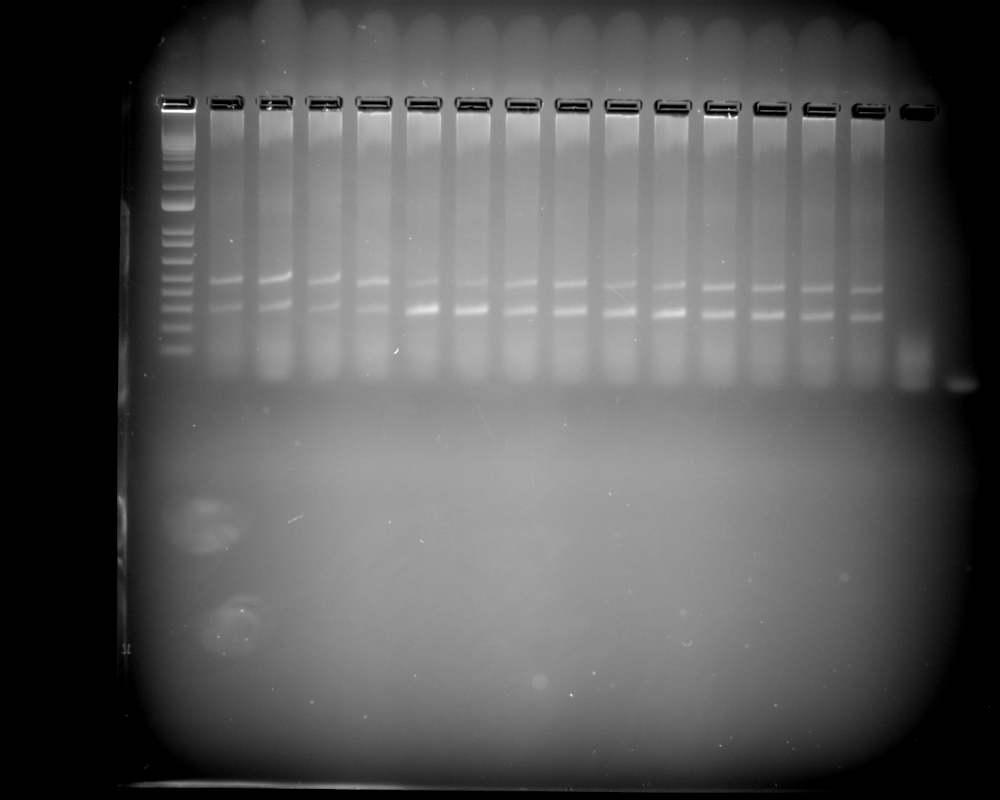

Supplement: Supplementary file 12 — Source Data [file 41467_2023_38273_MOESM12_ESM.zip › Source Data/Uncropped images/Fig6b_SYNGAP1_gel2.tif]

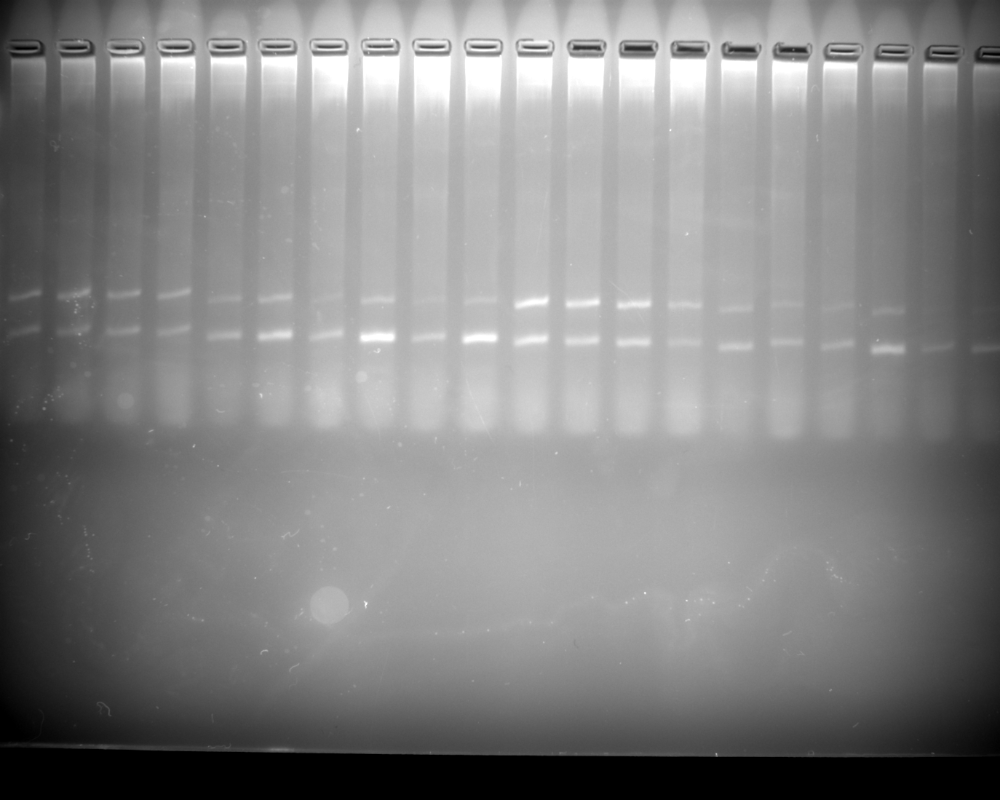

Supplement: Supplementary file 12 — Source Data [file 41467_2023_38273_MOESM12_ESM.zip › Source Data/Uncropped images/Fig6e_SYNGAP1_gel1_part1.tif]

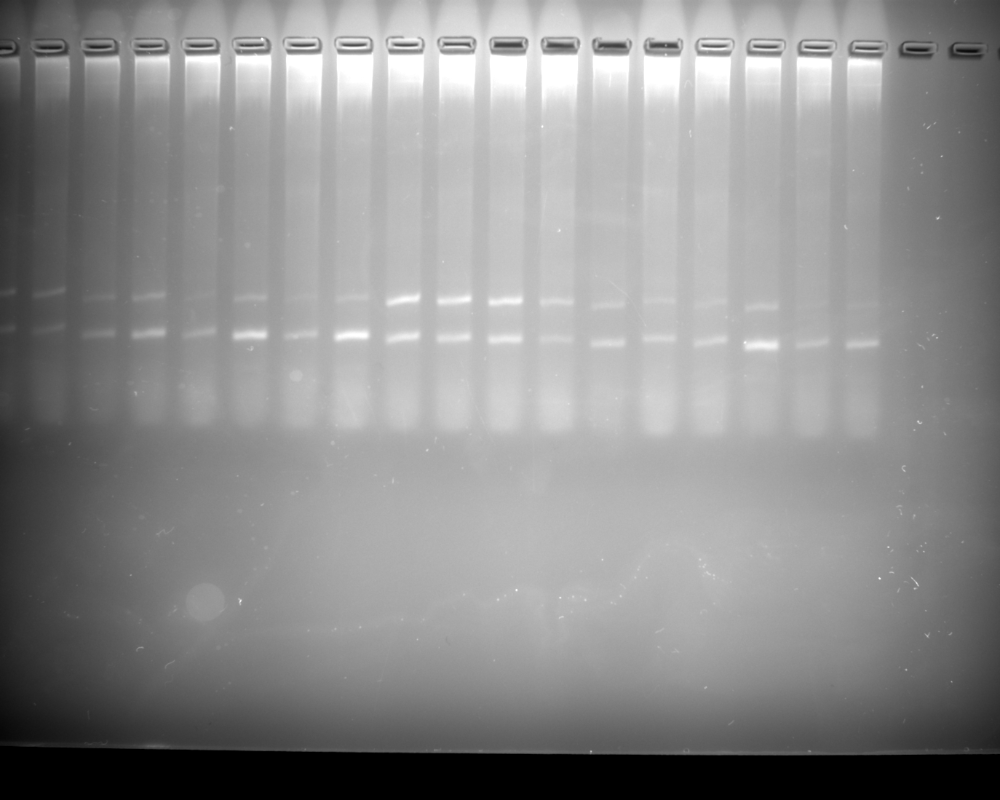

Supplement: Supplementary file 12 — Source Data [file 41467_2023_38273_MOESM12_ESM.zip › Source Data/Uncropped images/Fig6e_SYNGAP1_gel1_part2.tif]

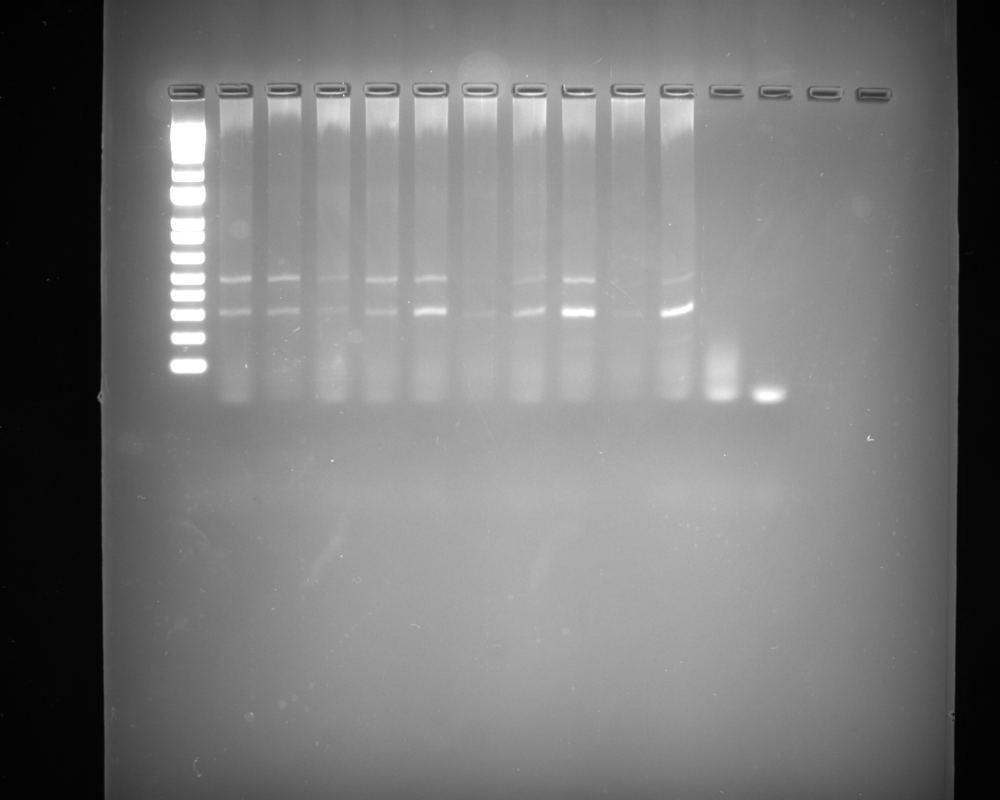

Supplement: Supplementary file 12 — Source Data [file 41467_2023_38273_MOESM12_ESM.zip › Source Data/Uncropped images/Fig6e_SYNGAP1_gel2.tif]

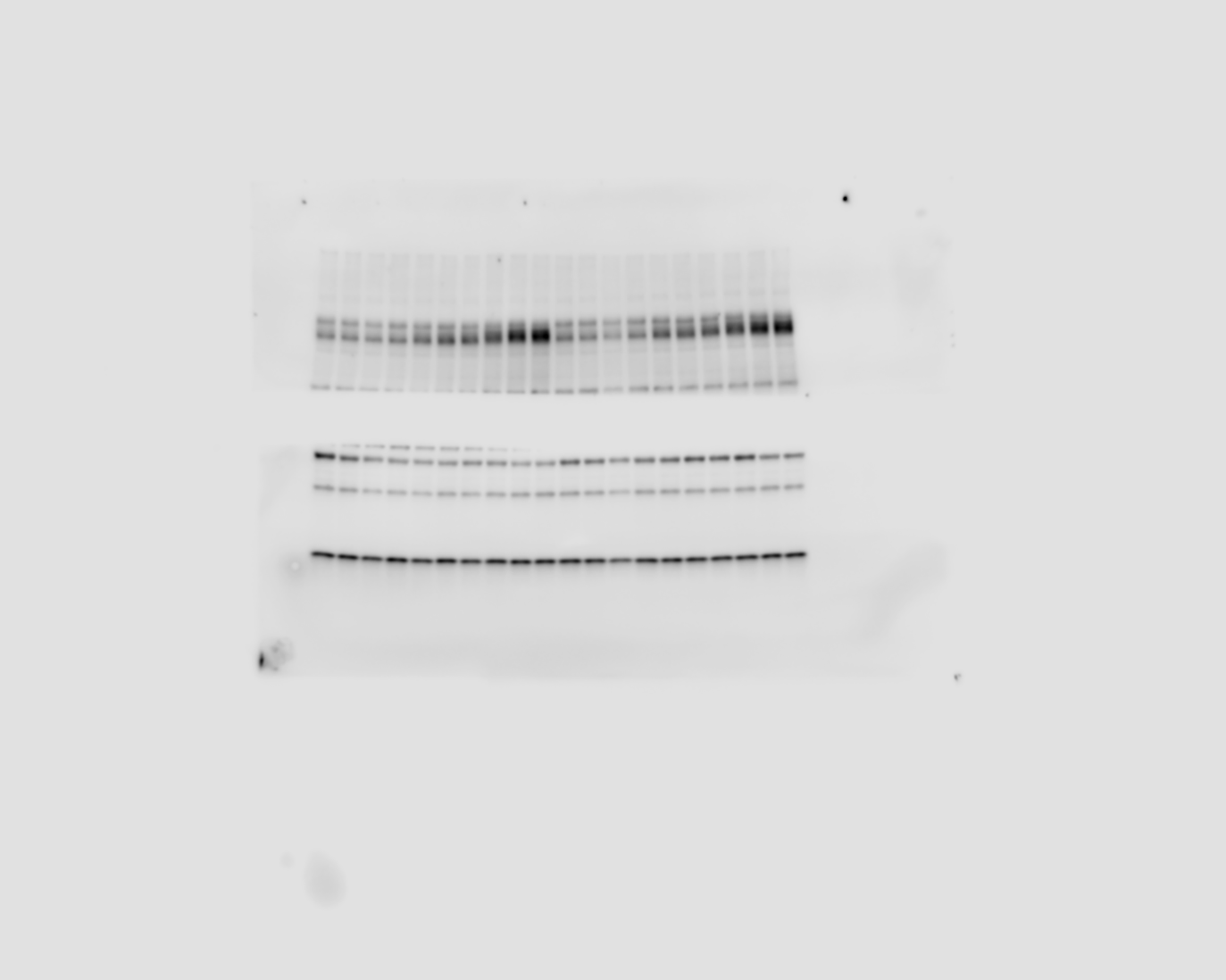

Supplement: Supplementary file 12 — Source Data [file 41467_2023_38273_MOESM12_ESM.zip › Source Data/Uncropped images/Fig6f_SYNGAP1_blot1_SYNGAP1_ATP5F1.tif]

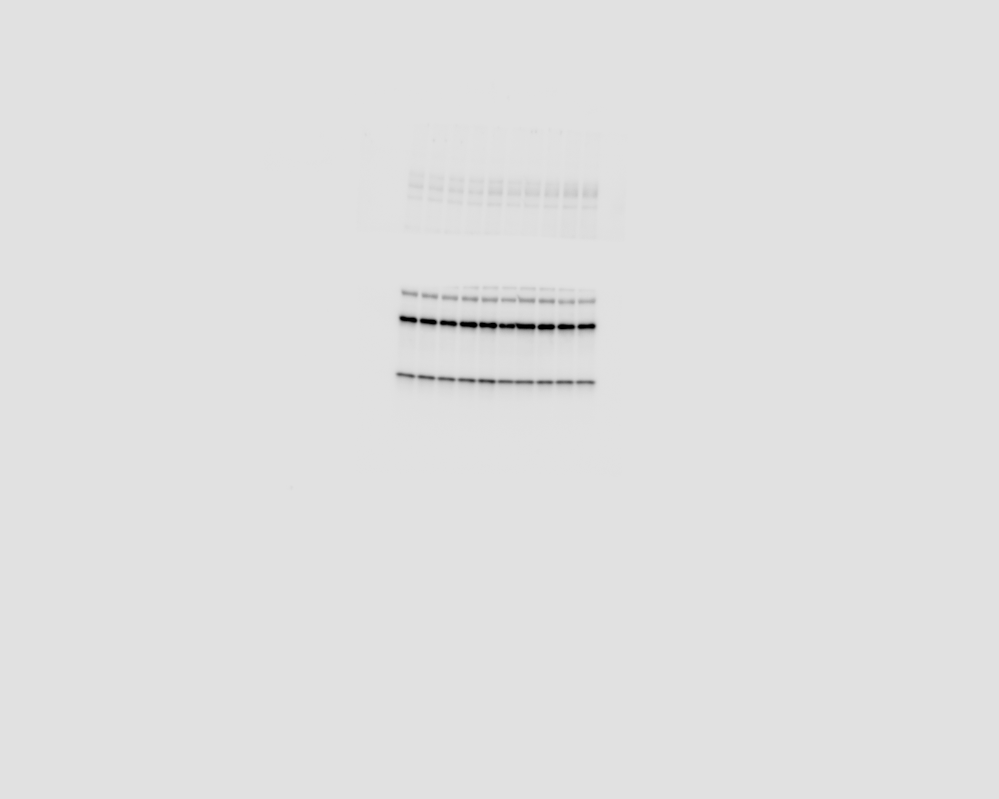

Supplement: Supplementary file 12 — Source Data [file 41467_2023_38273_MOESM12_ESM.zip › Source Data/Uncropped images/Fig6f_SYNGAP1_blot2_ATP5F1.tif]

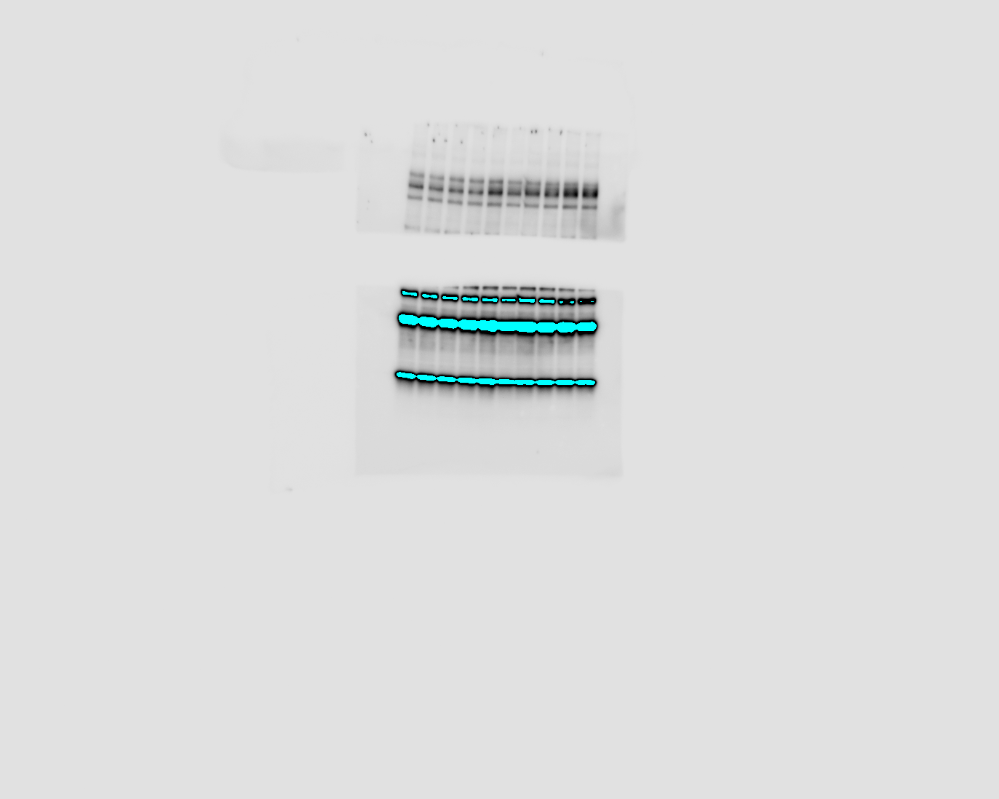

Supplement: Supplementary file 12 — Source Data [file 41467_2023_38273_MOESM12_ESM.zip › Source Data/Uncropped images/Fig6f_SYNGAP1_blot2_SYNGAP1.tif]

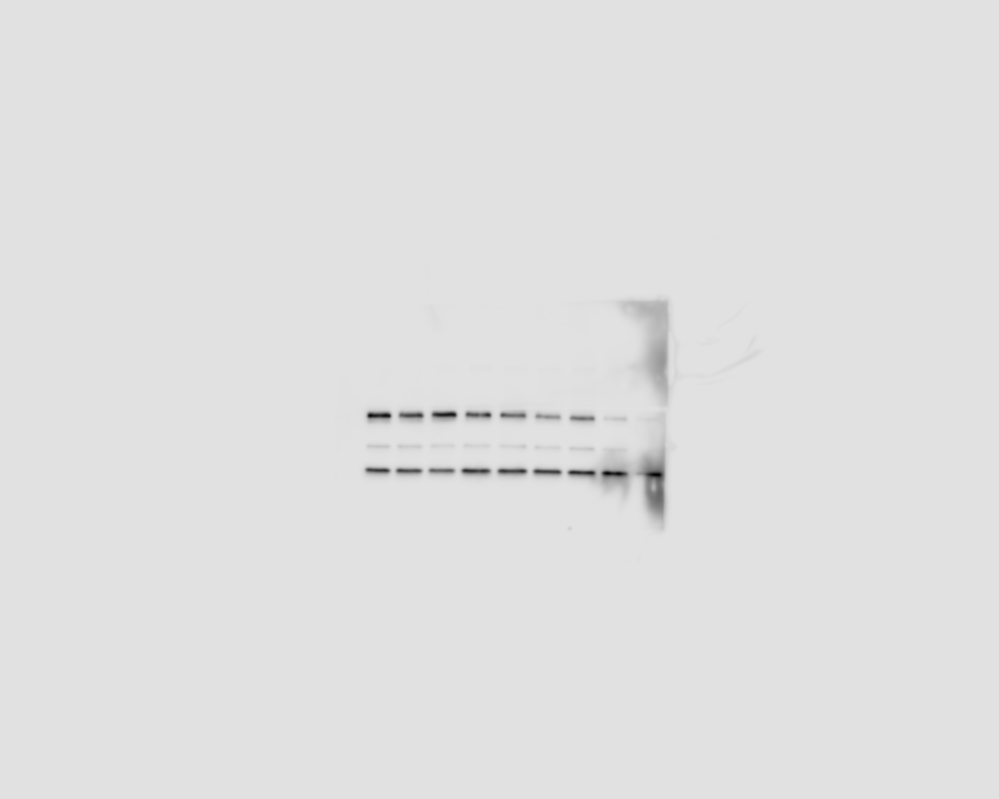

Supplement: Supplementary file 12 — Source Data [file 41467_2023_38273_MOESM12_ESM.zip › Source Data/Uncropped images/Fig7c_SYNGAP1blot_ATP5F1.tif]

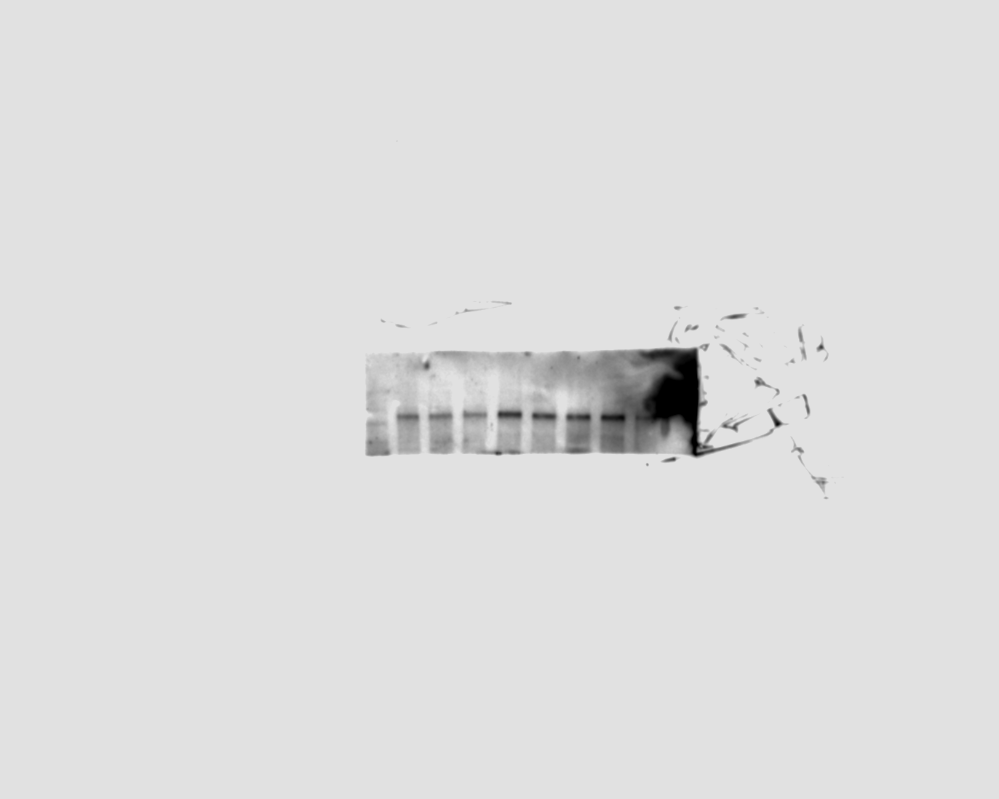

Supplement: Supplementary file 12 — Source Data [file 41467_2023_38273_MOESM12_ESM.zip › Source Data/Uncropped images/Fig7c_SYNGAP1blot_SYNGAP1.tif]

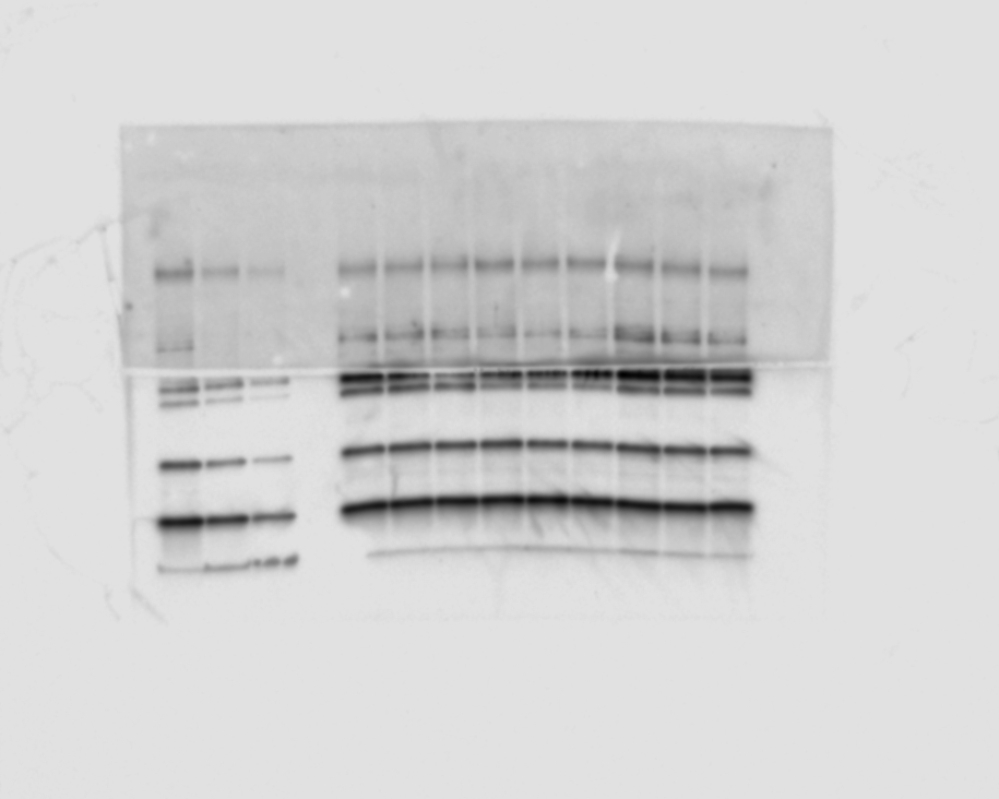

Supplement: Supplementary file 12 — Source Data [file 41467_2023_38273_MOESM12_ESM.zip › Source Data/Uncropped images/Fig7d_SYNGAP1blot_GAPDH.jpg]

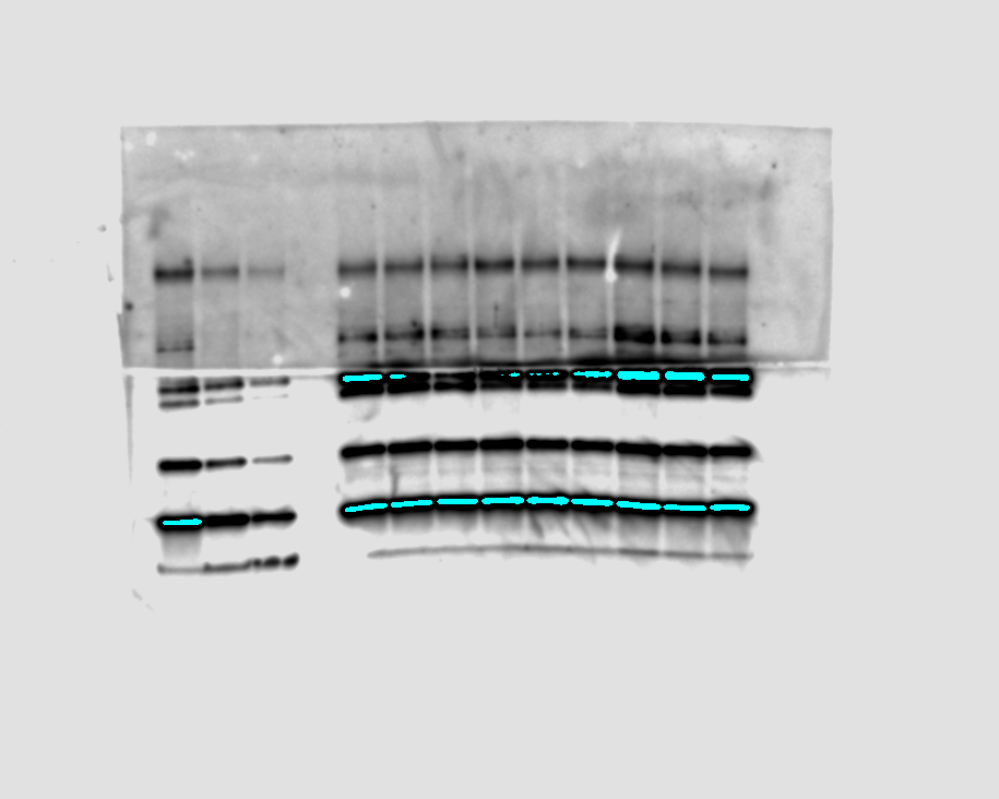

Supplement: Supplementary file 12 — Source Data [file 41467_2023_38273_MOESM12_ESM.zip › Source Data/Uncropped images/Fig7d_SYNGAP1blot_SYNGAP1.tif]

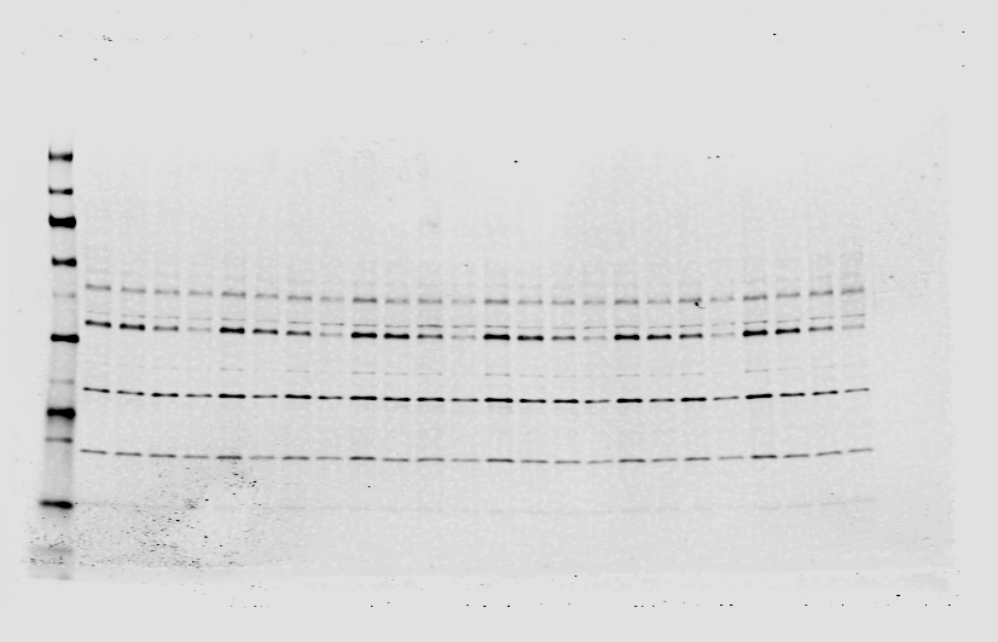

Supplement: Supplementary file 12 — Source Data [file 41467_2023_38273_MOESM12_ESM.zip › Source Data/Uncropped images/Fig7e_PTBP2blot_PTBP2_GAPDH_ATP5F1.tif]

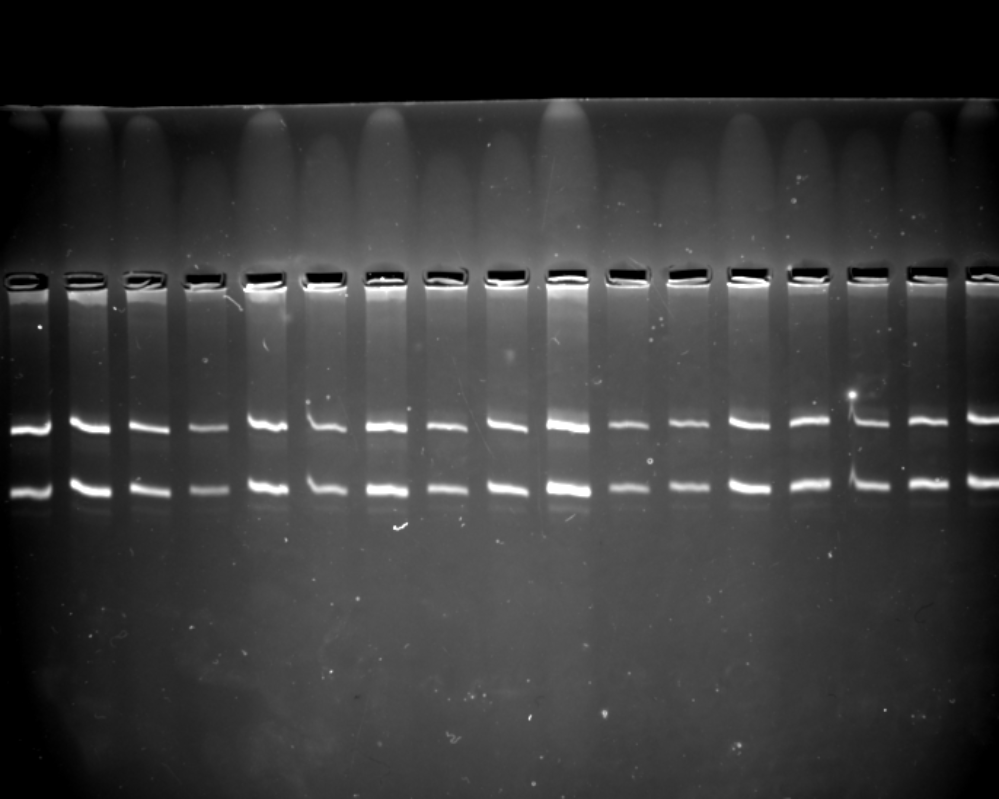

Supplement: Supplementary file 12 — Source Data [file 41467_2023_38273_MOESM12_ESM.zip › Source Data/Uncropped images/Fig7f_SYNGAP1_gel1_part1.tif]

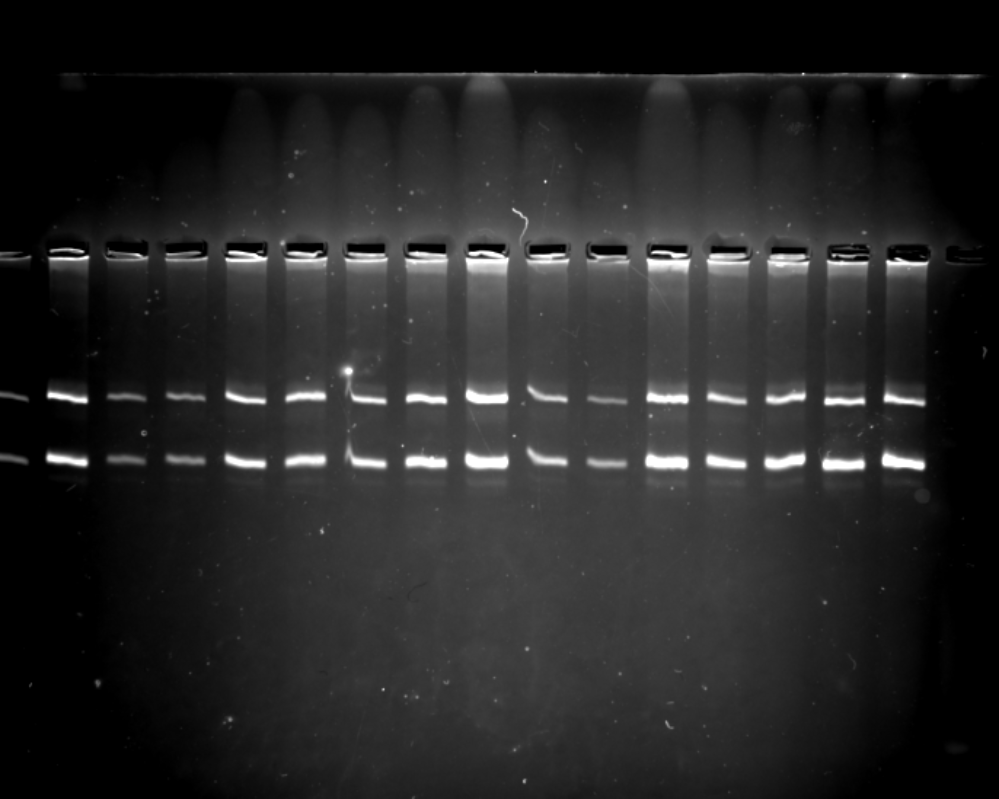

Supplement: Supplementary file 12 — Source Data [file 41467_2023_38273_MOESM12_ESM.zip › Source Data/Uncropped images/Fig7f_SYNGAP1_gel1_part2.tif]

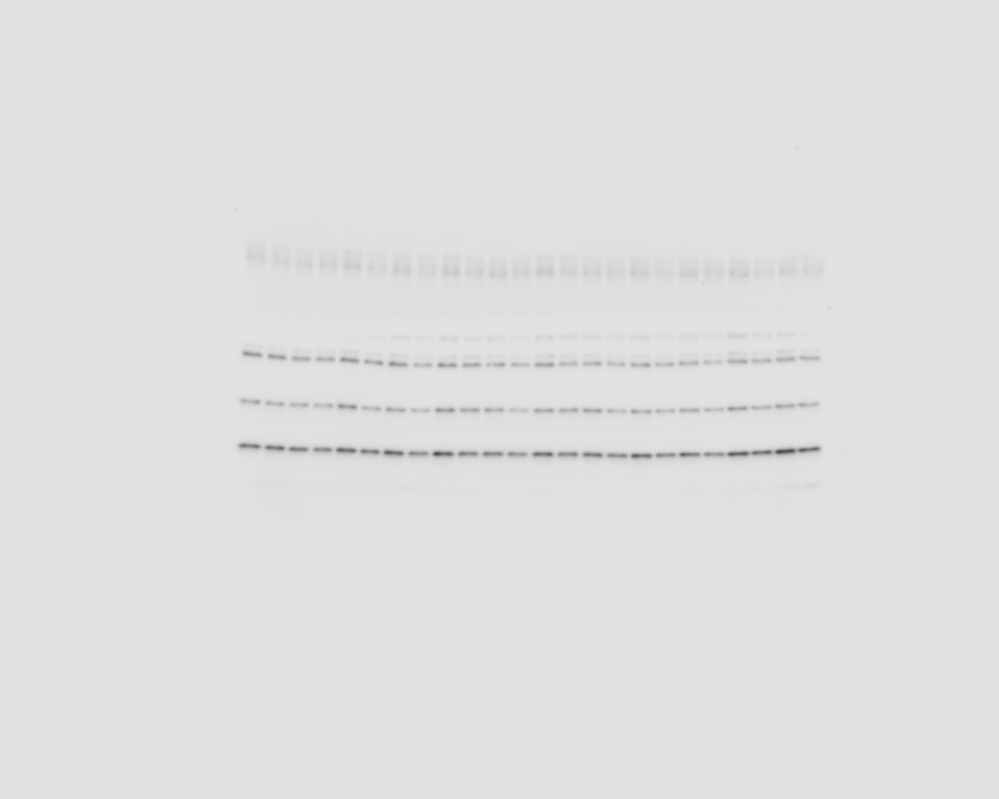

Supplement: Supplementary file 12 — Source Data [file 41467_2023_38273_MOESM12_ESM.zip › Source Data/Uncropped images/Fig7g_SYNGAP1blot_GAPDH_ATP5F1.tif]

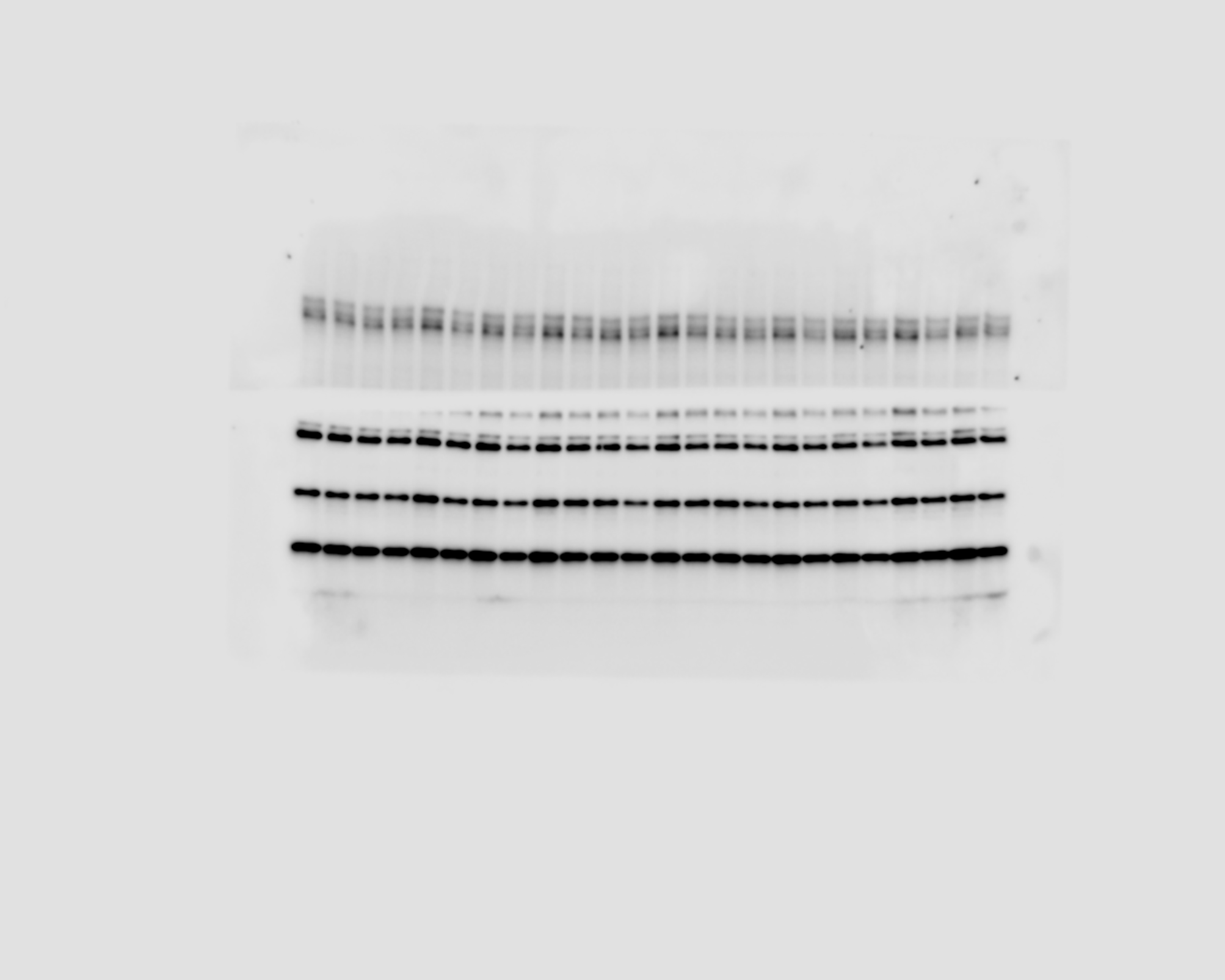

Supplement: Supplementary file 12 — Source Data [file 41467_2023_38273_MOESM12_ESM.zip › Source Data/Uncropped images/Fig7g_SYNGAP1blot_SYNGAP1.tif]

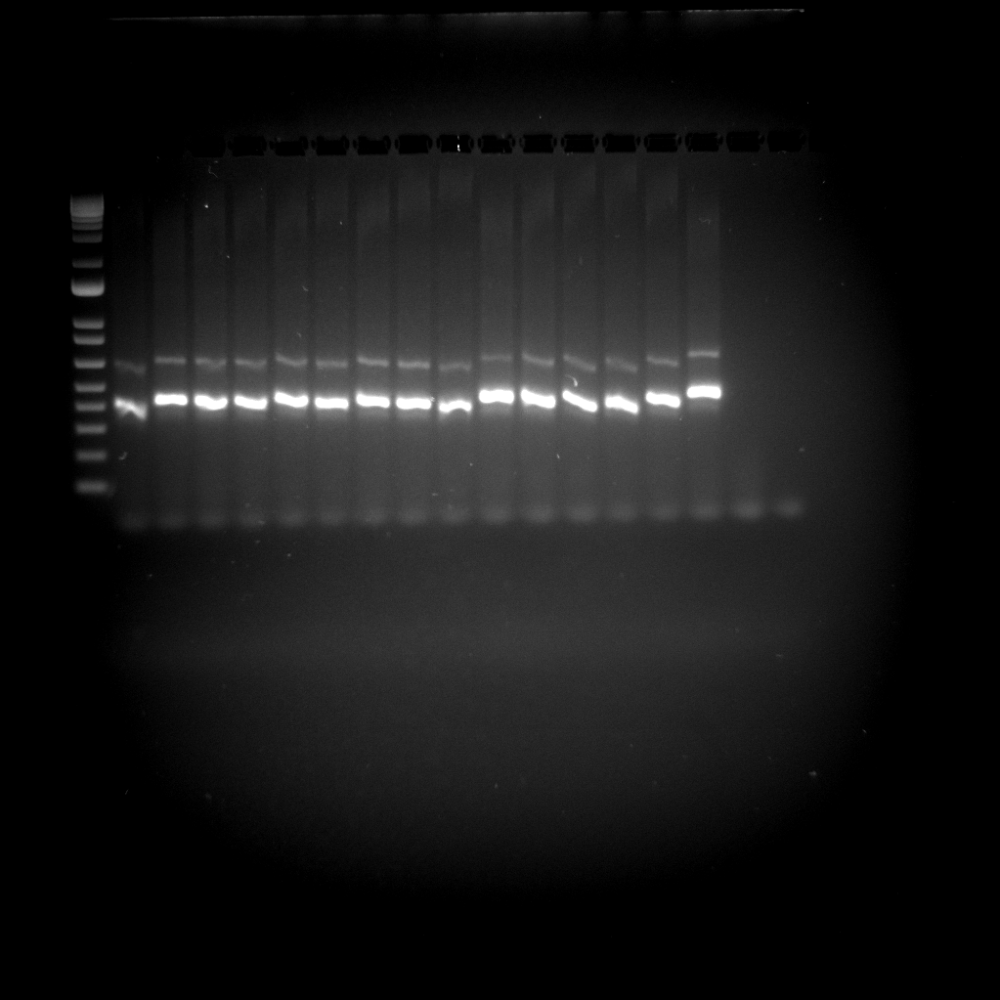

Supplement: Supplementary file 12 — Source Data [file 41467_2023_38273_MOESM12_ESM.zip › Source Data/Uncropped images/Fig7h_SYNGAP1_gel1.tif]

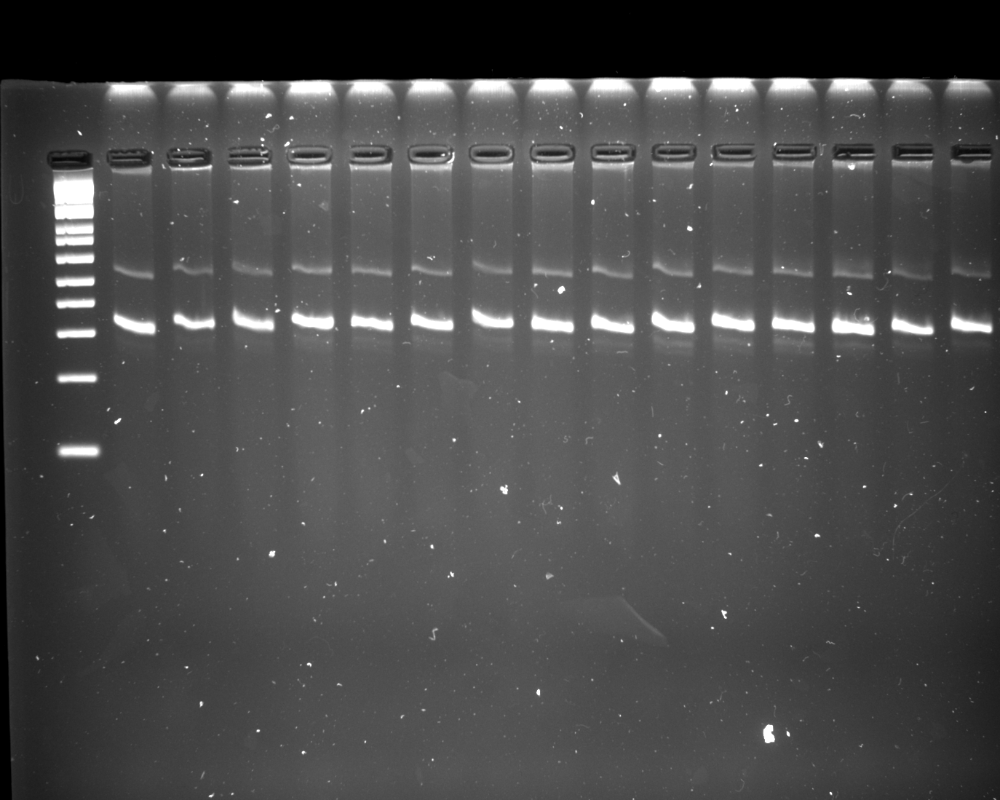

Supplement: Supplementary file 12 — Source Data [file 41467_2023_38273_MOESM12_ESM.zip › Source Data/Uncropped images/Fig7h_SYNGAP1_gel2.png]

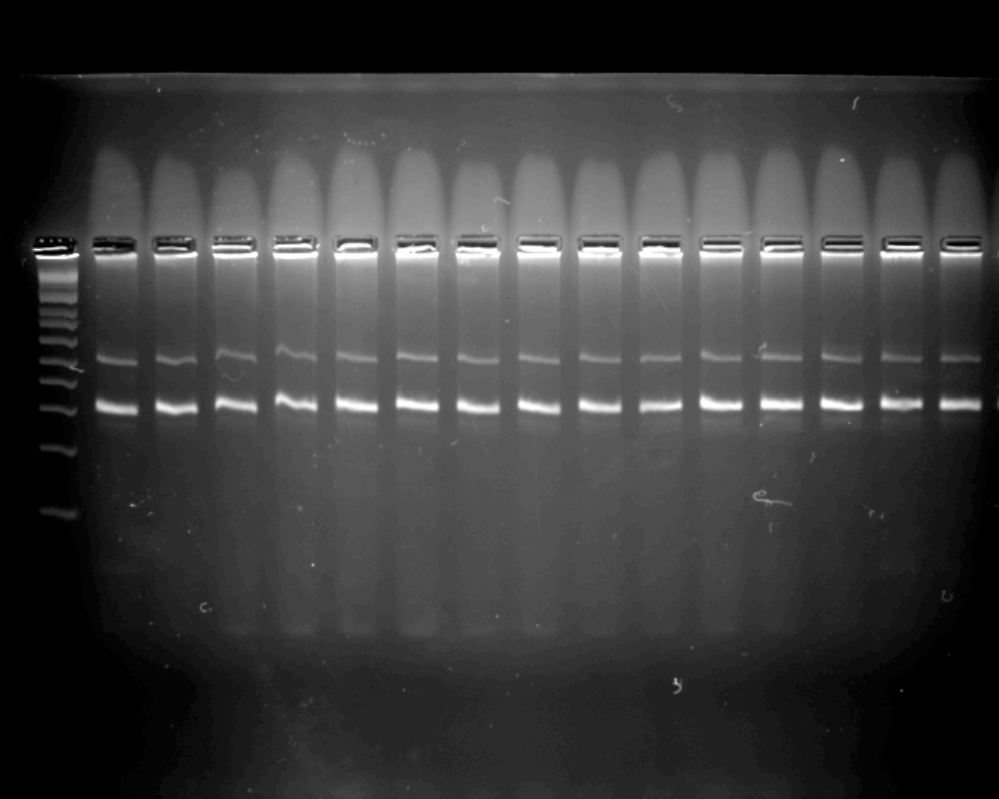

Supplement: Supplementary file 12 — Source Data [file 41467_2023_38273_MOESM12_ESM.zip › Source Data/Uncropped images/Fig7i_SYNGAP1_gel1_part1.tif]

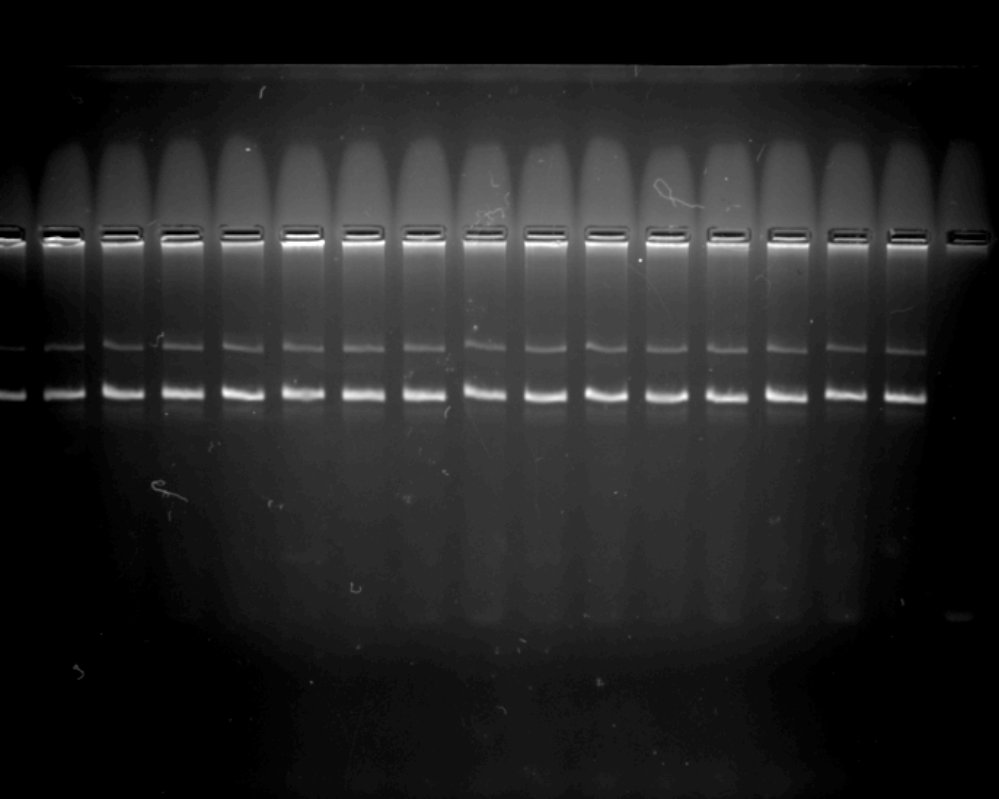

Supplement: Supplementary file 12 — Source Data [file 41467_2023_38273_MOESM12_ESM.zip › Source Data/Uncropped images/Fig7i_SYNGAP1_gel1_part2.tif]

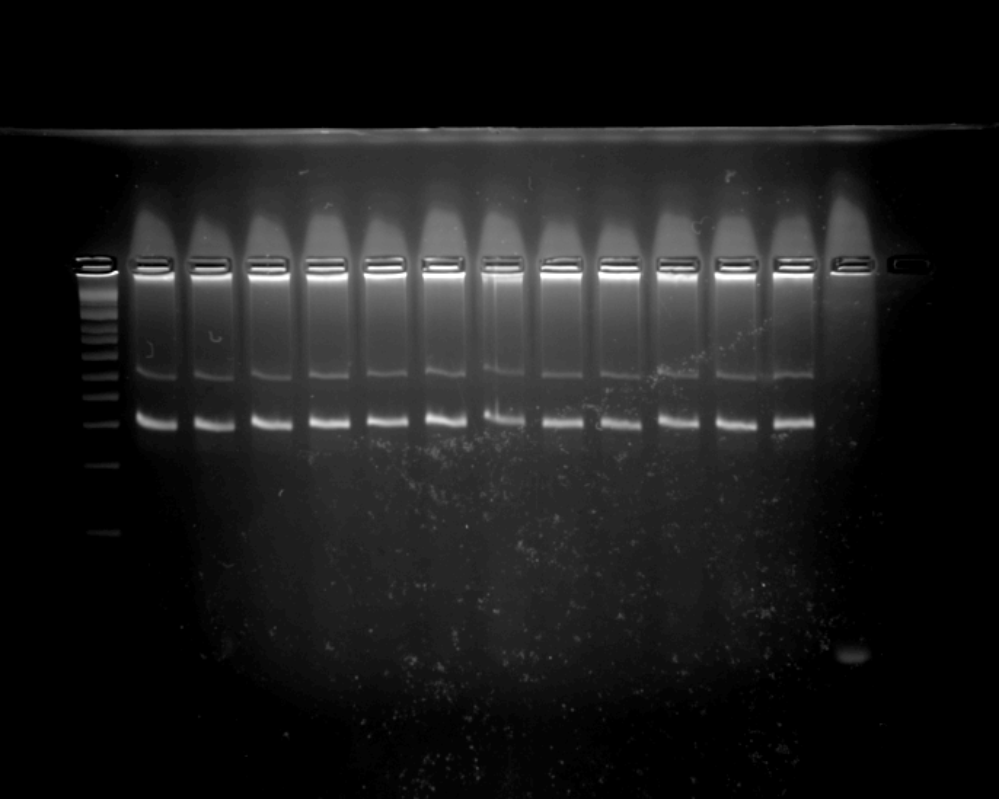

Supplement: Supplementary file 12 — Source Data [file 41467_2023_38273_MOESM12_ESM.zip › Source Data/Uncropped images/Fig7i_SYNGAP1_gel2.tif]

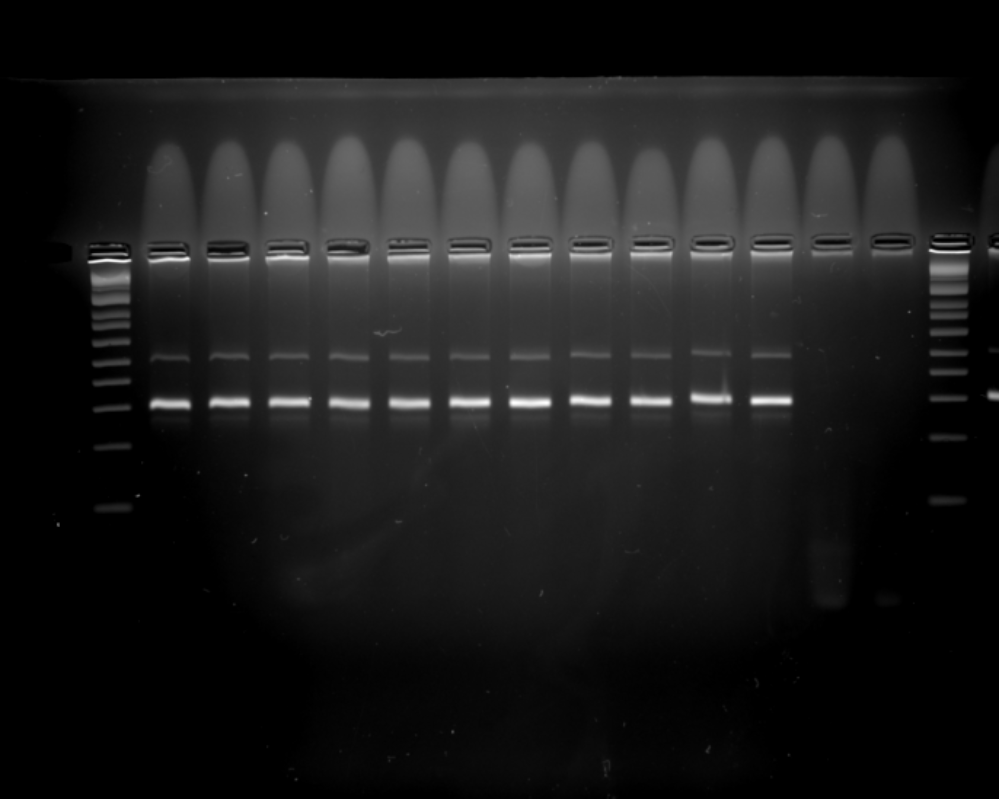

Supplement: Supplementary file 12 — Source Data [file 41467_2023_38273_MOESM12_ESM.zip › Source Data/Uncropped images/Fig7i_SYNGAP1_gel3.tif]

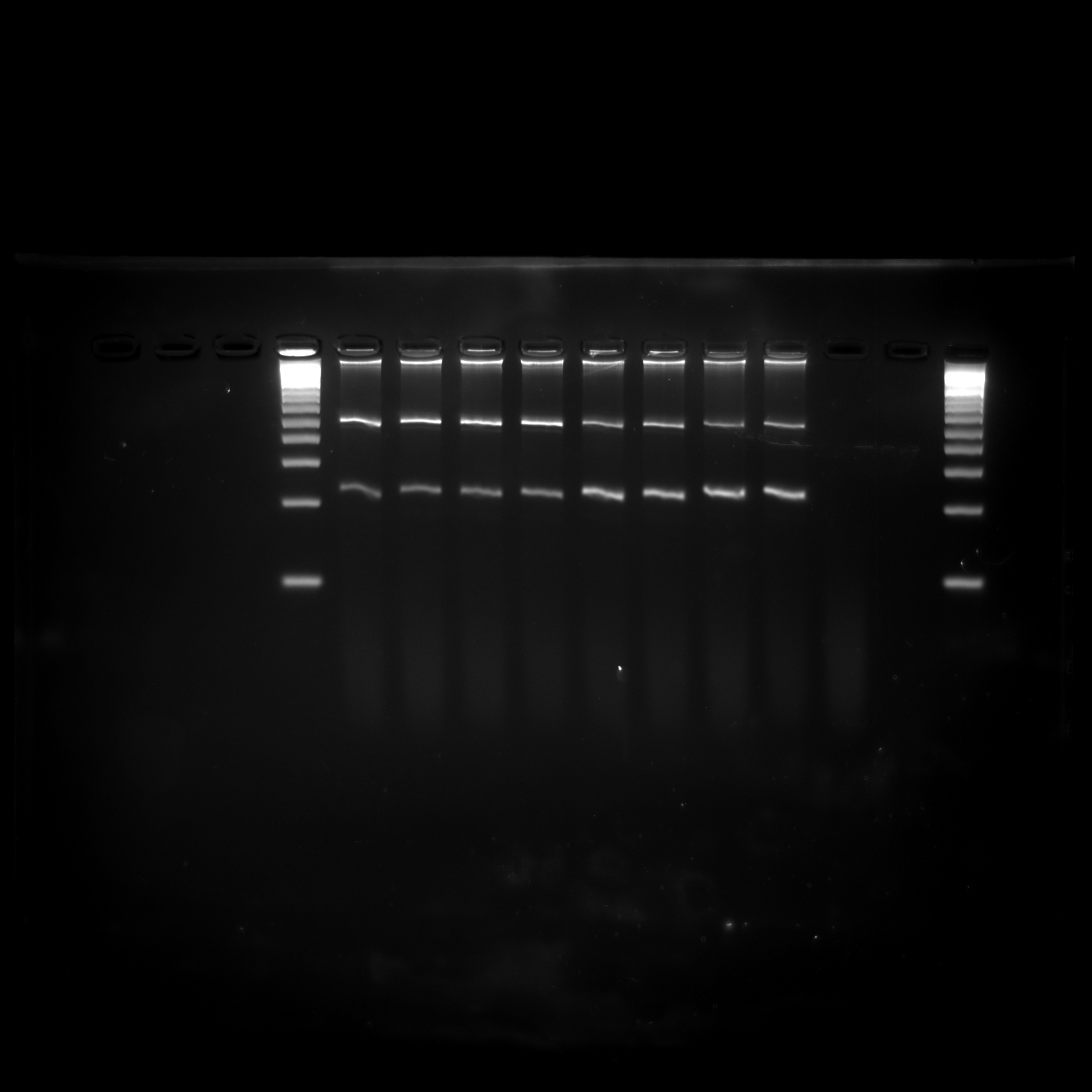

Supplement: Supplementary file 12 — Source Data [file 41467_2023_38273_MOESM12_ESM.zip › Source Data/Uncropped images/FigS4a_Syngap1.tif]

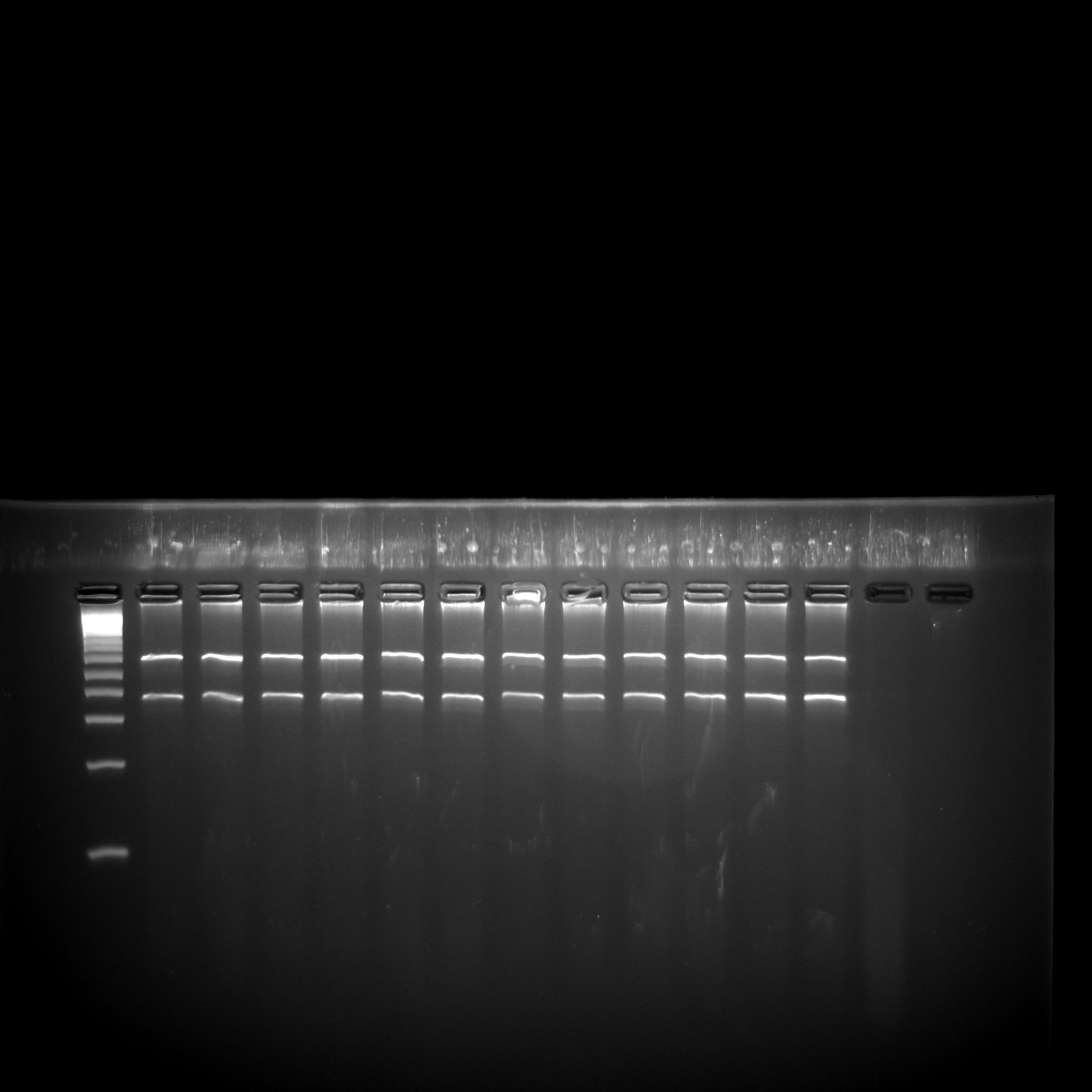

Supplement: Supplementary file 12 — Source Data [file 41467_2023_38273_MOESM12_ESM.zip › Source Data/Uncropped images/FigS4b_SYNGAP1.tif]

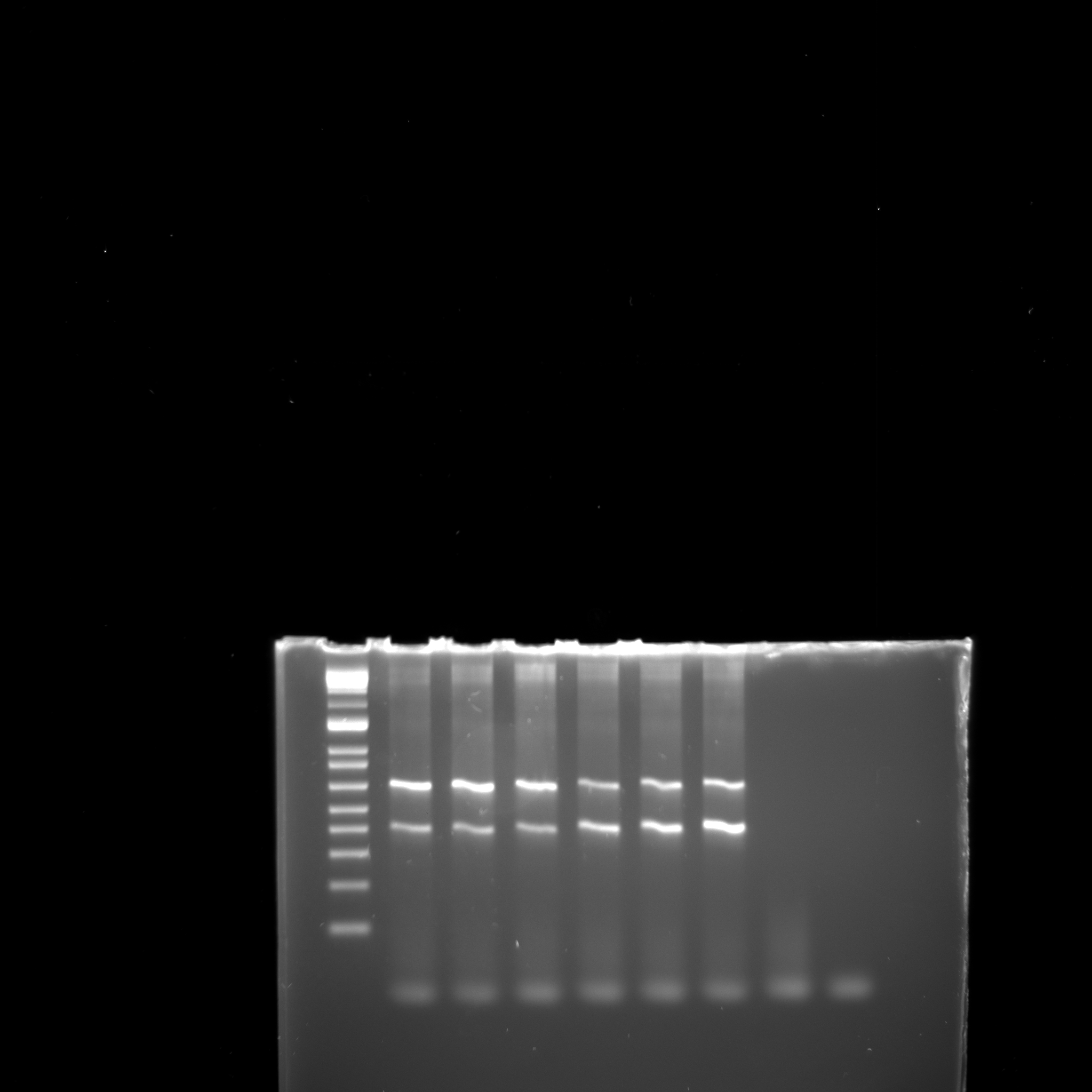

Supplement: Supplementary file 12 — Source Data [file 41467_2023_38273_MOESM12_ESM.zip › Source Data/Uncropped images/FigS4c_SYNGAP1_leftpanel_part1.tif]

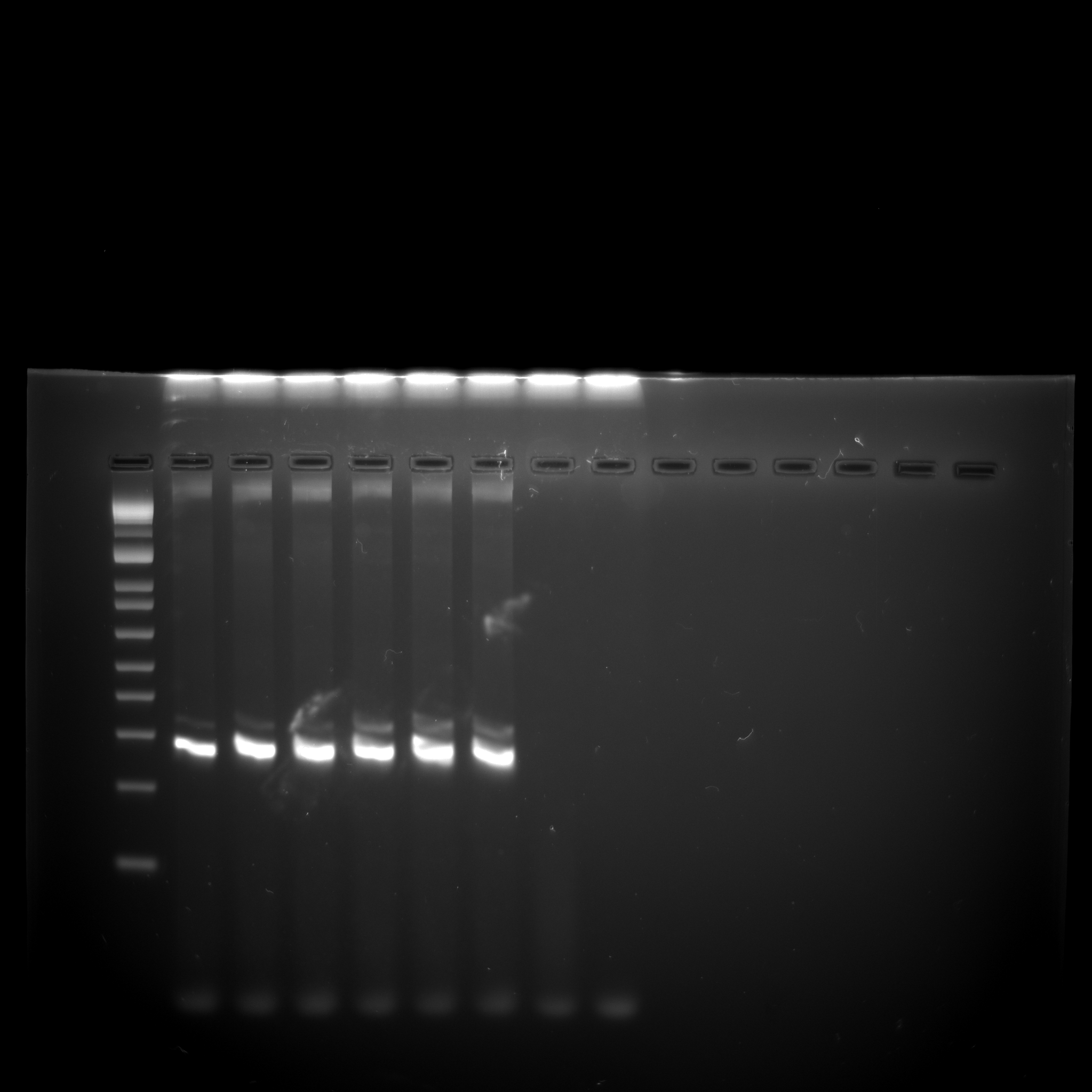

Supplement: Supplementary file 12 — Source Data [file 41467_2023_38273_MOESM12_ESM.zip › Source Data/Uncropped images/FigS4c_SYNGAP1_middlepanel_part1.tif]

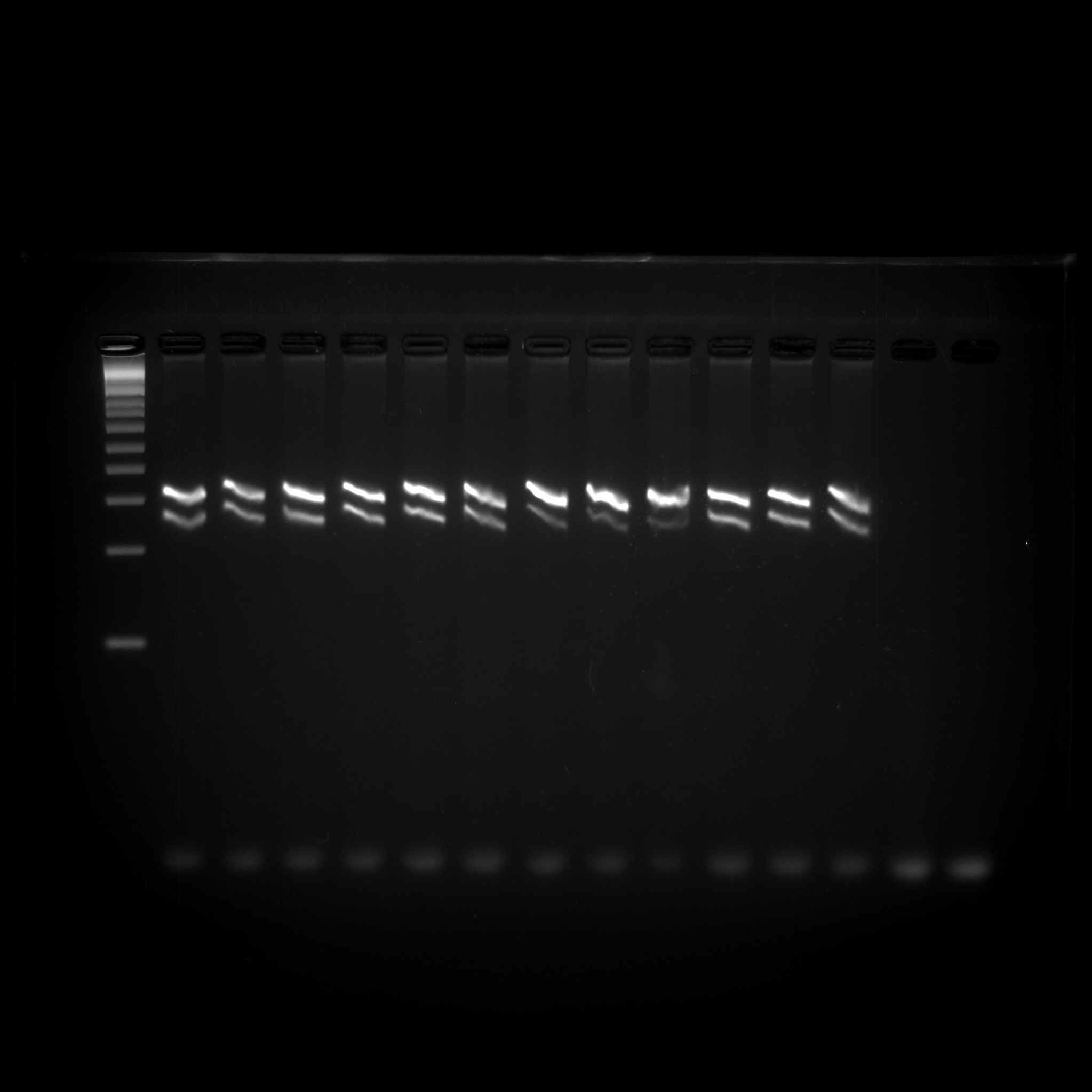

Supplement: Supplementary file 12 — Source Data [file 41467_2023_38273_MOESM12_ESM.zip › Source Data/Uncropped images/FigS4c_SYNGAP1_middlepanel_part2.tif]

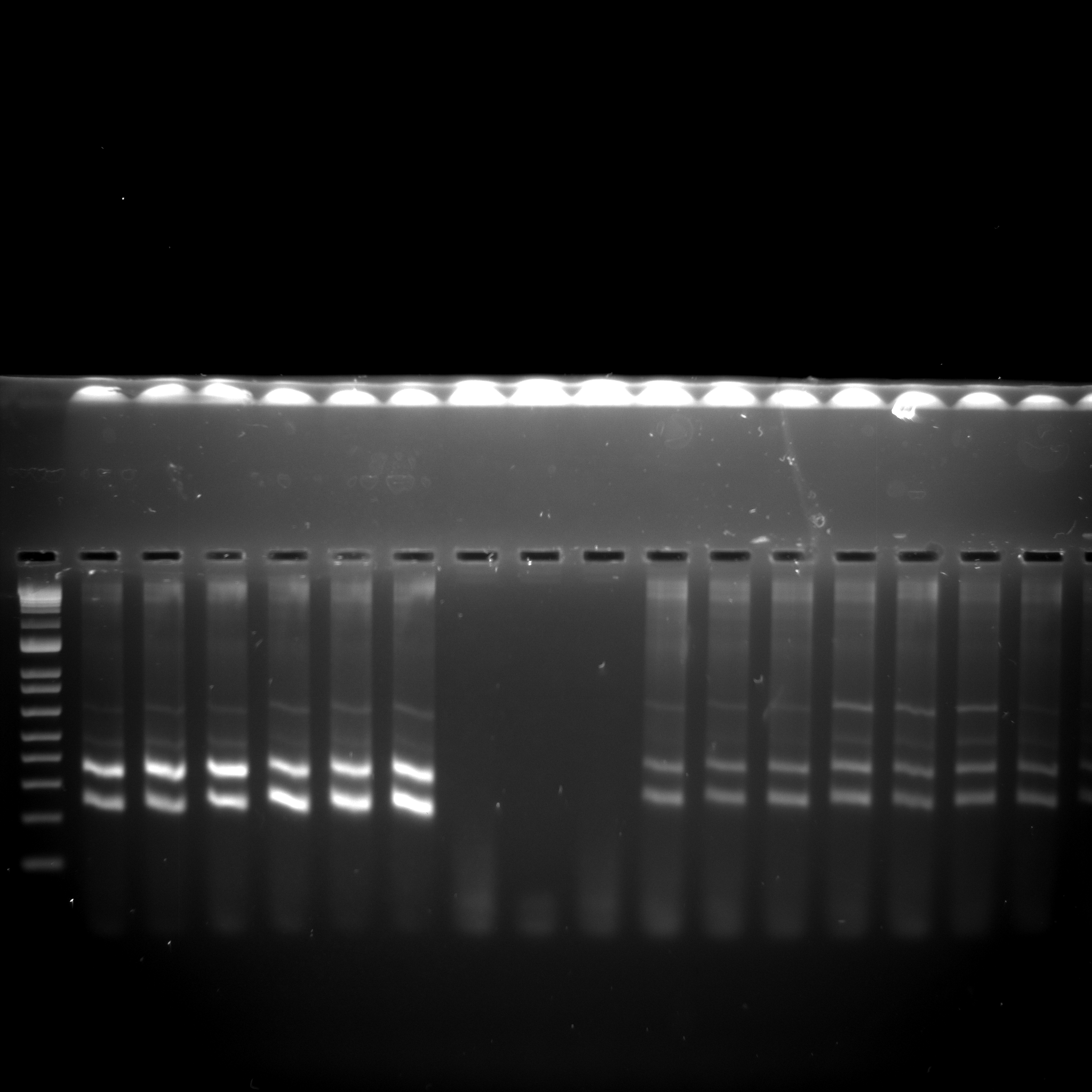

Supplement: Supplementary file 12 — Source Data [file 41467_2023_38273_MOESM12_ESM.zip › Source Data/Uncropped images/FigS4c_SYNGAP1_rightpanel_part1.tif]

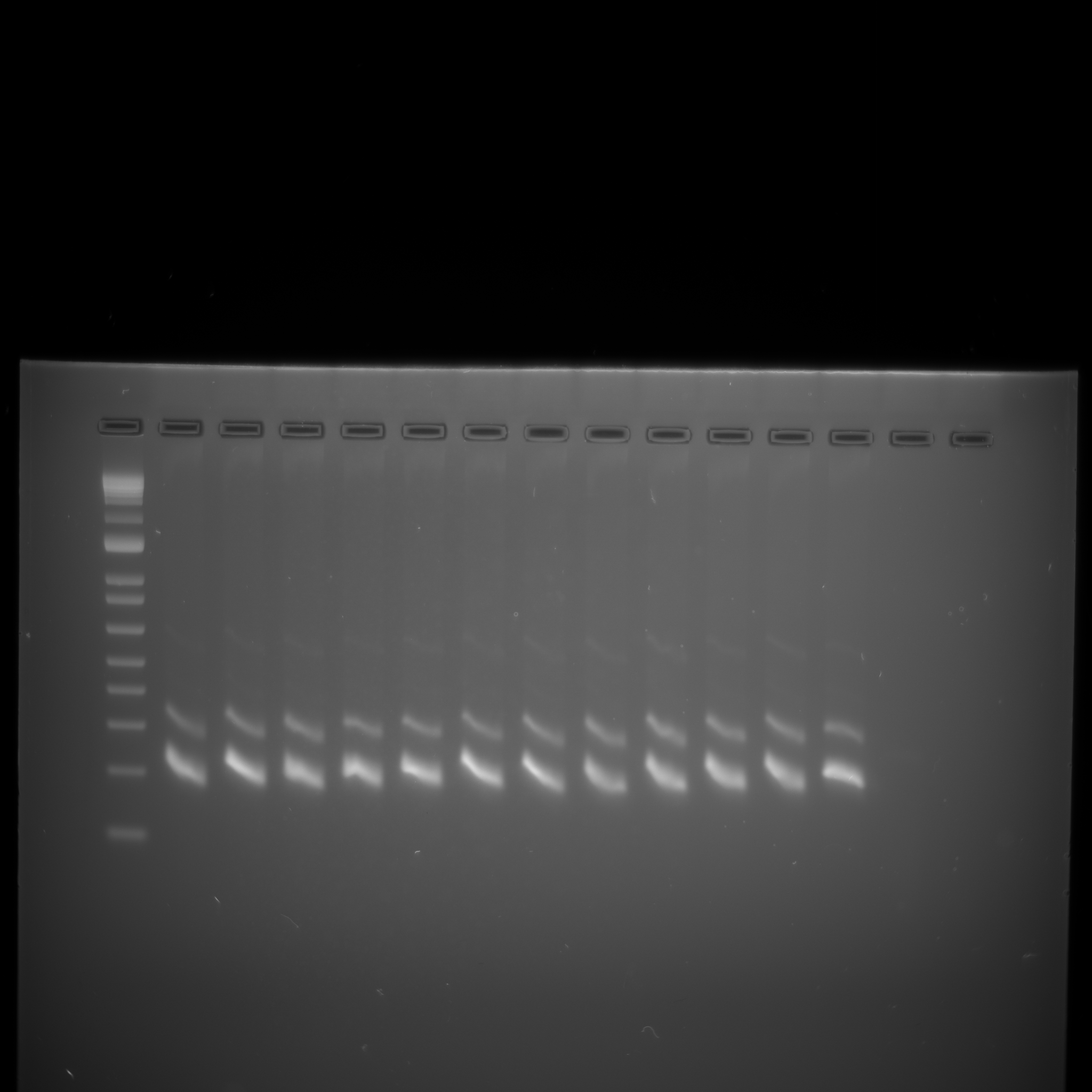

Supplement: Supplementary file 12 — Source Data [file 41467_2023_38273_MOESM12_ESM.zip › Source Data/Uncropped images/FigS4c_SYNGAP1_rightpanel_part2.tif]

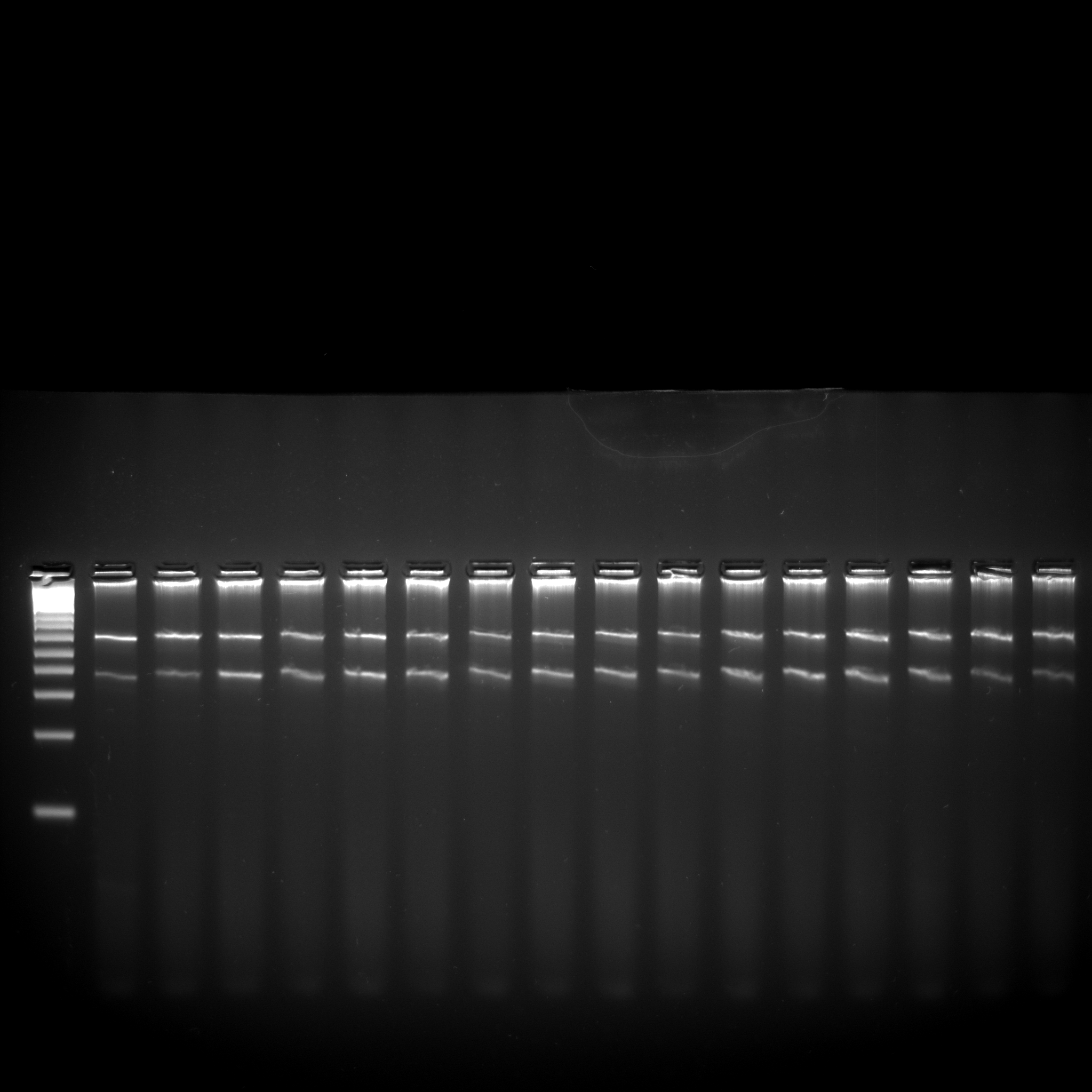

Supplement: Supplementary file 12 — Source Data [file 41467_2023_38273_MOESM12_ESM.zip › Source Data/Uncropped images/FigS4d_SYNGAP1.tif]

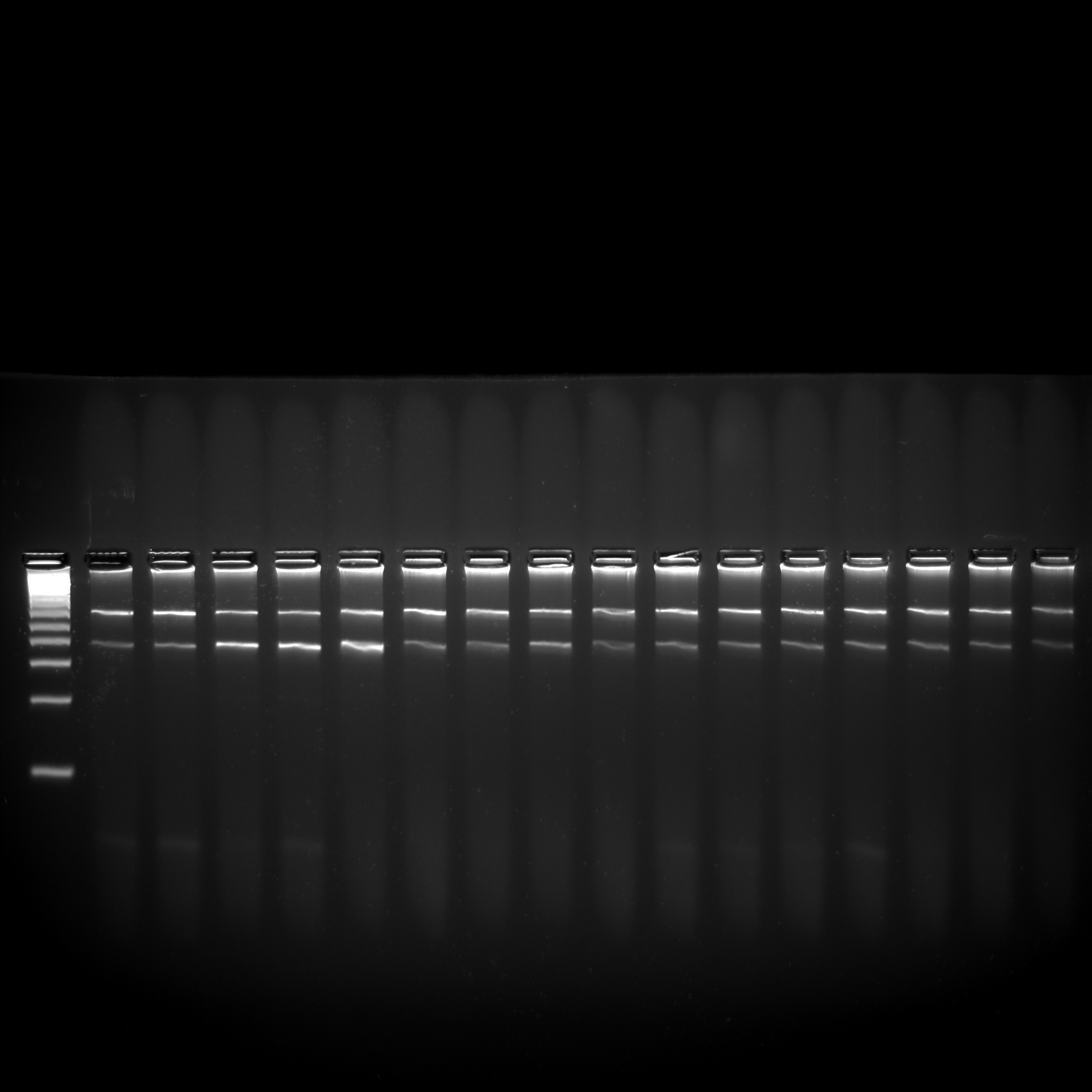

Supplement: Supplementary file 12 — Source Data [file 41467_2023_38273_MOESM12_ESM.zip › Source Data/Uncropped images/FigS4e_SYNGAP1_part1.tif]

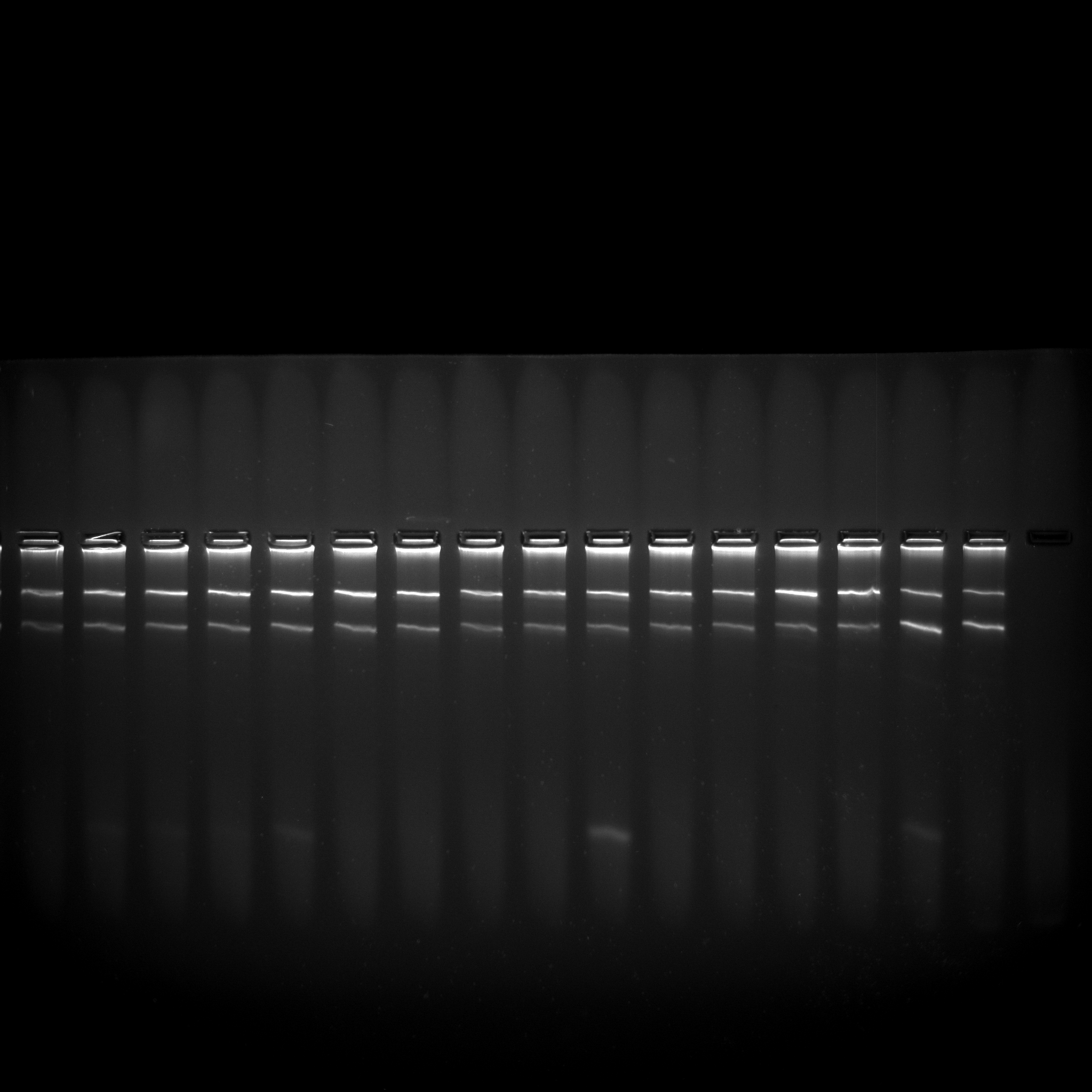

Supplement: Supplementary file 12 — Source Data [file 41467_2023_38273_MOESM12_ESM.zip › Source Data/Uncropped images/FigS4e_SYNGAP1_part2.tif]

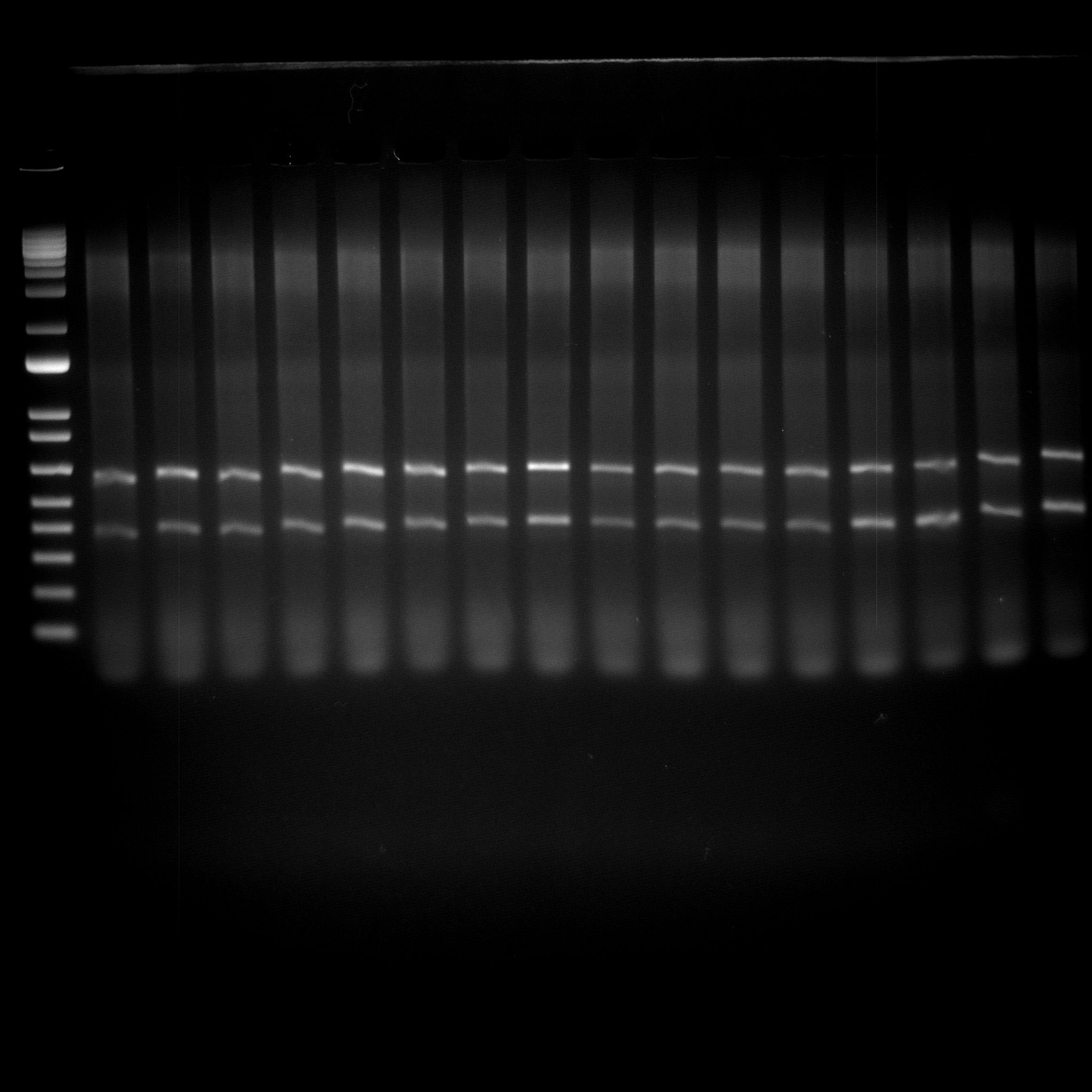

Supplement: Supplementary file 12 — Source Data [file 41467_2023_38273_MOESM12_ESM.zip › Source Data/Uncropped images/FigS5b_SYNGAP1_part1.tif]

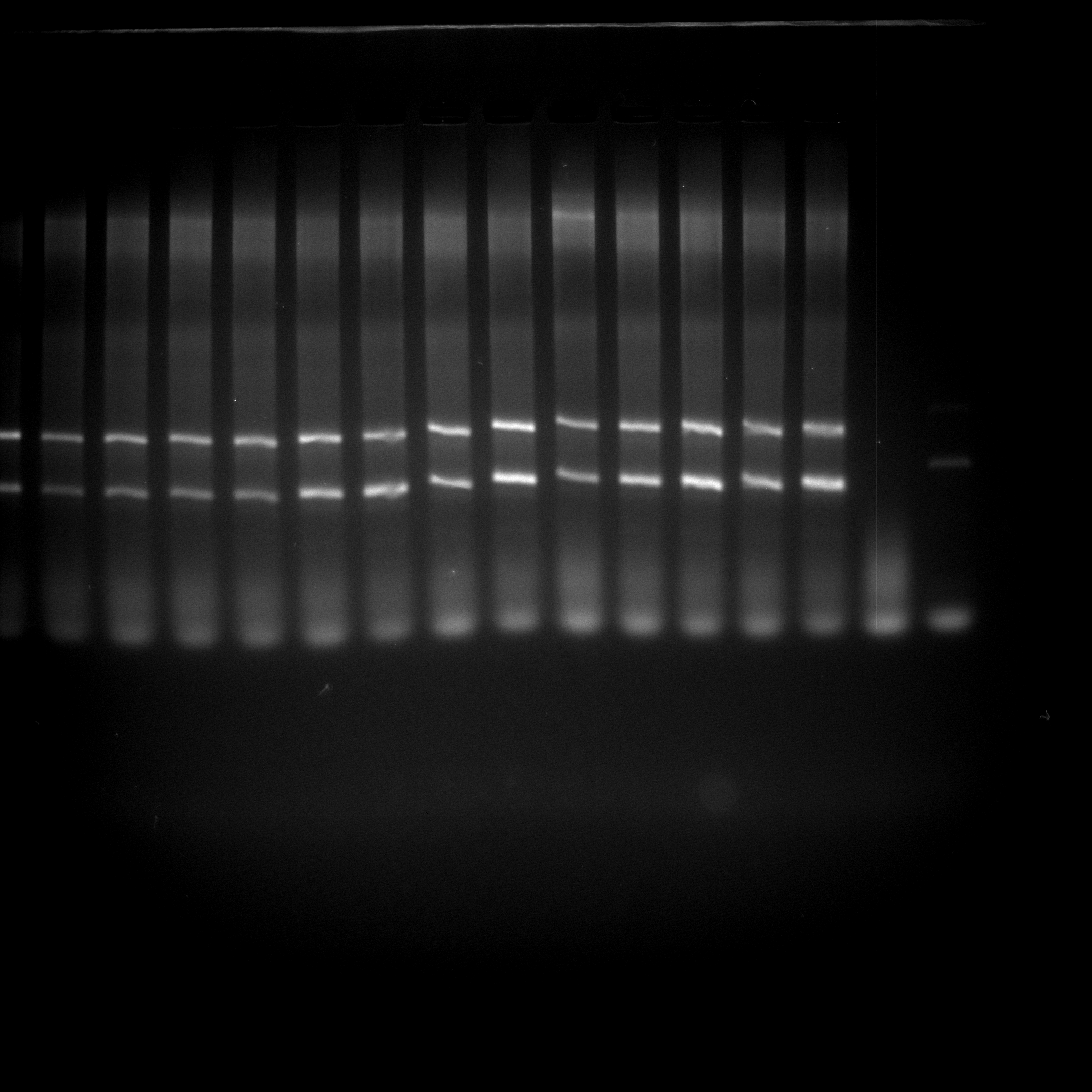

Supplement: Supplementary file 12 — Source Data [file 41467_2023_38273_MOESM12_ESM.zip › Source Data/Uncropped images/FigS5b_SYNGAP1_part2.tif]

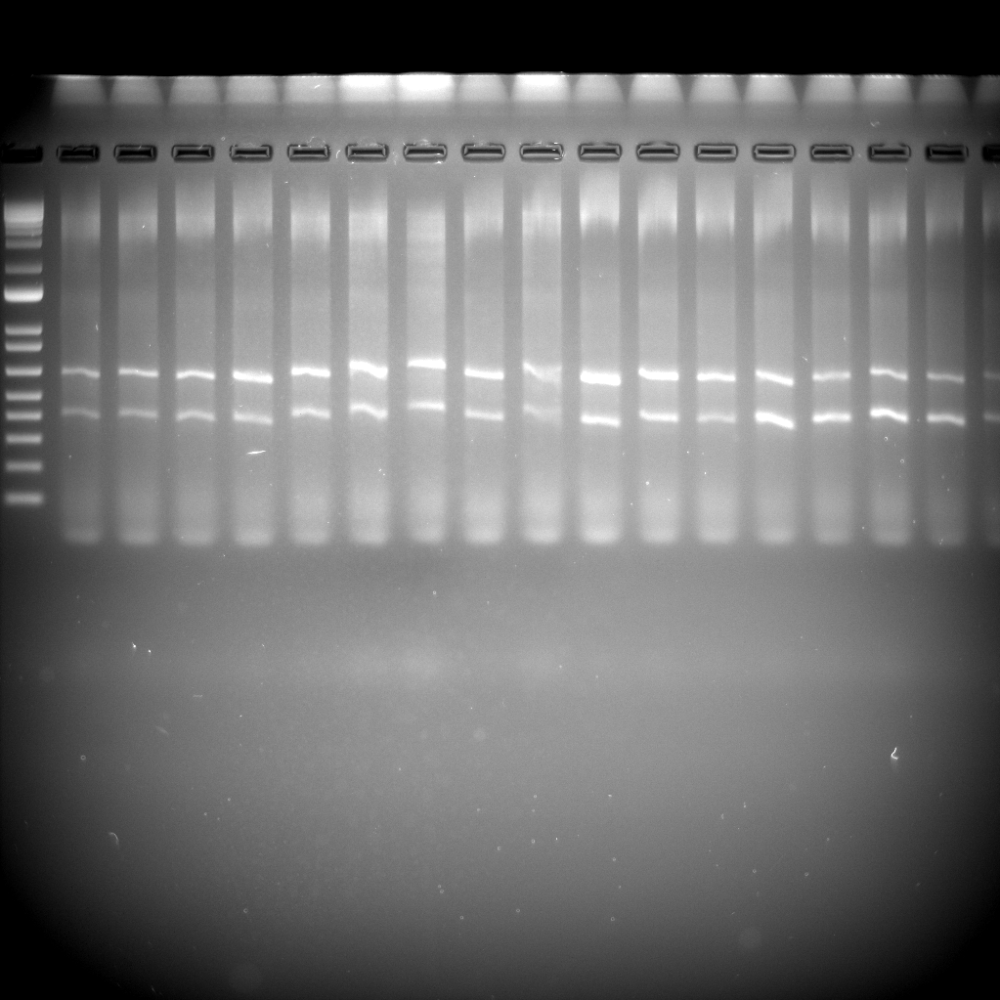

Supplement: Supplementary file 12 — Source Data [file 41467_2023_38273_MOESM12_ESM.zip › Source Data/Uncropped images/FigS5c_SYNGAP1_part1.tif]

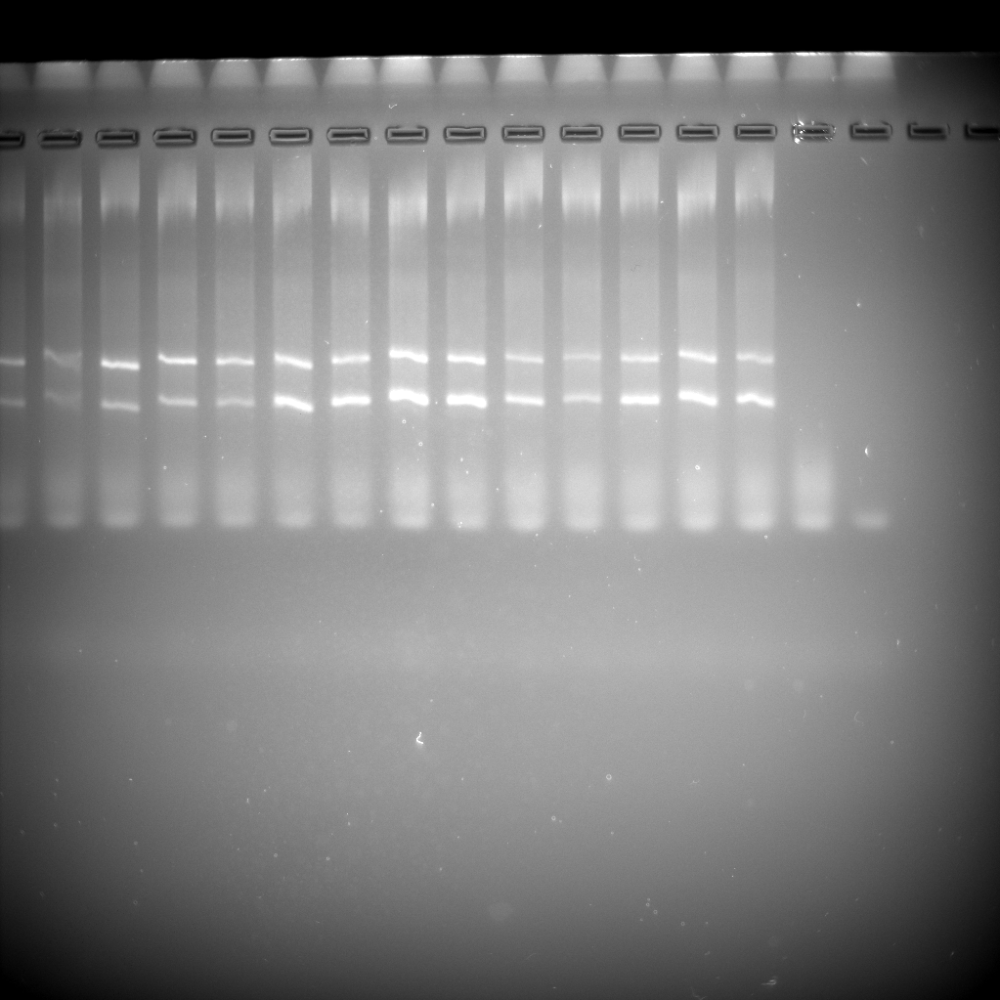

Supplement: Supplementary file 12 — Source Data [file 41467_2023_38273_MOESM12_ESM.zip › Source Data/Uncropped images/FigS5c_SYNGAP1_part2.tif]

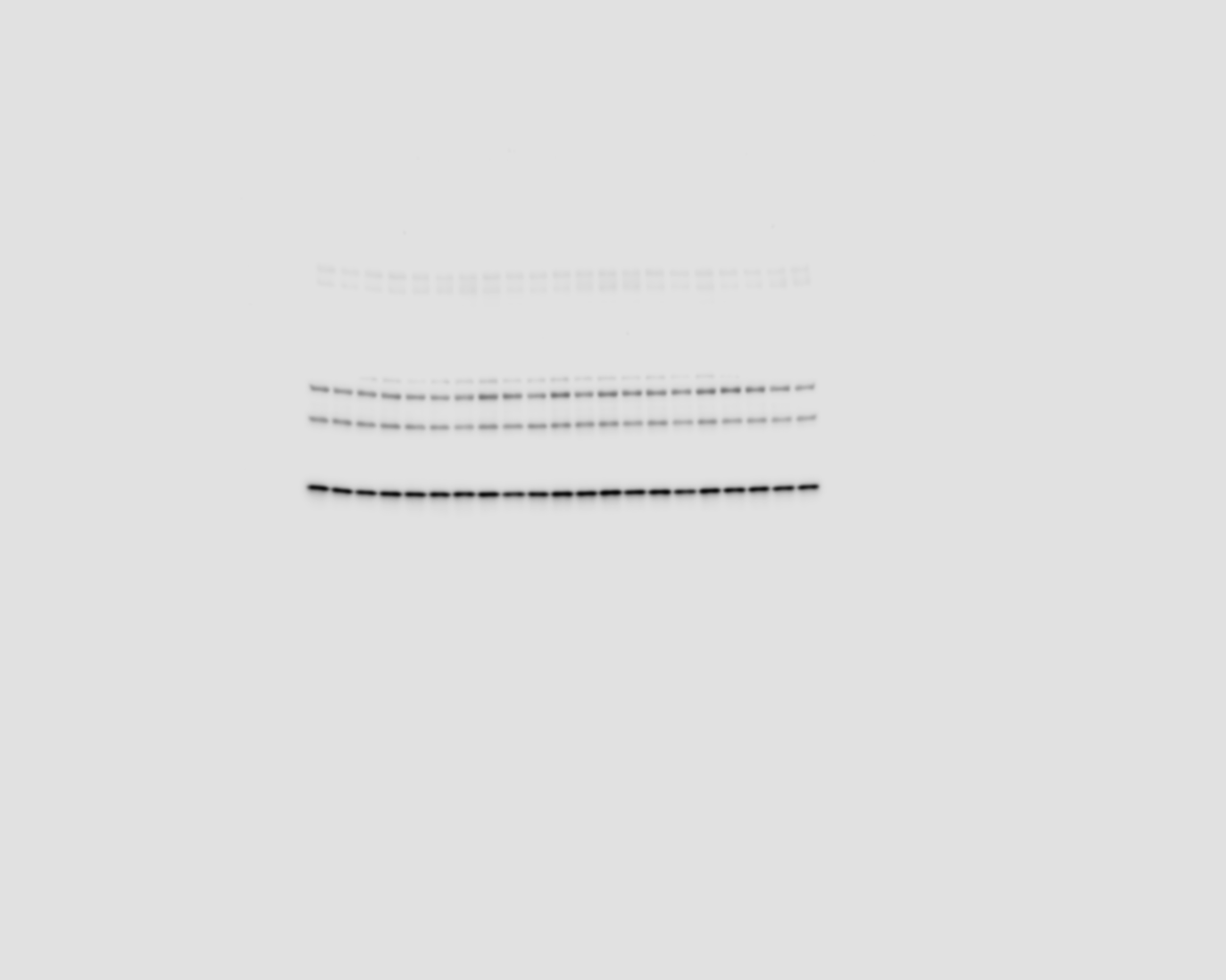

Supplement: Supplementary file 12 — Source Data [file 41467_2023_38273_MOESM12_ESM.zip › Source Data/Uncropped images/FigS5d_SYNGAP1blot_ATP5F1.tif]

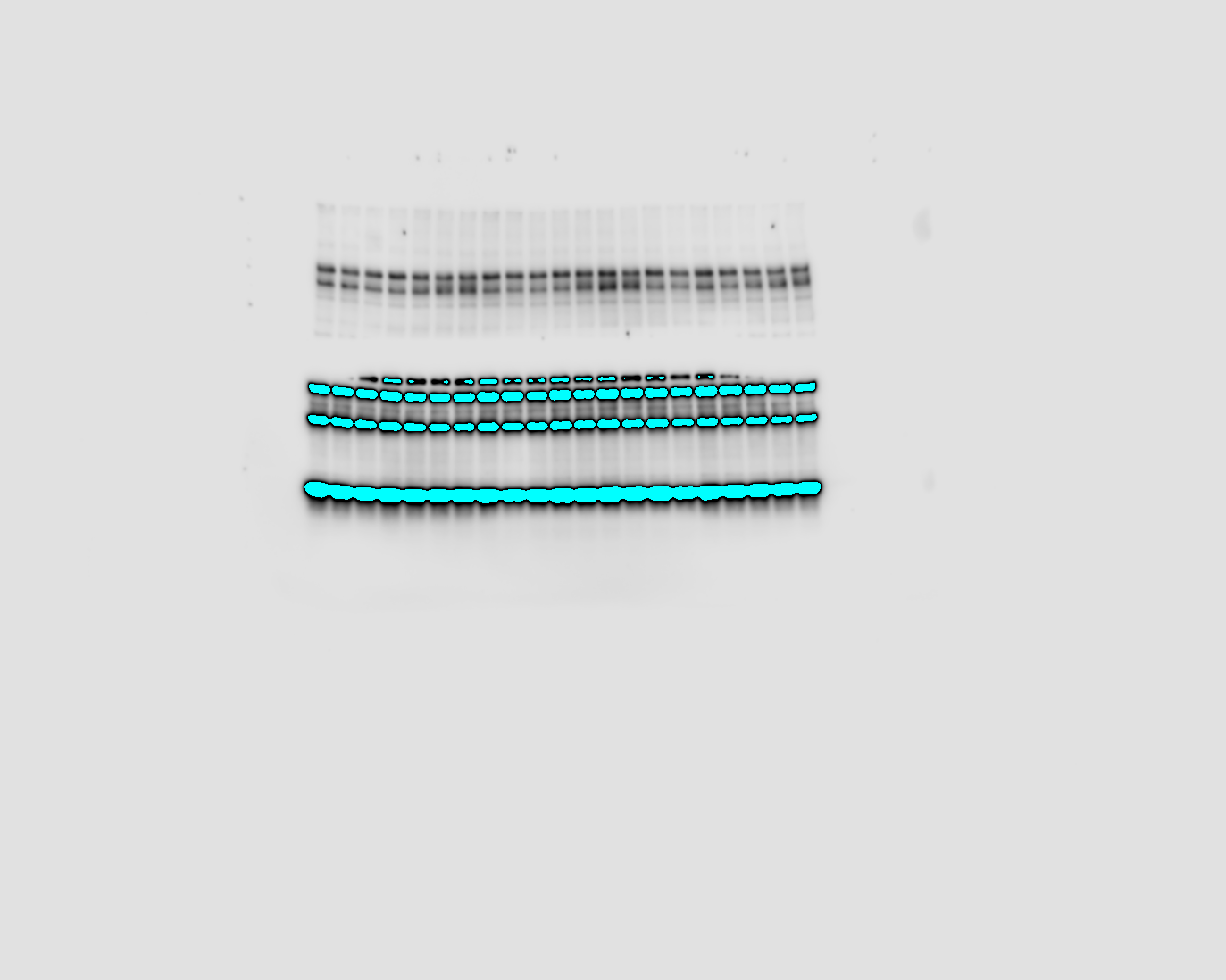

Supplement: Supplementary file 12 — Source Data [file 41467_2023_38273_MOESM12_ESM.zip › Source Data/Uncropped images/FigS5d_SYNGAP1blot_SYNGAP1.tif]

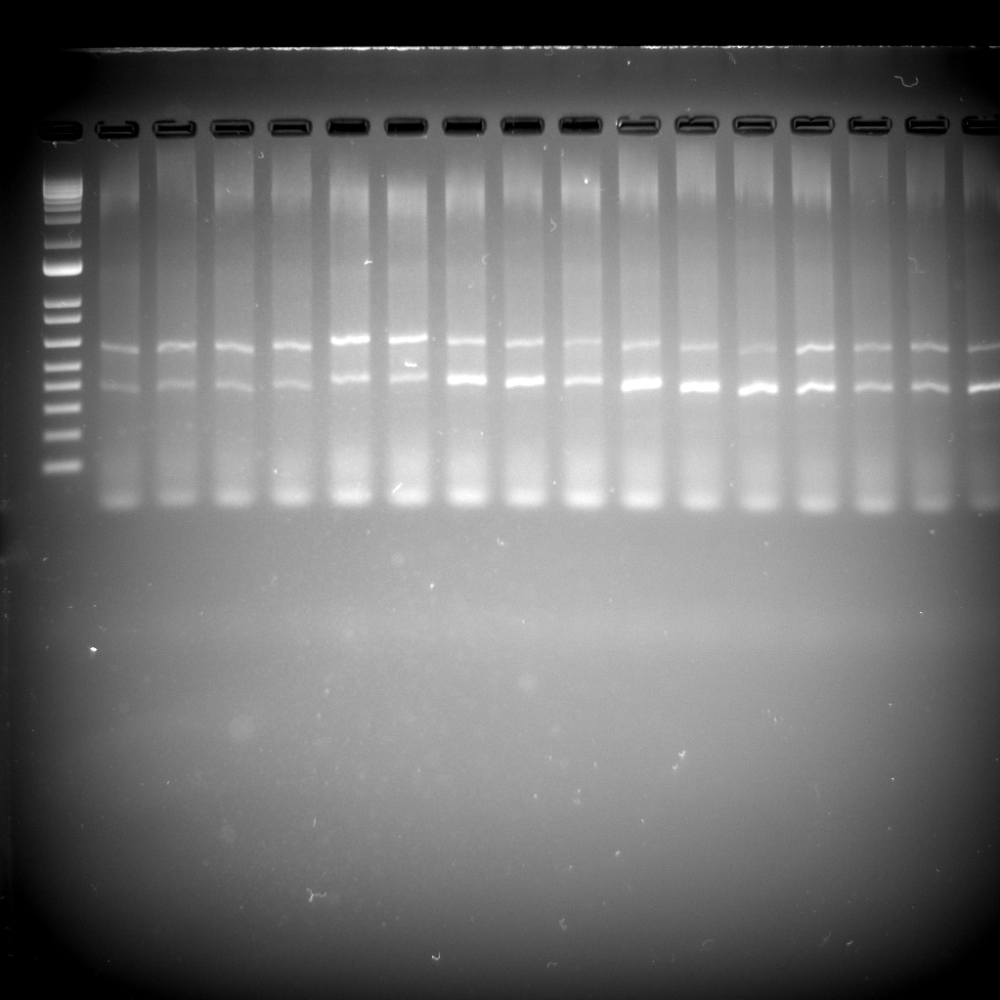

Supplement: Supplementary file 12 — Source Data [file 41467_2023_38273_MOESM12_ESM.zip › Source Data/Uncropped images/FigS5e_SYNGAP1_part1.tif]

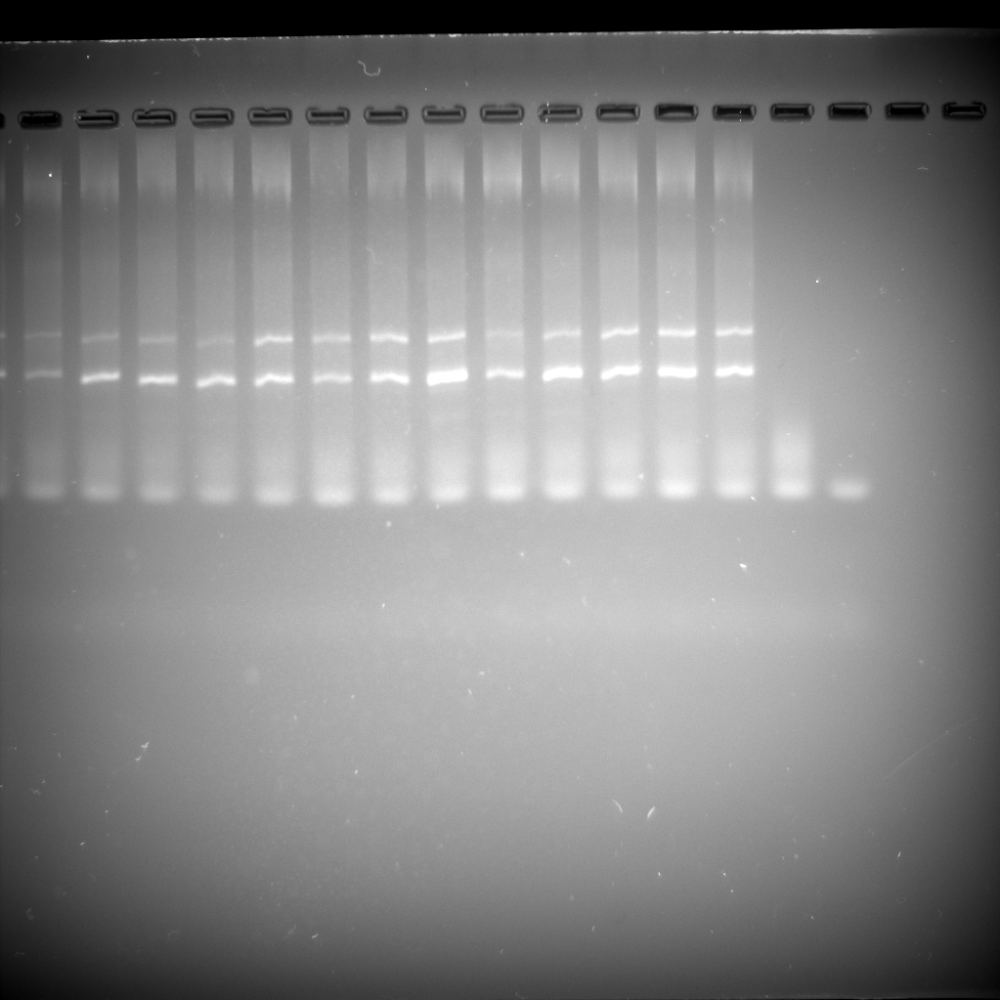

Supplement: Supplementary file 12 — Source Data [file 41467_2023_38273_MOESM12_ESM.zip › Source Data/Uncropped images/FigS5e_SYNGAP1_part2.tif]

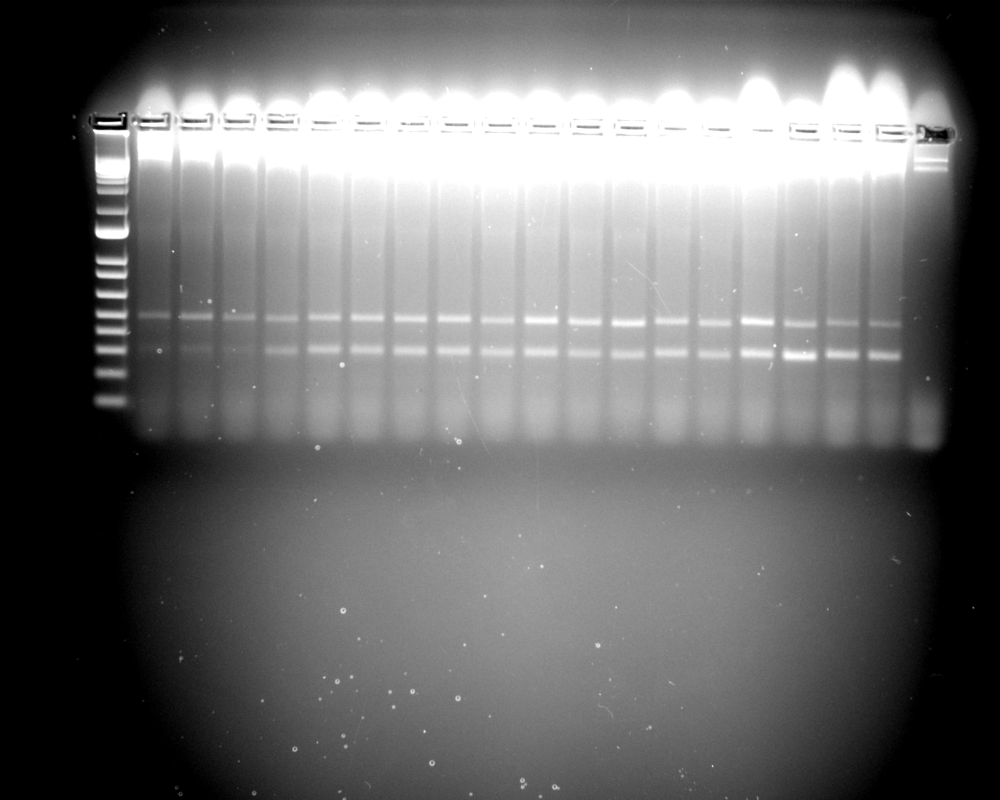

Supplement: Supplementary file 12 — Source Data [file 41467_2023_38273_MOESM12_ESM.zip › Source Data/Uncropped images/FigS5f_SYNGAP1.tif]

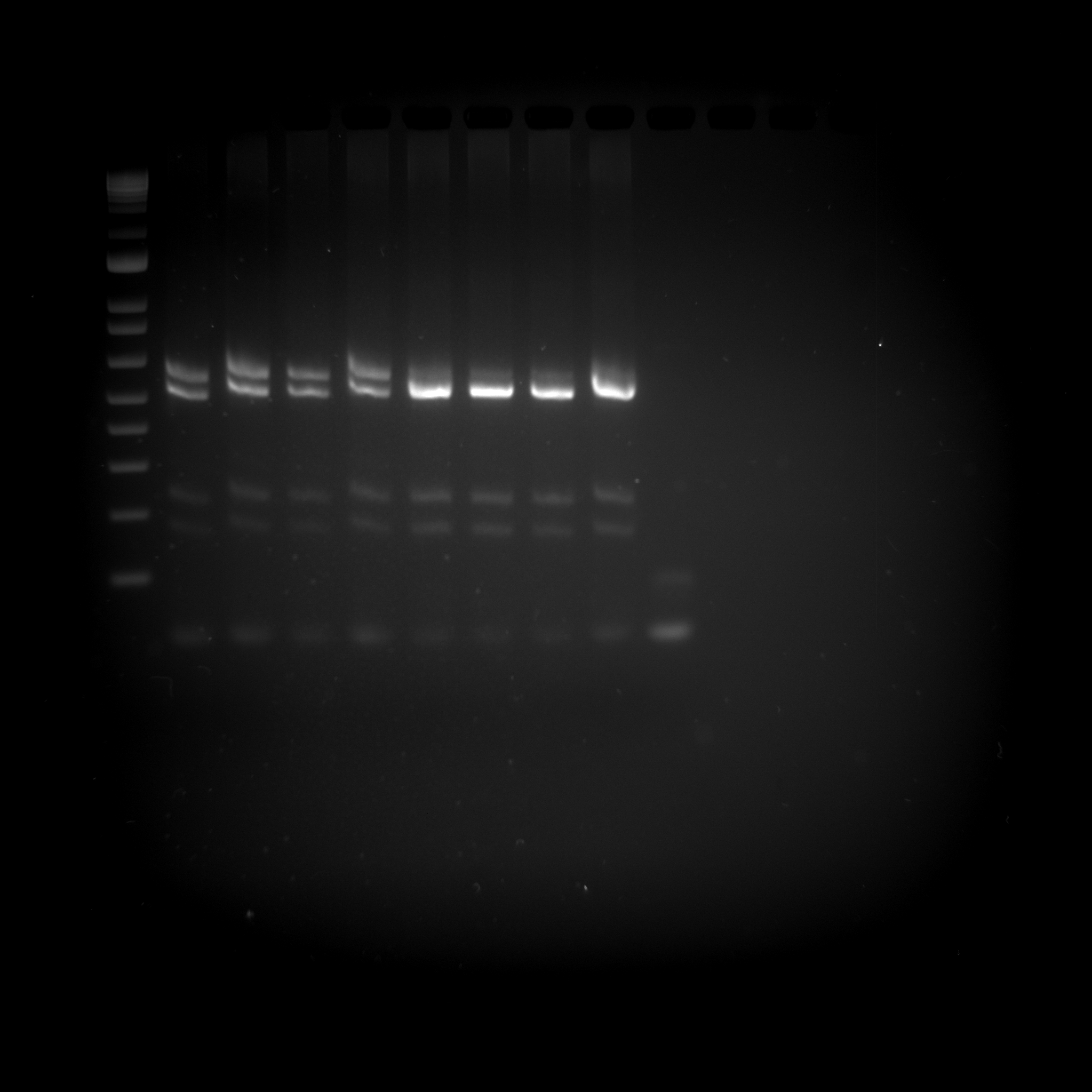

Supplement: Supplementary file 12 — Source Data [file 41467_2023_38273_MOESM12_ESM.zip › Source Data/Uncropped images/FigS7c_Scn1a.tif]

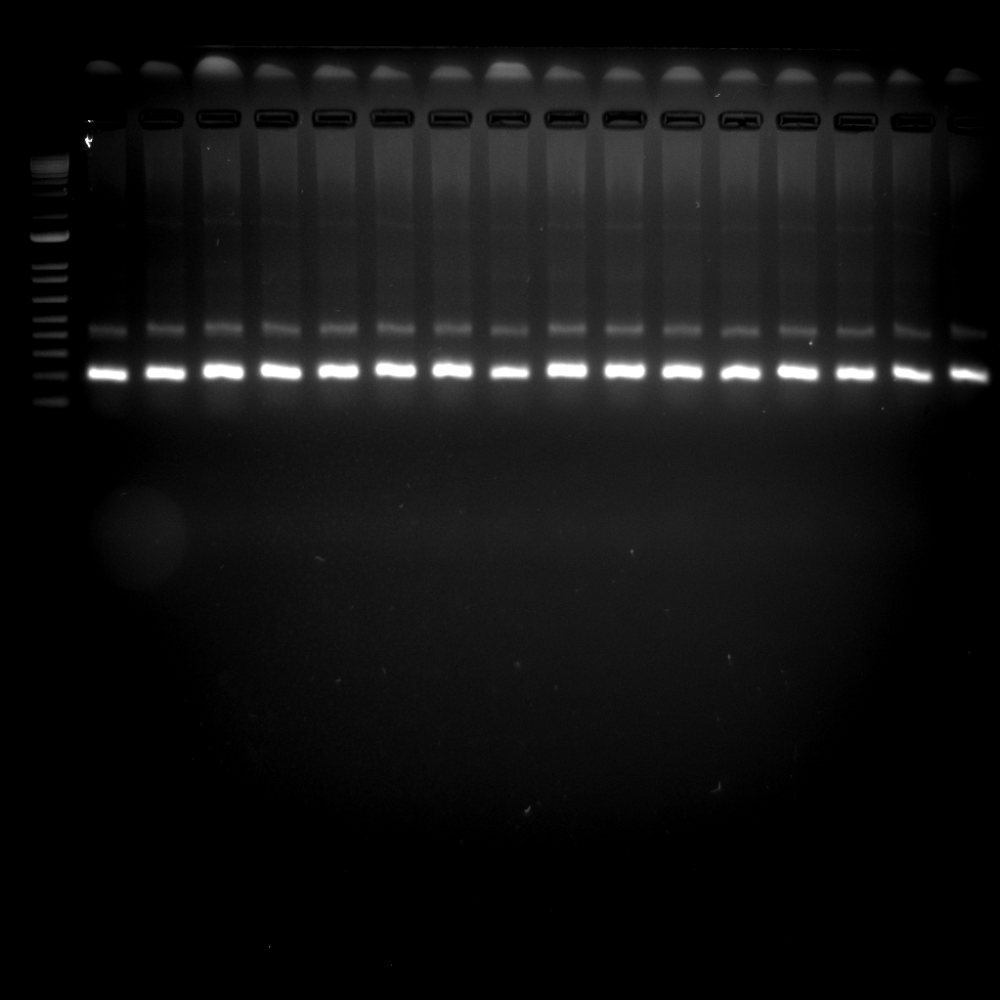

Supplement: Supplementary file 12 — Source Data [file 41467_2023_38273_MOESM12_ESM.zip › Source Data/Uncropped images/FigS7e_Syngap1_gel1_part1.tif]

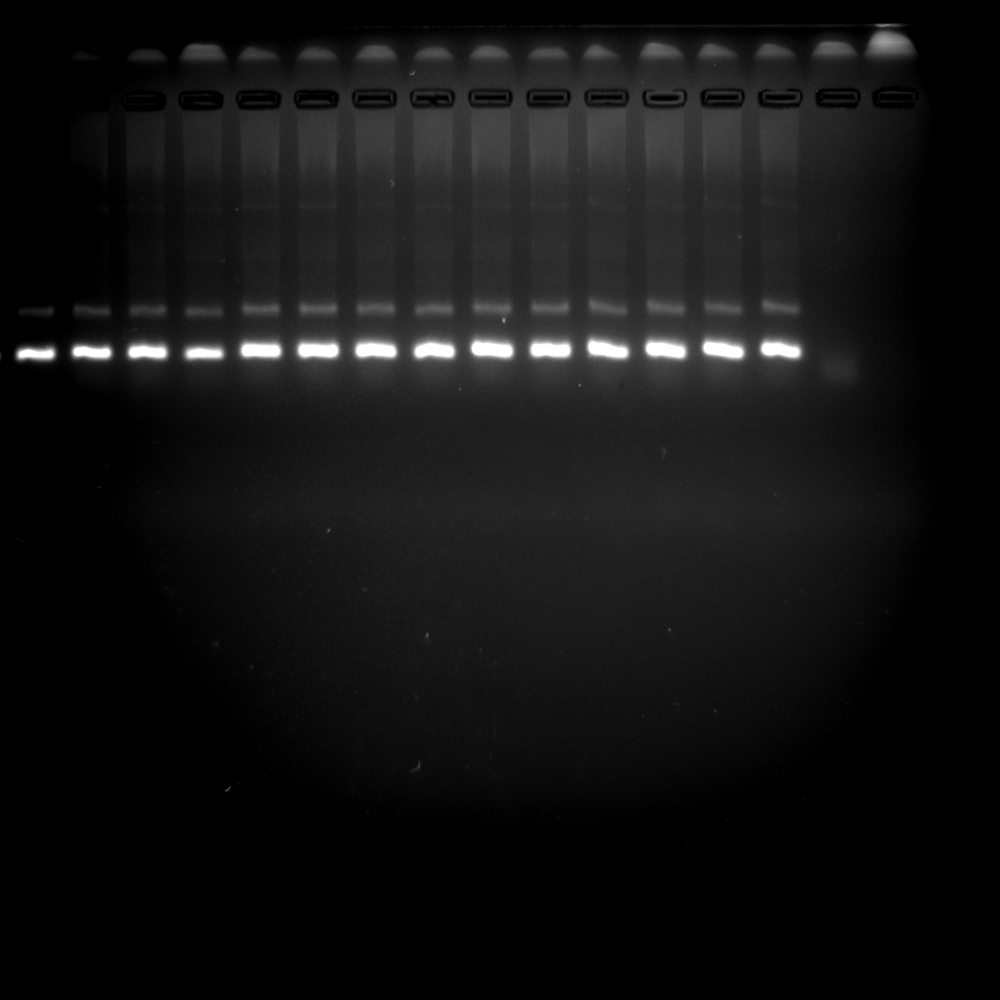

Supplement: Supplementary file 12 — Source Data [file 41467_2023_38273_MOESM12_ESM.zip › Source Data/Uncropped images/FigS7e_Syngap1_gel1_part2.tif]

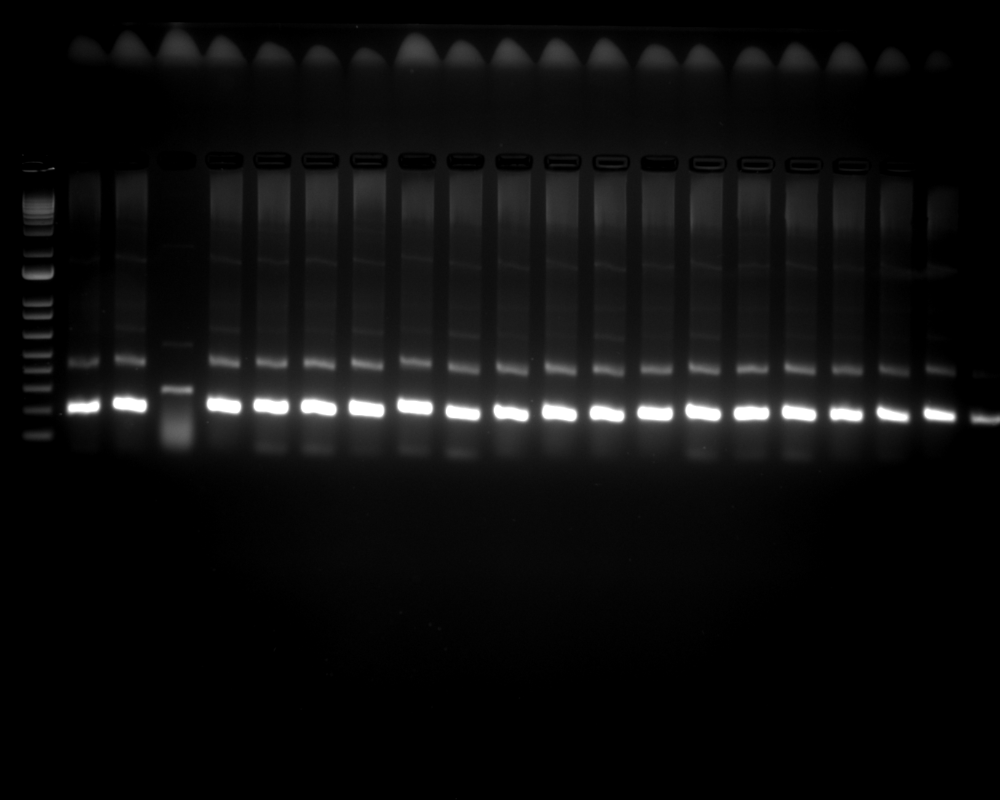

Supplement: Supplementary file 12 — Source Data [file 41467_2023_38273_MOESM12_ESM.zip › Source Data/Uncropped images/FigS7e_Syngap1_gel2.tif]
